# Machine intelligence-accelerated discovery of all-natural plastic substitutes

**DOI:** 10.1038/s41565-024-01635-z

**Published:** 2024-03-18

**Authors:** Tianle Chen, Zhenqian Pang, Shuaiming He, Yang Li, Snehi Shrestha, Joshua M. Little, Haochen Yang, Tsai-Chun Chung, Jiayue Sun, Hayden Christopher Whitley, I-Chi Lee, Taylor J. Woehl, Teng Li, Liangbing Hu, Po-Yen Chen

**Affiliations:** 1 Department of Chemical and Biomolecular Engineering, University of Maryland, College Park, MD USA; 2 Department of Mechanical Engineering, University of Maryland, College Park, MD USA; 3 Department of Chemistry and Biochemistry, University of Maryland, College Park, MD USA; 4 Department of Biomedical Engineering and Environmental Sciences, National Tsing Hua University, Hsinchu, Taiwan; 5 Department of Materials Science and Engineering, University of Maryland, College Park, MD USA; 6 Maryland Robotics Center, College Park, MD USA

**Keywords:** Composites, Design, synthesis and processing

## Abstract

One possible solution against the accumulation of petrochemical plastics in natural environments is to develop biodegradable plastic substitutes using natural components. However, discovering all-natural alternatives that meet specific properties, such as optical transparency, fire retardancy and mechanical resilience, which have made petrochemical plastics successful, remains challenging. Current approaches still rely on iterative optimization experiments. Here we show an integrated workflow that combines robotics and machine learning to accelerate the discovery of all-natural plastic substitutes with programmable optical, thermal and mechanical properties. First, an automated pipetting robot is commanded to prepare 286 nanocomposite films with various properties to train a support-vector machine classifier. Next, through 14 active learning loops with data augmentation, 135 all-natural nanocomposites are fabricated stagewise, establishing an artificial neural network prediction model. We demonstrate that the prediction model can conduct a two-way design task: (1) predicting the physicochemical properties of an all-natural nanocomposite from its composition and (2) automating the inverse design of biodegradable plastic substitutes that fulfils various user-specific requirements. By harnessing the model’s prediction capabilities, we prepare several all-natural substitutes, that could replace non-biodegradable counterparts as exhibiting analogous properties. Our methodology integrates robot-assisted experiments, machine intelligence and simulation tools to accelerate the discovery and design of eco-friendly plastic substitutes starting from building blocks taken from the generally-recognized-as-safe database.

## Abstract (web summary)

An integrated workflow that uses robotics and machine learning to discover all-natural plastic substitutes with programmable properties is presented.

## Main

Petrochemical plastics are lightweight, durable and inexpensive, enabling almost ubiquitous applications^1^. However, less than 10% of petrochemical plastics can be recycled, and nearly 80% of used plastics end up in landfills or pollute the environment, resulting in global plastic pollution^2^. One promising solution is to use natural components to develop sustainable, biodegradable plastic substitutes, which can attenuate the magnitude of plastic waste and prevent the release of microplastics^3^. However, discovering biodegradable alternatives that meet specific property criteria, such as optical transparency, fire retardancy and mechanical resilience, presents substantial challenges. Current approaches rely on trial-and-error experiments and probe a broad range of parameters in a scattershot manner^4,5^. As more plastics are needed to be replaced, the time and cost required to find suitable biodegradable substitutes will increase. Additionally, biodegradable plastic substitutes typically contain multiple natural building blocks, and conventional simulation tools are not efficient to describe such complex systems. Instead, it is highly desirable to have a prediction model that can optimize multiple physicochemical properties of a biodegradable plastic substitute and automatically suggest ideal fabrication parameters^6,7^, largely accelerating the research and development processes.

Machine learning (ML) is a form of artificial intelligence (AI) that constructs a model to make predictions or recommendations across multiple degrees of freedom^8,9^. Recently, AI/ML have benefitted the fields of organic/inorganic catalyst design^10,11^, drug discovery^12,13^ and quantum dot synthesis^14,15^, in which simulation tools or high-throughput analytical platforms can supply many high-quality data points. In contrast, substantial obstacles exist in constructing a high-accuracy prediction model for biodegradable plastic substitutes, as the acquisition of high-quality data points is time-consuming and labour intensive^16,17^. Also, because every lab selects different natural components and follows individual protocols, data points from the literature are inconsistent, thus making AI/ML predictions unreliable^18,19^. Moreover, recent reports on all-natural plastic substitutes focused on optimizing a single characteristic (for example, optical transparency or mechanical strength), so the development of an AI/ML model that can predict multiple properties is still limited.

**Fig. 1:**
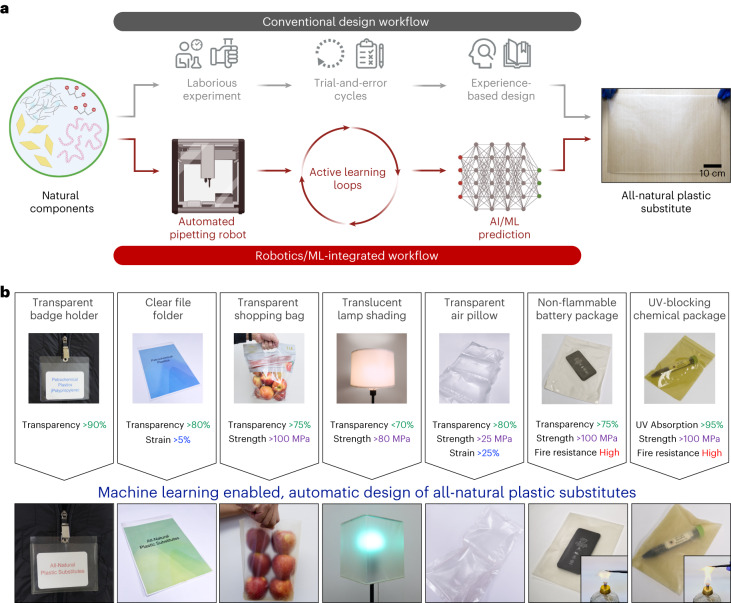
Machine intelligence-accelerated discovery of all-natural plastic substitutes with programmable properties. **a**, An integrated workflow using robotics and AI/ML predictions is demonstrated to construct a high-accuracy prediction model, enabling the accelerated discovery of all-natural plastic substitutes. Inset photo shows a model-suggested all-natural substitute that can be fabricated in a large area (with the dimensions of 53 cm × 38 cm). **b**, By inputting various property criteria for specific plastic products, the prediction model can automate the inverse design of all-natural substitutes to replace transparent badge holders, clear file folders, transparent shopping bags, translucent lamp shades, transparent air pillows, non-flammable battery packages and UV-blocking chemical packages. Top row photos show non-biodegradable plastic products and their property criteria. Bottom row photos show the biodegradable, all-natural substitutes suggested by the prediction model.

In this Article, an integrated workflow that uses robotics and AI/ML predictions was realized to accelerate the discovery of all-natural plastic substitutes with programmable optical, thermal and mechanical properties (Fig. 1a). Four generally-recognized-as-safe (GRAS) natural components, including cellulose nanofibres (CNFs), montmorillonite (MMT) nanosheets, gelatin and glycerol, were selected as the building blocks to fabricate various all-natural plastic substitutes (see Supplementary Note 1 and Supplementary Fig. 1 for the selection rationale). An automated pipetting robot (that is, OT-2 robot) was first commanded to prepare 286 nanocomposites with varying CNF/MMT/gelatin/glycerol ratios, and the film qualities were evaluated to train a support-vector machine (SVM) classifier. Next, through 14 active learning loops with data augmentation, 135 all-natural nanocomposites were stagewise fabricated, enabling the construction of an artificial neural network (ANN) model with high prediction accuracy across the entire design space. By harnessing the model’s predictive power, two-way design tasks were demonstrated, including (1) accurately predicting multiple characteristics of an all-natural nanocomposites from its composition and (2) automatically suggesting suitable biodegradable plastic alternatives with user-designated features. As shown in Fig. 1b, by inputting specific property criteria, the prediction model discovered suitable all-natural substitutes for diverse plastic replacements, without the need of iterative optimization experiments. Several data-scientific insights were generalized by SHapley Additive exPlanations (SHAP) model interpretation and validated by molecular dynamics (MD) simulations. Furthermore, through strategic selections of building blocks combined with a model expansion method, the prediction model continually expanded its design space and broadened the range of achievable functions. Our hybrid approach, involving robot-assisted experiments, data science and simulation tools, offers an unconventional design platform to accelerate the invention of eco-friendly, biodegradable plastic substitutes from the GRAS database.

## Composition-dependent physicochemical properties of the nanocomposite films

The characterizations of the four building blocks are provided in Supplementary Figs. 2 and 3. By casting the MMT/CNF/gelatin/glycerol mixtures followed by overnight evaporation, the all-natural nanocomposite films were obtained (scanning electron microscopy (SEM) images in Supplementary Fig. 4). As shown in Supplementary Fig. 5, by adjusting the MMT/CNF/gelatin/glycerol ratios, the physicochemical properties of nanocomposite films varied in a non-linear and hard-to-predict manner. Supplementary Note 2 estimates that >23,000 nanocomposite films are required to build an extensive database for four components (each with a step size of 2.0 wt.%). However, conducting a such large number of experiments is impractical due to finite resources and time constraints. Therefore, a robotics/ML-integrated workflow was implemented to discover suitable biodegradable nanocomposites for diverse plastic replacements.

## Design space definition

**Fig. 2:**
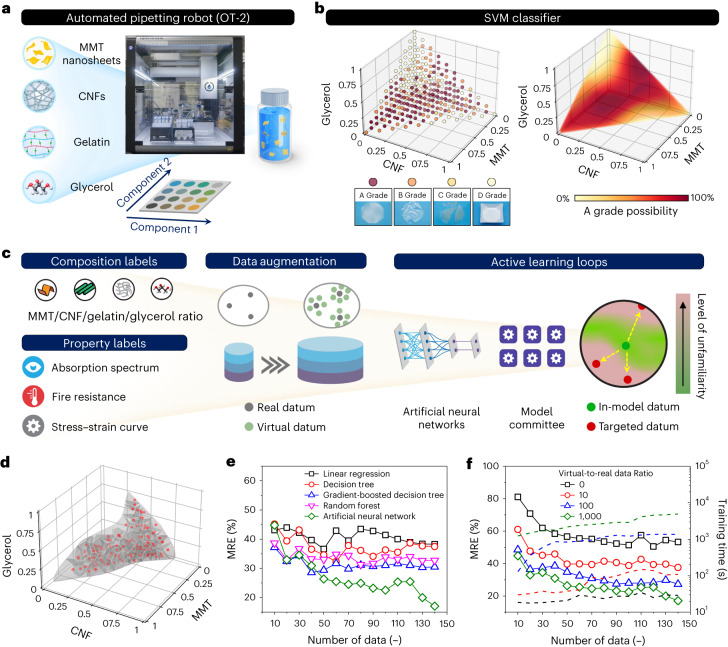
Construction of a high-accuracy prediction model via active learning loops, in silico data augmentation and robot–human teaming. **a**, An automated pipetting robot (that is, OT-2 robot) capable of preparing various MMT/CNF/gelatin/glycerol mixtures. **b**, Left: 286 discrete grades of nanocomposite films with varying MMT/CNF/gelatin/glycerol ratios. Inset: photos of nanocomposite films with four different grades. Right: a 3D heatmap representing the possibility of obtaining an A-grade nanocomposite film at a specific MMT/CNF/gelatin/glycerol ratio. **c**, Construction of an ANN-based prediction model *via* active learning loops, data augmentation and robot–human teaming. **d**, 3D diagram of Voronoi tessellation after 14 active learning loops. **e**, MRE values of different prediction models based on linear regression, decision tree, gradient-boosted decision tree, random forest and ANN algorithms. **f**, MRE values of different prediction models based on various virtual-to-real data ratios.

To construct a high-accuracy prediction model, we developed an AI/ML framework with three critical steps, including (1) boundary definition, (2) active learning and (3) in silico data augmentation. The first step was to define the boundaries of a feasible design space, during which an OT-2 robot was commanded to prepare a library of MMT/CNF/gelatin/glycerol mixtures with varying ratios (Fig. 2a). As demonstrated in Supplementary Movie 1, the OT-2 robot was able to prepare 286 mixtures within 6 h (four components, with a step size of 10 wt.%). Then, the robot-prepared solutions were cast onto planar polystyrene substrates and left to evaporate overnight. Afterward, based on the detachability and flatness of nanocomposite films (Methods), 286 samples were categorized into four cases (inset of Fig. 2b), ranging from (1) detachable and flat ones (A grade) to (2) detachable yet curved ones (B grade), (3) detachable yet fractured ones (C grade) and (4) non-detachable ones (D grade). As shown in Supplementary Tables 1 and 2, there were 132 A grades, 36 B grades, 46 C grades and 72 D grades; multiple blind tests were performed by several researchers. Supplementary Note 3 and Supplementary Fig. 6 discuss the influence of evaporation substrates on the grades of nanocomposite films.

The discrete grades were input to train a SVM classifier to locate the maximal-margin hyperplanes between the data points with different grades (see Supplementary Note 4 for detailed description)^20^. The trained SVM classifier suggested a specific MMT/CNF/gelatin/glycerol ratio that led to an A-grade nanocomposite film at a high successful rate >94% (examined by a set of testing data points, Supplementary Table 3). As shown in Fig. 2b, by predicting the A-grade possibilities across the design space, a three-dimensional (3D) heatmap was produced. By setting the possibility threshold of getting A-grade nanocomposites to be 75%, a 3D, irregular feasible design space was defined and held ~48% of the entire design space (Supplementary Fig. 7). Supplementary Note 5 describes the necessity of each AI/ML unit.

## Model construction

As illustrated in Fig. 2c, the active learning loops were initiated by commanding the OT-2 robot to prepare ten mixtures with random ratios. After overnight drying, ten nanocomposite films were obtained, and their MMT/CNF/gelatin/glycerol ratios were recorded as the ‘composition’ labels. Afterward, each nanocomposite film underwent optical, fire-resistant and mechanical characterizations.

First, the transmittance spectrum of each nanocomposite film was characterized, and the transmittance values at 365, 550 and 950 nm were extracted as the ‘spectral’ labels (${T}_{{{\mathrm{UV}}}}$, ${T}_{{{\mathrm{Vis}}}}$ and ${T}_{{{\mathrm{IR}}}}$), respectively. Second, the fire resistance of each nanocomposite film was examined through a modified ASTM D6413 fire test, and the residual ratio (${{\mathrm{RR}}}$, defined in equation (1)) was recorded as the ‘fire’ label,

1 $${\mathrm{RR}}=A^{\prime} /A,$$

where $A$ and ${A}^{{\prime} }$ are the sample dimensions before and after the fire test, respectively. Third, the stress–strain curve of each nanocomposite film was characterized by performing a tensile test. Several mathematic equations were tested to fit the stress–strain curves, and the cubic Bézier equation was finally selected due to the highest coefficient of determination (${R}^{2}$ = 0.995, Supplementary Fig. 8). Five cubic Bézier parameters, including ultimate tensile strength (${\sigma }_{{\mathrm{u}}}$), fracture strain (${\varepsilon }_{{\mathrm{f}}}$), Young’s modulus ($E$) and two shape parameters (${\rm{\alpha }}$ and $\beta$), were extracted as the ‘mechanical’ labels. In short, one nanocomposite film produced one data point containing four ‘composition’, three ‘spectral’, one ‘fire’ and five ‘mechanical’ labels. In the initial round, ten data points were collected.

To improve model’s learning efficiency and address overfitting, a data augmentation method, user input principle, was introduced to synthesize virtual data points (see Supplementary Figs. 9 and 10, and Supplementary Note 6 for detailed description). Both virtual and real data points were used as the training data for an ANN model through fivefold cross-validation^21^. Then, the ANN model evaluated the unfamiliarity level of targeted data points on the basis of a hybrid acquisition function (so-called A score in equation (2))^22^,

2 $${\mathrm{A}}\,{{\mathrm{score}}}=\hat{L}\times \hat{\sigma },$$

where $\hat{L}$ denotes the Euclidean distance between in-model and model-targeted ‘composition’ labels, and $\hat{\sigma }$ denotes the prediction variance of the ANN committee (see detailed discussion in Supplementary Note 7). For the next active learning loop, the data points with the highest A scores in the feasible design space were selected.

Afterward, the OT-2 robot was re-activated and followed the model-suggested ‘composition’ labels to prepare a new set of MMT/CNF/gelatin/glycerol mixtures. After cast drying, each nanocomposite film underwent similar spectral, fire and mechanical characterizations, and the user input principle method was again applied to synthesize virtual data points. With the updated dataset, the prediction model was re-trained and suggested another set of targeted data points with the highest A scores for the next loop. A total of 14 active learning loops were conducted, and 135 all-natural nanocomposite films were stagewise fabricated (Supplementary Table 4), resulting in ~140,000 real and virtual data points.

To visualize how data points were collected and distributed during active learning loops, 3D diagrams of Voronoi tessellation were adopted. As shown in Fig. 2d and Supplementary Figs. 11 and 12, the average cell volumes and their volume variances decreased as the loop number increased, implying that the AI/ML framework suggested the data points in different subregions without forming uninformative data clusters. Next, the accuracy of multi-property prediction was evaluated using a set of testing data points (that were never input to ANN, Supplementary Table 5). By inputting the ‘composition’ values of testing data points, the prediction model output the ‘optical’, ‘fire’ and ‘mechanical’ labels, which were compared with the actual values of testing data points. The deviation between model-predicted property labels and actual property values was quantified using a mean relative error (MRE, see Methods for details). A smaller MRE value indicates higher prediction accuracy and vice versa. As demonstrated in Fig. 2e, after 14 active learning loops, the MRE decreased to around 17%, which was close to some measurement variations (~12% in the ${T}_{{{\mathrm{UV}}}}$ label and ~15% in the ${\varepsilon }_{{\mathrm{f}}}$ label). Among other prediction models, the ANN model demonstrated the lowest MREs and the highest accuracy of multi-property prediction. Supplementary Figs. 13 and 14, Supplementary Table 6 and Supplementary Note 8 compare the active learning sampling with other sampling methods.

Figure 2f shows that the ANN model without data augmentation presented a high MRE of >55% after 14 active learning loops, mainly due to a small amount of training data points that caused model overfitting. In this work, the optimal virtual-and-real data ratio was determined to be 1,000, which maximized the learning efficiency while keeping a short loop time. Supplementary Note 9 provides the estimated time for all fabrication and characterization steps in one active learning loop. On average, completing one loop took approximately 2.5 days. When the virtual-and-real data ratio further increased to 5,000 and 10,000, the model training and optimization for one loop took over 4 and 7 days, respectively. Finally, the ANN model with the lowest MRE of 17% was selected as ‘the champion model’.

## Property prediction of the nanocomposites

**Fig. 3:**
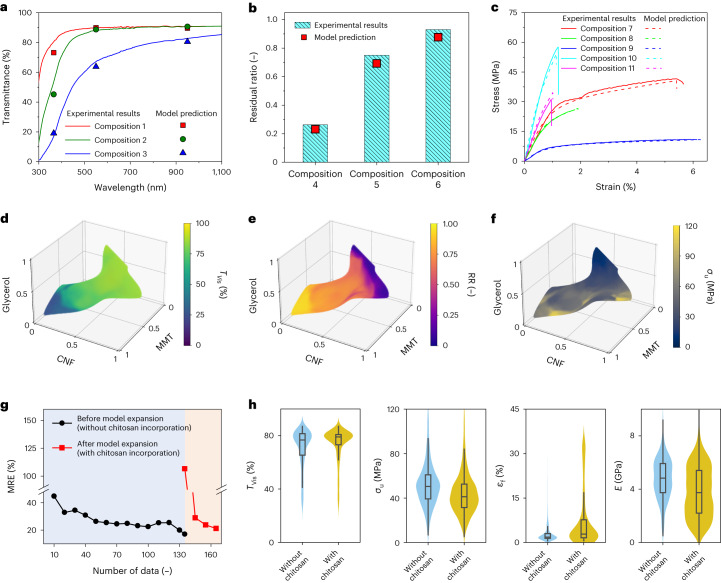
Accurate prediction of optical, flammable and mechanical properties via a champion model. **a**–**c**, Comparison between actual optical transmittance spectra and model-predicted spectral labels of three all-natural nanocomposites (compositions 1–3) (**a**), actual ${{\mathrm{RR}}}$ values and model-predicted fire labels of three all-natural nanocomposites (compositions 4–6) (**b**), and actual stress–strain curves and model-predicted stress–strain curves of five all-natural nanocomposites (compositions 7–11) (**c**). Supplementary Table 7 summarizes the composition labels of 11 all-natural nanocomposites tested and predicted. **d**–**f**, 3D heatmaps representing the spatial distributions of model-predicted ${T}_{{{\mathrm{Vis}}}}$ (**d**), ${{\mathrm{RR}}}$ (**e**) and ${\sigma }_{{\mathrm{u}}}$ (**f**) labels within the feasible design space. **g**, MRE values of the champion model during the model expansion process to incorporate chitosan into the design space. **h**, Violin plots of ${T}_{{{\mathrm{Vis}}}}$, ${\sigma }_{{\mathrm{u}}}$, ${\varepsilon }_{{\mathrm{f}}}$ and $E$ labels with and without chitosan incorporation. The embedded box plot within each violin plot indicates the 25th and 75th percentiles with the median represented by the centre line. Whiskers extend to 1.5× IQR from the box, *n* = 491,131.

As shown in Fig. 3a–c and Supplementary Table 7, the champion model accurately predicted the optical transmittances, fire resistances and stress–strain curves of multiple all-natural nanocomposites, which well matched the experimental results. By inputting all possible compositions within the feasible design space, the champion model produced a set of 3D heatmaps that visually represented the spatial distributions of all property labels, including thickness (Supplementary Fig. 15), ${T}_{{{\mathrm{Vis}}}}$ (Fig. 3d and Supplementary Fig. 17c), ${T}_{{{\mathrm{UV}}}}$ (Supplementary Figs. 16a and 17d), ${T}_{{{\mathrm{IR}}}}$ (Supplementary Figs. 16b and 17e), ${{\mathrm{RR}}}$ (Fig. 3e and Supplementary Fig. 17f), ${\sigma }_{{\mathrm{u}}}$ (Fig. 3f and Supplementary 17g), ${\varepsilon }_{{\mathrm{f}}}$ (Supplementary Figs. 16c and 17h) and $E$ (Supplementary Figs. 16d and 17i). Figure 3g and Supplementary Fig. 18 show that, through adjusting the MMT/CNF/gelatin/glycerol ratios, the optical, thermal and mechanical properties of all-natural nanocomposites were highly tunable across wide ranges.

With high prediction accuracy, the champion model was adopted to accelerate the discovery of high-strength structural materials using natural building blocks^23,24^. Through clustering analyses (see Supplementary Note 10 and Supplementary Table 8), the champion model suggested two suitable compositions: one with a high MMT loading (MMT/CNF/gelatin/glycerol = 64.2/6.7/23.8/5.3), and the other with a high CNF loading (3.7/61.8/28.4/6.1). By following two model-suggested compositions, we successfully fabricated the MMT-rich and CNF-rich nanocomposites, with their average ${\sigma }_{{\mathrm{u}}}$ values of 114 ± 18 and 98 ± 7 MPa (from 5 replicates), respectively (see Supplementary Fig. 19 for detailed description). To further strengthen the MMT-rich and CNF-rich nanocomposites, two-step treatments (including Ca^2+^ crosslinking and heat pressing) were conducted (Supplementary Figs. 20 and 21, and Supplementary Note 11), and the average *σ*_u_ values were improved to 468.6 ± 52.6 MPa (Supplementary Fig. 20a, with the highest *σ*_u_ of 520.7 MPa,) and 463.0 ± 35.7 MPa (Supplementary Fig. 20b, with the highest *σ*_u_ of 521.0 MPa).

## Model expansion method to incorporate new building blocks

To further enrich the portfolio of all-natural plastic substitutes, a model expansion method was applied to incorporate chitosan as the fifth building block, due to its excellent properties of antimicrobial activity and biocompatibility^25,26^. As depicted in Fig. 3g, the prediction model guided three additional active learning loops to integrate the new degree of freedom (that is, chitosan loading) into the champion model. Throughout the model expansion phase, 133 experiments were conducted: 90 to refine the SVM classifier (Supplementary Table 9) and 43 to retrain the champion model (Supplementary Table 10). This model expansion phase spanned ~13 days. As shown in Fig. 3g, the prediction model maintained high predictive accuracy after three loops, and the MREs decreased from 107% to 21%. As highlighted in Fig. 3h, the incorporation of chitosan notably elevated the ultimate strains of all-natural substitutes from 15% (without chitosan) to 34% (with chitosan). By utilizing the expanded model, we produced two additional all-natural plastic substitutes with high strains for clear file folders and transparent air pillows in Fig. 1b and Supplementary Table 11. Further discussion is detailed in Supplementary Note 12.

## Inverse design of all-natural substitutes for diverse plastic replacement

**Fig. 4:**
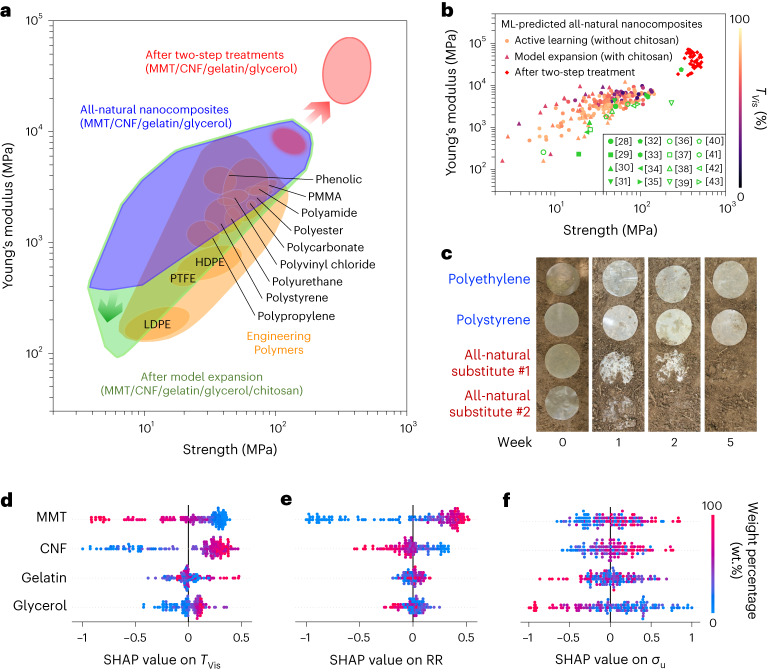
AI/ML-accelerated inverse design of all-natural nanocomposites for diverse plastic replacement with model interpretation. **a**, Ashby diagram (ultimate strength (${\sigma }_{{\mathrm{u}}}$) versus Young’s modulus ($E$)) for engineered polymers and model-predicted all-natural nanocomposites^27^. The champion model was able to suggest a library of all-natural nanocomposites with programmable ${\sigma }_{{\mathrm{u}}}$ and $E$ values, which well matched the mechanical properties of phenolic, poly(methyl methacrylate) (PMMA), polystyrene (PS), polyvinyl chloride (PVC), polycarbonate (PC), polyamide (PA), polyurethane and polypropylene (PP). **b**, ${\sigma }_{{\mathrm{u}}}\;{{\mathrm{versus}}}\;{E}$ plot of >200 all-natural nanocomposites fabricated during active learning loops, model expansion, and after two-step treatments, and the dot colour represents the ${T}_{{{\mathrm{Vis}}}}$ label of each nanocomposite. Compared to the state-of-the-art works using repetitive design of experiments to discover the biodegradable plastic substitutes (scattered in the Ashby diagram), our AI/ML prediction approach was able to discover a library of all-natural plastic substitutes across the entire subregion. **c**, Biodegradability tests of two commercial plastic films (polypropylene and PS) and two all-natural substitutes buried in soils for 5 weeks. The MMT/CNF/gelatin/glycerol ratio of all-natural substitute #1 was 60.0/3.0/24.0/13.0; the MMT/CNF/gelatin/glycerol ratio of all-natural substitute #2 was 6.0/36.0/1.0/57.0. **d**–**f**, Normalized SHAP values of MMT, CNF, gelatin and glycerol loadings on ${T}_{{{\mathrm{Vis}}}}$ (**d**), ${{\mathrm{RR}}}$ (**e**) and ${\sigma }_{{\mathrm{u}}}$ (**f**).

Figure 4a shows the Ashby diagram that displays ${\sigma }_{{\mathrm{u}}}$ and $E$ of various engineered polymers (including plastics) and our all-natural substitutes^27^. Using AI/ML predictions, a library of all-natural substitutes was developed to satisfy the mechanical design region within 1 < ${\sigma }_{{\mathrm{u}}}$ < 120 MPa and 0.5 < $E$ < 9.9 GPa. After the two-step treatments, these model-suggested substitutes were densified, and the design region was extended into the ranges of 278 < ${\sigma }_{{\mathrm{u}}}$ < 521 MPa and 17.5 < $E$ < 71.7 GPa. Compared with the reported works in the literature (Fig. 4b and Supplementary Table 12)^28–43^, our robotics/ML integrated approach discovered a set of >150 all-natural substitutes that covered the entire subregion(s) of the Ashby diagram, enabling a wide range of plastic replacement. In Fig. 4b and Supplementary Fig. 22, the dot colours represent the ${T}_{{{\mathrm{Vis}}}}$ and ${{\mathrm{RR}}}$ values of each all-natural substitute, respectively.

To demonstrate the power of multi-property prediction, the champion model was employed to automate the inverse design of all-natural plastic substitutes with programmable physicochemical characteristics. As shown in Fig. 1b and Supplementary Table 11, multiple plastic products were targeted to be replaced, and every model-recommended all-natural plastic substitute exhibited optical transparency, fire retardancy and mechanical resilience in line with the diverse design criteria. By following the model-suggested compositions, various all-natural substitutes were produced in large areas (with the dimensions of 53 cm × 38 cm, inset of Fig. 1a and Supplementary Fig. 23a,b). It is worth noting that these all-natural plastic alternatives demonstrated long shelf lives at least over 6 months (as shown in Supplementary Fig. 23c,d).

To examine the biodegradability of all-natural substitutes, two all-natural substitutes were buried in soil, along with polystyrene and polyethylene films as the controls (Fig. 4c). After 5 weeks, both all-natural substitutes were completely decomposed, while the petrochemical plastics remained intact. Supplementary Fig. 24 further demonstrates that all-natural plastic substitutes lost >60% of their original weights after 2 weeks. The good biocompatibility of all-natural plastic substitutes was validated through multiple cytotoxicity experiments on L929 cells (Supplementary Fig. 25 and Supplementary Note 13). As shown in Supplementary Fig. 26, the properties of all-natural plastic substitutes remained stable under direct sunlight after 8 days. Supplementary Fig. 27 and Supplementary Note 14 further discuss the construction of a SVM classifier to design the all-natural plastic substitutes with different water stability levels.

## Model interpretation and composition–property correlations

To uncover complex composition–property correlations and improve model’s interpretability, Spearman’s rank correlation coefficients (see Supplementary Fig. 28 and Supplementary Note 15 for details) and SHAP model interpretation were implemented on over 150 data collected during active learning loops. SHAP is a game theoretic approach to explain the output of any AI/ML model^44^. A positive SHAP value refers to a positive correlation, and vice versa (see detailed description in Supplementary Fig. 29 and Supplementary Note 16). Taking ${T}_{{{\mathrm{Vis}}}}$ as an example (Fig. 4d), the SHAP values of MMT and CNF loadings fluctuated from −0.9 to +0.4 and from −1.0 to +0.5, respectively, indicating that both components were equally influential yet had the opposite effects on ${T}_{{{\mathrm{Vis}}}}$. In contrast, the gelatin and glycerol loadings were less impactful on ${T}_{{{\mathrm{Vis}}}}$, as their SHAP values were distributed in narrower ranges. A similar SHAP analysis was conducted on ${{\mathrm{RR}}}$ (Fig. 4e), where the MMT loading had a strong, positive impact. As shown in Fig. 4f, the SHAP values of MMT and CNF loadings on ${\sigma }_{{\mathrm{u}}}$ were both distributed from −0.6 to +0.8, suggesting that these two components might have synergistic strengthening effects at molecular scale, which was investigated through MD simulations next. Additional SHAP analyses on ${T}_{{{\mathrm{UV}}}}$, ${T}_{{{\mathrm{IR}}}}$, ${\varepsilon }_{{\mathrm{f}}}$ and $E$ are shown in Supplementary Fig. 30, and Supplementary Table 13 summarizes the impactful component(s) on each property label.

## MD simulations for investigating strengthening mechanisms

**Fig. 5:**
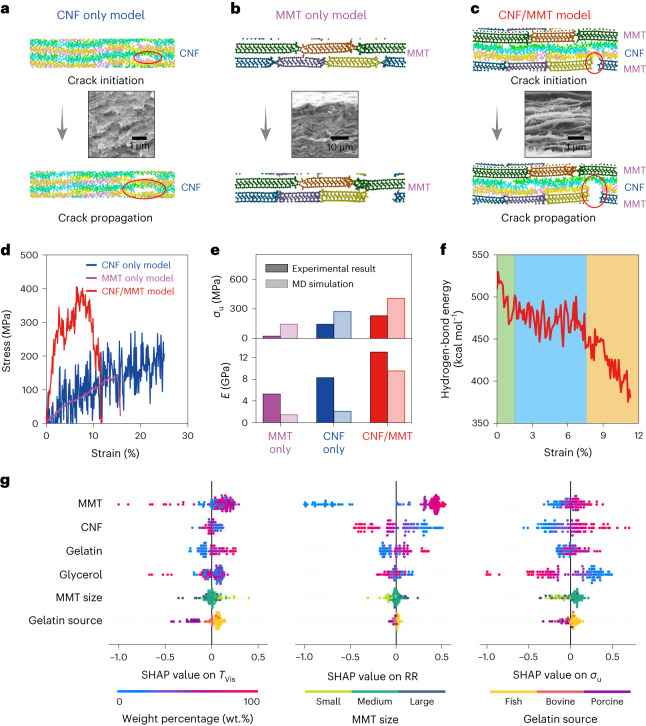
MD simulations reveal the deformation and failure mechanisms at the molecular scale. **a**–**c**, Atomic structures of CNF only (**a**), MMT only (**b**) and MMT/CNF (**c**) models before and after tensile failure. Insets are the SEM images of the fracture surfaces of CNF only, MMT only and MMT/CNF thin films. **d**, Simulated stress–strain curves of CNF only, MMT only and MMT/CNF models. **e**, Comparison of ultimate strengths and Young’s moduli extracted from MD-simulated and experimental results. **f**, Variation of hydrogen-bond energy in the MMT/CNF model as a function of tensile strain, showing three stages, namely initial decreasing stage (shaded in green), fluctuation stage (shaded in blue) and final decreasing stage (shaded in yellow). **g**, Normalized SHAP values of MMT loading, CNF loading, gelatin loading, glycerol loading, gelatin source and MMT size on *T*_Vis_, RR and *σ*_u_.

To investigate the strengthening mechanism between CNF chains and MMT nanosheets, we performed MD simulations on three models under tension: CNF only, MMT only, and MMT/CNF models (Methods). The atomic structures of these models are shown in Fig. 5a–c, and their tensile failure processes are recorded in Supplementary Movies 2–4, respectively. Supplementary Fig. 31 shows alternative presentations of these models using different colours to represent various atoms. As shown in Fig. 5a and Supplementary Movie 2, the CNF only model exhibited chain sliding behaviours, which led to crack formation/propagation and eventually caused tensile failure^45^. As shown in Fig. 5d, the stress–strain curve of the CNF only model featured a zigzag profile, corresponding to the cascade events of hydrogen-bond formation, breaking and reformation between neighbouring cellulose chains. On the other hand, the MMT only model was more brittle and developed inter-particle fractures upon tension, as shown in Fig. 5b and Supplementary Movie 3. As shown in Fig. 5d, the stress–strain curve of the MMT only model was quasi-linear and had an abrupt stress drop upon tensile failure.

In the MMT/CNF model, the tensile failure mechanism was distinct from the CNF only and MMT only models. As shown in Fig. 5c and Supplementary Movie 4, cracks initially developed between neighbouring MMT particles upon tension. Through these open cracks, the cellulose chains underwent localized tensile deformation, further propagating the cracks towards tensile failure of the MMT/CNF model. As the MMT/CNF interfaces exhibited high binding energy, the cracks upon tension propagated only in the perpendicular direction (in the *y* direction), and the MMT/CNF model involved a certain amount of CNF fractures upon tension, thus demonstrating a higher tensile strength (Fig. 5d).

To validate the MD simulation results, we fabricated three thin-film samples through vacuum filtration: one MMT only, one CNF only, and one from a 1:1 MMT/CNF mixture. As insets shown in Fig. 5a–c, the tensile fracture surfaces of these samples were observed under SEM. For example, the MMT only film displayed a sharp and clear-cut fracture surface, whereas the CNF only film showcased that fibres were pulled out at the points of rupture. In contrast, the MMT/CNF film revealed rough, rugged fracture surfaces, featuring multiple MMT/CNF subcomponents that had intertwined due to tension. Supplementary Fig. 32 shows the stress–strain curves of the MMT only, CNF only and MMT/CNF thin-film samples. As summarized in Fig. 5e, both MD simulations and experimental results show similar trends in ultimate strengths and Young’s moduli (MMT/CNF > CNF only > MMT only). Supplementary Note 17 further details the comparison between CNF only and MMT only models.

Figure 5f plots the variation of hydrogen-bond energy in the MMT/CNF model as a function of tensile strains. At the initial decreasing stage (shaded in green), the curvy cellulose chains are first straightened, and the intrachain hydrogen bonds (between repeating unit of individual cellulose chains) are gradually broken. As the fluctuation stage (shaded in blue), the elongation of cellulose chains is associated with many breaking and reforming events of interchain hydrogen bonds. At the final decreasing stage (shaded in yellow), an abrupt drop of hydrogen-bond energy corresponds to the breaking of many hydrogen bonds during the crack propagation process.

## Sensitivity analyses on structural and physical attributes of building blocks

To understand the influences of both structural and physical attributes of building blocks on the end-product properties, several sensitivity analyses and MD simulations were implemented in Supplementary Figs. S33–35 and Supplementary Note 18. As shown in Supplementary Fig. 36, 24 different MMT/CNF/gelatin/glycerol ratios were selected, and 6 sets of all-natural nanocomposites were prepared using three different gelatin sources (cold fish skin, porcine skin and bovine skin) and three different MMT sizes (large-, medium- and small-sized nanosheets). Subsequently, the optical, fire-resistant and mechanical properties of 144 all-natural nanocomposites were evaluated and fed into the prediction model. Next, SHAP analyses were used to determine the influences of different gelatin sources and MMT sizes on all nine property labels (Fig. 5g and Supplementary Fig. 37). The SHAP analyses suggested that both gelatin source and MMT size had considerable impacts on the optical properties (${T}_{{{\mathrm{IR}}}}$, ${T}_{{{\mathrm{Vis}}}}$ and ${T}_{{{\mathrm{UV}}}}$), while their influences on the fire-resistant and mechanical properties (${{\mathrm{RR}}}$, ${\sigma }_{{\mathrm{u}}}$, ${\varepsilon }_{{\mathrm{f}}}$ and $E$) were limited.

## Conclusions

In conclusion, an unconventional design platform that utilized automated robots, machine intelligence, wet-lab experiments and simulation tools was developed to discover a library of all-natural nanocomposites as biodegradable plastic substitutes with programmable optical, fire-resistant and mechanical properties. Furthermore, compared to the state-of-the-art works in Supplementary Table 14^46–48^, this ML/robotics-integrated workflow stimulates the development of various functional materials with multi-property optimization, which can be applied to a wide range of nanoscience fields, including tactile sensors^49,50^, stretchable conductors^51,52^, electrochemical electrolyte optimization^53,54^ and thermal insulative aerogels^55,56^.

Still, there exist several ongoing challenges and limitations associated with the AI/ML-integrated workflow for the accelerated design of all-natural plastic substitutes. First, no available collaborative robotics systems can automate the entire preparation and characterization processes for all-natural nanocomposites. Therefore, manual operations are still required to connect each stage for sample preparation and/or characterization. When more building blocks and structural/chemical features are included, the time and manpower needed for constructing an accurate AI/ML model will be inflated without robot-automated experimentation. Second, the quality of natural building blocks may vary from batch to batch. Therefore, stringent quality controls for each building block are crucial, especially for large-scale production and manufacturing. Third, data fusion with cost analyses and life cycle analyses into the champion model would be highly beneficial, allowing for identifying the optimal all-natural plastic substitutes that meet desired properties as well as provide the benefits of cost saving and environmental impact reduction. Last, the end-of-life processing of all-natural plastic substitutes has not been considered, which could be converted into biofuels or other valuable chemicals.

## Methods

### Materials

MMT (BYK Additives Incorporation; Cloisite Na+), northern bleached softwood kraft (NBSK) pulp (NIST RM 8495), TEMPO (Sigma-Aldrich, 99%), sodium bromide (NaBr, Sigma-Aldrich, ACS reagent, ≥99.0%), sodium hypochlorite solution (NaClO, Sigma-Aldrich, reagent grade, available chlorine 10–15%), sodium hydroxide (NaOH, Sigma-Aldrich, reagent grade, ≥98%), gelatin (Sigma-Aldrich, from cold-water fish skin) and glycerol (Sigma-Aldrich, ACS reagent, ≥99.5%) were used as received without further purification. Deionized (DI) water (18.2 MΩ) was obtained from a Milli-Q water purification system (Millipore) and used as the water source throughout this work.

### Preparation of MMT nanosheet dispersion

The MMT nanosheet dispersion was prepared according to the literature^57^. To obtain medium-sized MMT nanosheets, MMT powders were mixed in DI water at 10 mg ml^−1^, and the mixture was ultrasonicated for 2 h and continuously stirred for another 12 h. Afterward, the mixture was centrifuged at 1,252*g* for 60 min, and the supernatant was then collected as the dispersion of MMT nanosheets with the concentration about 8 mg ml^−1^. To obtain small-sized MMT nanosheets, the ultrasonication time was extended to 3 h, and the mixture was centrifuged at 5,009*g* for 60 min. Conversely, for large-sized MMT nanosheets, the ultrasonication time was reduced to 1 h, and the mixture was centrifuged at a slower speed of 489*g* for 15 min.

### Preparation of CNF dispersion

The CNF dispersion was prepared according to the literature^58^. First, 20 g of NBSK pulp was suspended in 1.0 litre of DI water, and then TEMPO (2 × 10^−3^ mol) and NaBr (0.02 mol) were added into the pulp. The TEMPO-mediated oxidation was initiated by adding 0.2 mol of NaClO, and the oxidation process was maintained under continuous stirring for 5–6 h, during which the pH was controlled at 10.0 by adding NaOH solution (3.0 M). The TEMPO-oxidized pulp was repeatedly washed with DI water until the pH returned back to 7.0. Afterward, the pulp was disassembled in a microfluidizer processor (Microfluidics M-110EH), and the concentration of CNF dispersion was about 10 mg ml^−1^.

### Preparation of gelatin solution

A total of 8.0 g of gelatin was dissolved in 1.0 litre of DI water followed by continuous stirring for 48 h, and the concentration of gelatin solution was 8.0 mg ml^−1^.

### Preparation of glycerol solution

A total of 8.4 g of glycerol was dissolved in 1.0 litre of DI water followed by continuous stirring for 12 h, and the concentration of glycerol solution was 8.4 mg ml^−1^.

### Fabrication of all-natural nanocomposite films via an automated pipetting robot

An automated pipetting robot (Opentrons OT-2) was operated to prepare different mixtures with varying MMT/CNF/gelatin/glycerol ratios. For each mixture, the dispersions/solutions of MMT nanosheets, CNFs, gelatin and glycerol were mixed at different volumes. Afterward, the robot-prepared mixtures were vortexed at 3,000 rpm for 30 s and placed in a vacuum desiccator to remove air bubbles. Then, the mixtures were cast into a flat, polystyrene-based container at 40 °C and air dried for 48 h.

### Identification of A-grade nanocomposites

Each nanocomposite film was subject to detachment and flatness testing after it dried. Regarding detachability, except for samples that can be clearly labelled as detachable or non-detachable (Supplementary Fig. 38a), the mechanical delamination tests were conducted to measure the binding energies of nanocomposite films on hydrophobic polystyrene substrates. As shown in Supplementary Fig. 39. all the detachable samples exhibited the binding energies of <0.4 J cm^−2^, while the undetachable ones were with the binding energies >0.6 J cm^−2^. Thus, the threshold binding energy was set to be 0.5 J cm^−2^ to classify the detachability of nanocomposite films. Regarding flatness, except for samples that can be clearly labelled as flat or curved (Supplementary Fig. 38b), a high-speed laser scanning confocal microscope was employed to characterize the roughness of nanocomposite films. As demonstrated in Supplementary Fig. 40a, the nanocomposite films considered ‘flat’ exhibited height differences of <200 µm. Meanwhile, those considered ‘curved’ typically showcased height differences of >500 µm (Supplementary Fig. 40b). Once the detachment and flatness tests were finished, only the detachable and flat samples were identified as A-grade nanocomposites.

### Determination of SVM classifier accuracy

After constructing the SVM classifier, we examined its prediction accuracy using a set of testing data points. As shown in Supplementary Table 3, a total of 35 MMT/CNF/gelatin/glycerol ratios were randomly selected, and 35 nanocomposite films were fabricated according to the established procedure. Detachment and flatness tests were conducted to categorize these nanocomposite films into different grades. Subsequently, the MMT/CNF/gelatin/glycerol ratios (that is, composition labels) were input into the SVM classifier to obtain the predicted grades, which were then compared with the experimental results. In this study, the SVM classifier accurately predicted the grades for 33 out of the 35 nanocomposite films, resulting in a prediction accuracy of 94.3%.

### Determination of ANN-based prediction model accuracy

After constructing the ANN-based prediction model, we examined its prediction accuracy using a set of testing data points.

The deviation between model-predicted property labels and actual property values was quantified using a MRE, defined in equation (3),

3 $${\rm{MRE}}=\frac{1}{N}\,\mathop{\sum }\limits_{i=1}^{N}\left|\frac{{{{\mathrm{output}}}}^{i}-{E}^{i}}{{E}^{i}}\right|,$$

where *N* is the cumulative number of testing data, ${{{\mathrm{output}}}}^{i}$ is the model-predicted property labels based on a testing datum (*i*), ${E}^{i}$ is the actual property values of a testing datum (*i*). A smaller MRE value indicates higher prediction accuracy and vice versa.

### Film thickness characterization

The thickness of each all-natural nanocomposite was initially determined using a digital micrometre (293-340-30, Mitutoyo). For each strip sample used in the mechanical test, the nanocomposite thickness was gauged at three separate points, and the average thickness value was derived. Furthermore, the thickness of the all-natural nanocomposites was verified using a field emission scanning electron microscope (Tecan XEIA) operating at 15.0 kV. Cross-sectional SEM images were taken, followed by thickness measurements to validate the earlier readings.

### Transmittance spectrum characterization

The transmittance spectra of all-natural nanocomposites were measured with an ultraviolet (UV)–visible spectrometer from 250 to 1,100 nm (UV-3600 Plus, PerkinElmer) equipped with an integrating sphere. The transmittance values at 365, 550 and 950 nm were extracted as the ‘spectral’ labels (${T}_{{{\mathrm{UV}}}}$, ${T}_{{{\mathrm{Vis}}}}$ and ${T}_{{{\mathrm{IR}}}}$), respectively.

### Fire resistance characterization

The fire resistances of the all-natural nanocomposites were assessed using a horizontal combustibility testing method, modified from the standard test method (ASTM D6413)^59^. The all-natural nanocomposites were cut into 1 cm × 1 cm squares, and then they were exposed to the flame of an ethanol burner for 30 s (with a flame temperature ranging from 600 °C o 850 °C)^60^. The fire resistance of the all-natural nanocomposites was quantified in terms of ${{\mathrm{RR}}}$. Three replicates were conducted, and the average ${{\mathrm{RR}}}$ values were recorded as the fire labels.

### Mechanical property characterization

The stress–strain curves of the all-natural nanocomposites were determined using a mechanical testing machine (Instron 68SC-05) fitted with a 500-N load cell. After calibrating the load cell, the all-natural nanocomposites were cut into 3 cm × 1 cm stripes and subject to a tensile test at an extension rate of 0.02 mm s^−1^. The tensile tests started with an initial fixture gap of 2 cm. Three replicates were conducted for each all-natural nanocomposites.

### Materials characterization

The surface functional groups of all-natural nanocomposites were characterized using a Fourier transform infrared spectroscopy (FT-IR, Thermo Nicolet NEXUS 670).

### Biocompatibility tests of all-natural nanocomposites

The cytotoxic effects of all-natural nanocomposites on the cultured cells (that is, L929 cells) were determined by complying with ISO 10993. Six all-natural nanocomposites with different MMT/CNF/gelatin/glycerol ratios were incubated with Dulbecco’s modified Eagle medium (DMEM, Gibco) supplemented with foetal bovine serum (Biological Industries) at 37 °C for 24 h, and the media were then extracted for cell culture. L929 cells were then seeded in 96-well cell culture plates at the density of 1 × 10^4^ cells per well and incubated in a standard cell incubation environment with 5% CO_2_. After 24 h of cell culture, the culture media were removed and replaced with the extracts of all-natural nanocomposites followed by additional 24-h incubation. After 24 h, the culture media were withdrawn, and 3-(4,5-dimethylthiazol-2-yl)-2,5-diphenyltetrazolium bromide solution was added to each well. Then, the cell culture plate was incubated for 2 h at 37 °C. After the 3-(4,5-dimethylthiazol-2-yl)-2,5-diphenyltetrazolium bromide solution was discarded, 200 ml of dimethyl sulfoxide was added to dissolve the formazan crystals. The optical density of the formazan solution was read by an enzyme-linked immunosorbent assay plate reader at 570 nm with a reference wavelength of 650 nm.

The cytotoxicity of all-natural nanocomposites was evaluated by a cytotoxicity detection kit (Roche). First, the L929 cells were incubated with the all-natural nanocomposite extracts at 37 °C for 24 h, and the medium (100 µl) was collected and incubated with the reaction mixture from the kit following the manufacturer’s instructions. LDH content was assessed by enzyme-linked immunosorbent assay and read at an absorbance of 490 nm in a plate reader with a reference wavelength of 630 nm. To further confirm the cytotoxicity of all-natural nanocomposites, a fluorescence-based live/dead assay (LIVE/DEAD kit, Life) was performed. After the L929 cells were cultured with the extracts for 24 h, calcein was mixed with ethidium homodimer-1 according to the manufacturer’s instructions, and the dye (100 µl) was mixed with the retained medium (100 µl), which was added to each well and incubated at 37 °C for 15 min. After the incubation, we used an inverted microscope (Leica DMi8) to capture the images of live (green) and dead (red) cells. Fluorescence with excitation wavelengths of 488 nm and 561 nm was used to visualize the green (515 nm) and red (635 nm) fluorescence signals emitted by calcein and ethidium homodimer-1, respectively. ImageJ software was employed to calculate the proportion of live and dead cell areas. The relative percentages of fluorescence intensity were also determined. ImageJ was utilized to quantify the areas of red and green fluorescence, which produced average values. These numerical values were subsequently used in the quantification formula to determine the fluorescence intensity of live/dead cells in equation (4):

4 $${\rm{Fluorescence}}\,{\rm{intensity}}=({\rm{Live}}/{\rm{Dead}})/({\rm{Live}}+{\rm{Dead}})\times 100 \%$$

### MD simulations

The full atomistic simulations utilized the ReaxFF potential within the Large-scale Atomic/Molecular Massively Parallel Simulator (LAMMPS) simulation package^61^. The ReaxFF potential is widely used to describe chemical bonds and weak interactions of cellulose chains and MMT nanosheets^62,63^. As shown in Supplementary Fig. 41a, the MD model of the MMT/CNF nanocomposite configured as a multilayered microstructure comprising alternating CNF chains and MMT nanosheets, similar to the SEM observations in Supplementary Fig. 41b. The length of the cellulose chains was set to 104 Å, and the scale of the MMT nanosheets was randomly set between 30 Å and 60 Å, corresponding to the length scale ratio in the experiments (*L*_CNF_:*L*_MMT_ = 1:2). The cellulose chains and MMT nanosheets were passivated by polar hydrogens or –OH groups. The entire system was equilibrated under the isothermal-isobaric ensemble (that is, NPT ensemble) at 300 K and 0 atm, using the Nosé–Hoover thermostat and barostat. Then, the micro-canonical ensemble was applied in the stretching process. The timestep was set as 0.5 fs, and the periodic boundary conditions were applied in all directions (*x*, *y* and *z*) for all models. To better understand intermolecular interactions, both cellulose chains and MMT nanosheets were randomly arranged in alignment in the periodical box. All calculations were relaxed using the conjugate gradient algorithm to minimize the total energy of the system until the total atomic forces were converged to less than 10^–9^ eV Å^–1^.

## Online content

Any methods, additional references, Nature Portfolio reporting summaries, source data, extended data, supplementary information, acknowledgements, peer review information; details of author contributions and competing interests; and statements of data and code availability are available at 10.1038/s41565-024-01635-z.

### Supplementary information

Supplementary Information Supplementary Figs. 1–41, Notes 1–18 and Tables 1–14.

Supplementary Video 1 Automated pipetting robot (that is, OT-2 robot) for preparing various MMT/CNF/gelatin/glycerol mixtures.

Supplementary Video 2 Deformation and tensile failure processes of the CNF only model.

Supplementary Video 3 Deformation and tensile failure processes of the MMT only model.

Supplementary Video 4 Deformation and tensile failure processes of the CNF/MMT model.

## Data Availability

The data that used for model training are available from the Zenodo repository Data for: Machine Intelligence-Accelerated Discovery of All-Natural Plastic Substitutes, accessible via 10.5281/zenodo.7916360. The data that support the plots within this paper and other findings of this study are available from the corresponding authors upon reasonable request.

## Code availability

The Python code to implement the ML tasks within this study are available from GitHub (https://github.com/chentl/MatAL).
