# Supplementary material for: Machine intelligence-accelerated discovery of all-natural plastic substitutes
Source: Nat Nanotechnol. 2024 Mar 18;19(6):782–91. doi: 10.1038/s41565-024-01635-z (PMC11186784; doi:10.1038/s41565-024-01635-z)
Supplement: Supplementary file 1 — Supplementary Figs. 1–41, Notes 1–18 and Tables 1–14. [file 41565_2024_1635_MOESM1_ESM.pdf]

nature nanotechnology

Article

https://doi.org/10.1038/s41565-024-01635-z

Machine intelligence-accelerated discovery of all-natural plastic substitutes

In the format provided by the authors and unedited

**Fig. S1**	Stress–strain curves of all-natural nanocomposites with 1–4 building blocks.	4
**Fig. S2**	TEM image of CNFs and MMT nanosheets.	5
**Fig. S3**	Stability of CNF, MMT, CNF/MMT dispersions.	6
**Fig. S4**	SEM images of all-natural nanocomposites.	7
**Fig. S5**	Composition-dependent physicochemical properties of all-natural nanocomposite films.	8
**Fig. S6**	Influence of evaporation substrates on the grades of nanocomposite films.	10
**Fig. S7**	Determination of a feasible design space.	11
**Fig. S8**	Using various mathematical equations to fit stress–strain curves.	12
**Fig. S9**	Similar physicochemical properties of all-natural nanocomposites upon small composition changes.	13
**Fig. S10**	Measurement variations of 9 property labels across multiple all-natural nanocomposite replicates with the same MMT/CNF/gelatin/glycerol ratios.	14
**Fig. S11**	3D diagrams of Voronoi tessellation at different active learning stages.	15
**Fig. S12**	Normalized average cell volumes of 3D Voronoi diagrams and their variances at different active learning stages.	16
**Fig. S13**	Distribution profiles of data points collected from different sampling methods	17
**Fig. S14**	Prediction model performance based on different sampling methods.	18
**Fig. S15**	Spatial distribution of model-predicted thickness labels.	19
**Fig. S16**	3D heatmaps representing the spatial distributions of model-predicted property labels.	20
**Fig. S17**	3D heatmaps with the gelatin loading shown on the z-axes.	21
**Fig. S18**	Violin plots of T_{UV}, T_{IR}, and RR labels.	22
**Fig. S19**	Clustering analyses for discovering high-strength, all-natural nanocomposites.	23
**Fig. S20**	Stress–Strain curves, SEM images and FTIR spectra of all-natural plastic nanocomposites before and after two-step treatments.	24
**Fig. S21**	Fire resistance and optical transmittance of densified all-natural nanocomposites.	25
**Fig. S22**	σ_u–E plot of >150 all-natural nanocomposites with their RR values.	26
**Fig. S23**	Large-area production of all-natural substitutes with long shelf lives.	27
**Fig. S24**	Biodegradability of all-natural plastic substitutes.	28
**Fig. S25**	Biocompatibility of all-natural plastic substitutes.	29
**Fig. S26**	Stability of all-natural plastic substitutes under sunlight.	30
**Fig. S27**	Water stability levels of various all-natural nanocomposites.	31
**Fig. S28**	Statistical analyses for all-natural nanocomposites between natural components and physicochemical properties.	32

**Fig. S29**	Working mechanism of SHAP.	33
**Fig. S30**	SHAP values of CNF, MMT, gelatin, and glycerol loadings on various property labels.	34
**Fig. S31**	Alternative presentations of CNF only, MMT only, and MMT/CNF models.	35
**Fig. S32**	Stress–strain curves of MMT only, CNF only, and MMT/CNF thin-film samples.	36
**Fig. S33**	Properties of all-natural nanocomposites with different gelatin sources and MMT sizes.	37
**Fig. S34**	Characterizations of MMT at different sizes.	38
**Fig. S35**	MD simulations of CNF/MMT models with shorter CNF lengths and smaller MMT sizes.	39
**Fig. S36**	24 MMT/CNF/gelatin/glycerol ratios selected for sensitivity analyses	40
**Fig. S37**	Normalized SHAP values of MMT loading, CNF loading, gelatin loading, glycerol loading, gelatin source, and MMT size on T_{UV}, T_{IR}, $\boldsymbol{\varepsilon_f}$, and E.	41
**Fig. S38**	Examples of all-natural nanocomposites with clearly classified detachability and flatness.	42
**Fig. S39**	Mechanical delamination tests.	43
**Fig. S40**	Flatness classification of nanocomposite films.	44
**Fig. S41**	Construction of a MMT/CNF model.	45

**Note S1**	Selection of natural components for all-natural plastic substitutes.	46
**Note S2**	Estimated number of experiments required to build an extensive dataset for all-natural nanocomposites.	47
**Note S3**	The influence of evaporation substrates on the grades of nanocomposite films	48
**Note S4**	Training of a support-vector machine (SVM) classifier.	49
**Note S5**	Necessity of multi-stage ML framework.	50
**Note S6**	User Input Principle (UIP) method.	51
**Note S7**	Calculation of *A Score* acquisition function.	52
**Note S8**	Comparative studies of active learning sampling method.	54
**Note S9**	Estimated time for the fabrication and characterization processes for all-natural nanocomposites.	55
**Note S10**	Working mechanism of clustering analysis.	57
**Note S11**	Two-step treatments of all-natural nanocomposites.	58
**Note S12**	Perpectives on model expansion method.	59
**Note S13**	Biocompatibility of all-natural nanocomposites.	59
**Note S14**	Water stability levels of various all-natural nanocomposites.	60

**Note S15**	Spearman's rank correlation coefficients.	61
**Note S16**	Shapley Additive exPlanations (SHAP).	62
**Note S17**	Comparison of Young's moduli of CNF only and MMT only models.	65
**Note S18**	Potential approaches to evaluating the structural and physical attributes of building blocks.	67

**Movie S1**	Automated pipetting robot (i.e., OT-2 robot) for preparing various MMT/CNF/gelatin/glycerol mixtures.	69
**Movie S2**	Deformation and tensile failure processes of the CNF only model.	69
**Movie S3**	Deformation and tensile failure processes of the MMT only model.	69
**Movie S4**	Deformation and tensile failure processes of the CNF/MMT model.	69

**Table S1**	Grades of 286 nanocomposites with different MMT/CNF/gelatin/glycerol ratios.	70
**Table S2**	Photos of 286 nanocomposites with different MMT/CNF/gelatin/glycerol ratios.	75
**Table S3**	Testing data for the SVM classifier.	87
**Table S4**	Training data for the prediction model.	88
**Table S5**	Testing data for the prediction model.	94
**Table S6**	Data for sampling method comparison	95
**Table S7**	Composition labels of 11 model-predicted all-natural substitutes.	103
**Table S8**	Comparison of model-suggested MMT-rich and CNF-rich nanocomposites with high σ_u.	104
**Table S9**	Grades of 90 nanocomposites with different MMT/CNF/chitosan/gelatin/glycerol ratios during the model expansion process.	105
**Table S10**	Dataset for the prediction model during the model expansion process.	107
**Table S11**	Summary of inverse design requests for targeted plastic products and model-suggested compositions.	109
**Table S12**	Comparison of our all-natural plastic substitutes with the state-of-the-art works.	110
**Table S13**	Summary of data-driven influential components by different data analysis methods.	112
**Table S14**	Comparison of our AI/ML framework with the literature.	113

**Supporting References**	114

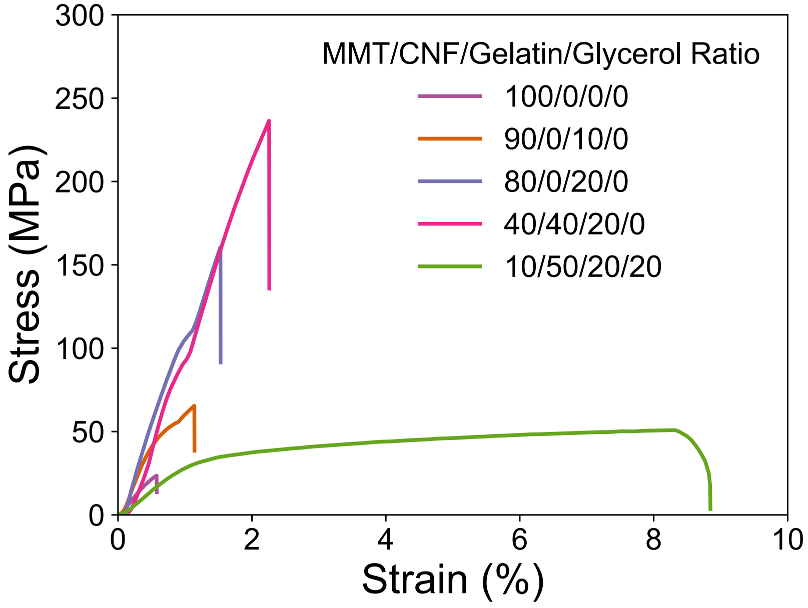

Fig. S1. Stress–strain curves of all-natural nanocomposites with 1–4 building blocks.

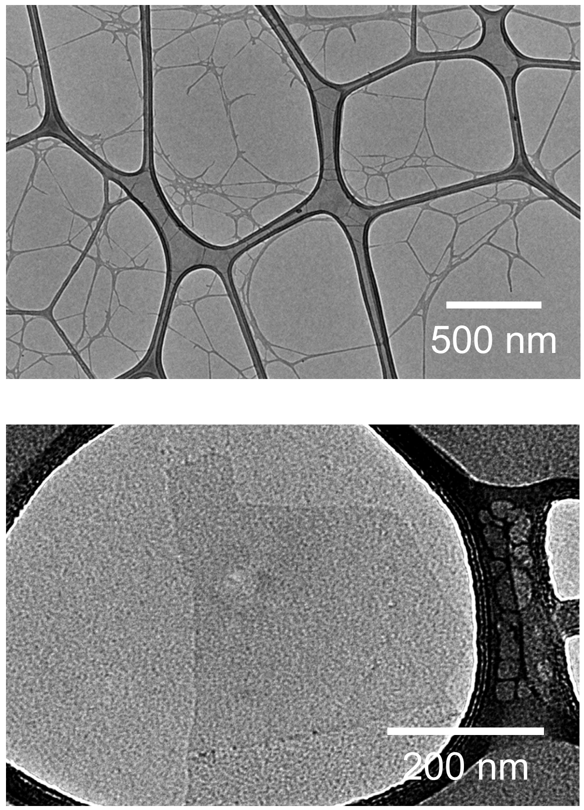

Fig. S2. TEM image of CNFs and MMT nanosheets. (**a**) TEM image of CNFs. The average diameter and length of CNFs are 10 nm and 1–2 μm, respectively. (**b**) TEM image of MMT nanosheets. The average dimensions of MMT nanosheets are 300 × 300 nm.

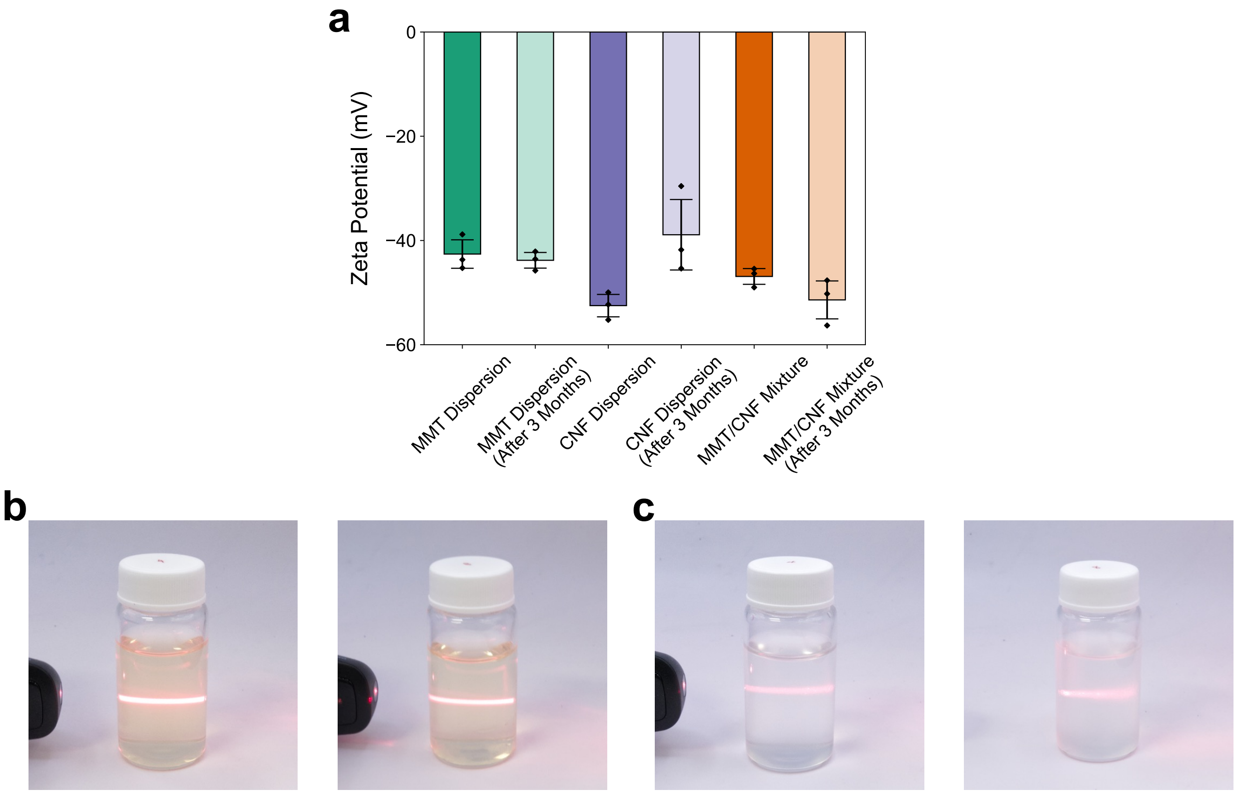

Fig. S3. Stability of CNF, MMT, CNF/MMT dispersions. (**a**) Zeta potentials of MMT, CNF, MMT/CNF dispersions before and after 3-month storage. Data are presented as mean ± s.d., n = 3, with each independent experiment marked by a black diamond. (**b**) Photos of MMT dispersion before and after 3-month storage. (**c**) Photos of CNF dispersion before and after 3-month storage.

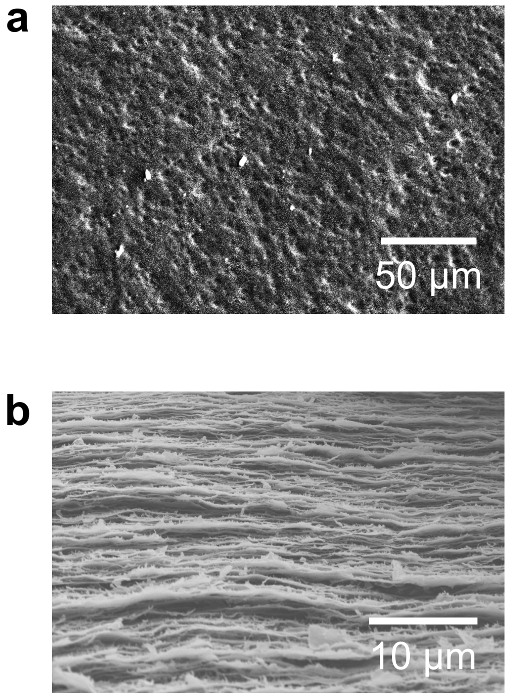

Fig. S4. SEM images of all-natural nanocomposites. (**a**) Top-down and (**b**) cross-sectional SEM images of an all-natural nanocomposites.

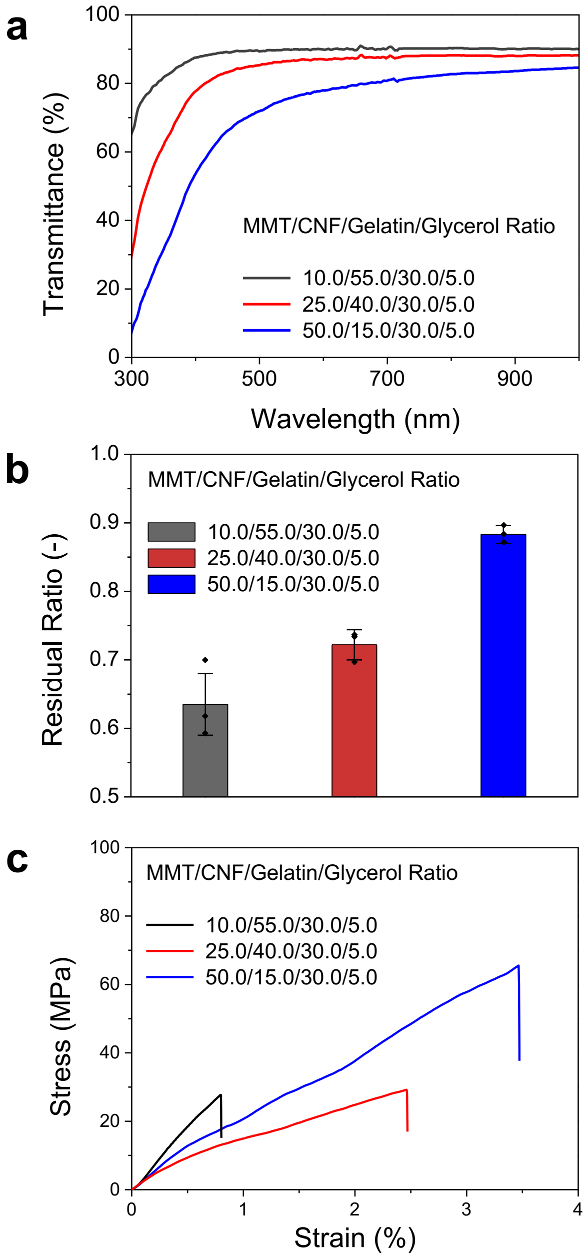

Fig. S5. Composition-dependent physicochemical properties of all-natural nanocomposite films. The MMT/CNF/gelatin/glycerol ratios affected the optical, fire-resistant, and mechanical properties of all-natural nanocomposites in a complex and non-linear mannar. (**a**) Transmittance spectra of three all-natural nanocomposites with different MMT/CNF/gelatin/glycerol ratios (10.0/55.0/30.0/5.0; 25.0/40.0/30.0/5.0; 50.0/15.0/30.0/5.0). (**b**) Residual ratios (RR) of three all-

natural nanocomposites with different MMT/CNF/gelatin/glycerol ratios (10.0/55.0/30.0/5.0; 25.0/40.0/30.0/5.0; 50.0/15.0/30.0/5.0). Data are presented as mean ± s.d., n = 3, with each independent experiment marked by a black diamond. (**c**) Stress–strain curves of three all-natural nanocomposites with different MMT/CNF/gelatin/glycerol ratios (10.0/55.0/30.0/5.0; 25.0/40.0/30.0/5.0; 50.0/15.0/30.0/5.0).

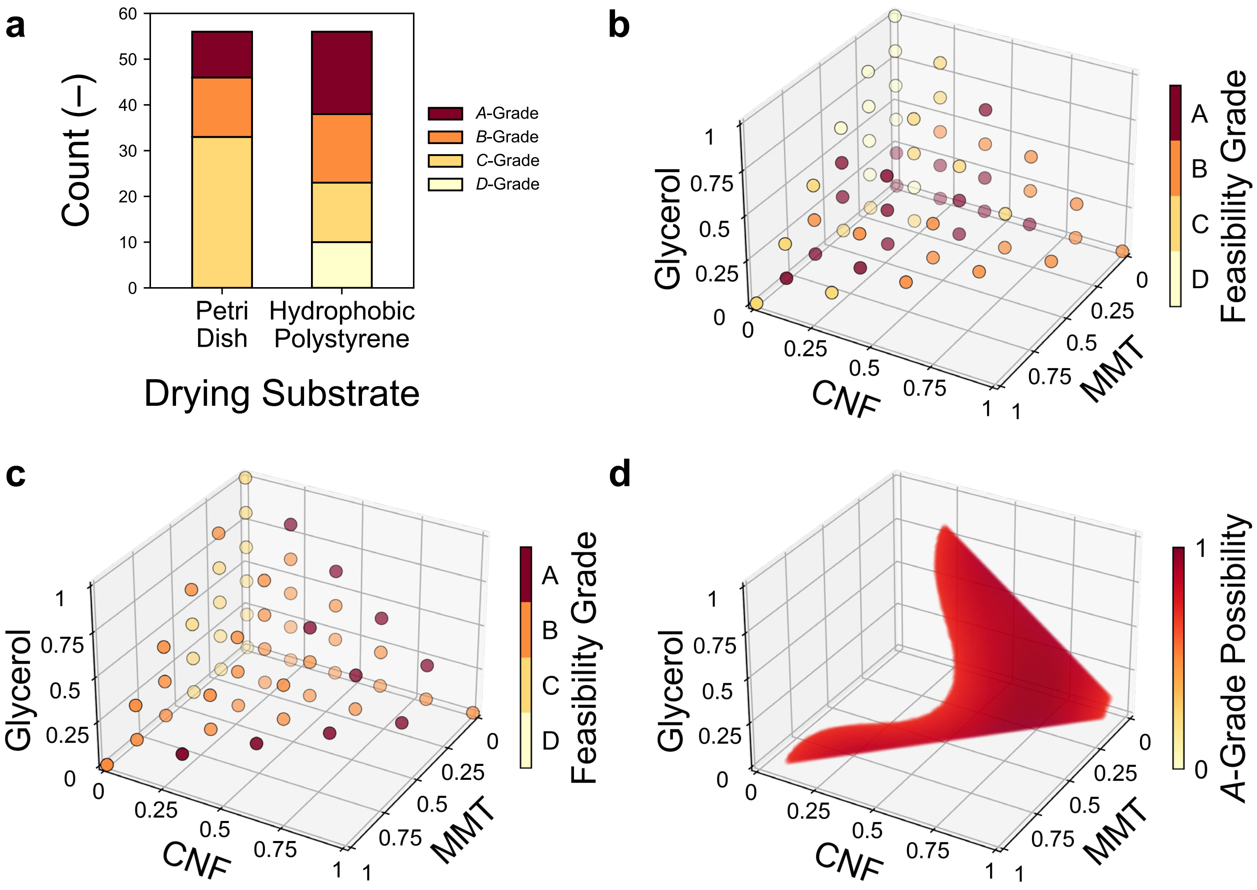

Fig. S6. Influence of evaporation substrates on the grades of nanocomposite films. (**a**) Numbers of nanocomposite films at different grades, obtained by drying on polycarbonate petri dishes or hydrophobic polystyrene substrates. (**b)** 56 discrete grades of nanocomposite films with varying MMT/CNF/gelatin/glycerol ratios, obtained by drying on hydrophobic polystyrene substrates. (**c**) 56 discrete grades of nanocomposite films with varying MMT/CNF/gelatin/glycerol ratios, obtained by drying on hydrophobic polystyrene substrates on polycarbonate petri dishes. (**d**) Feasible design space upon the use of polycarbonate petri dishes.

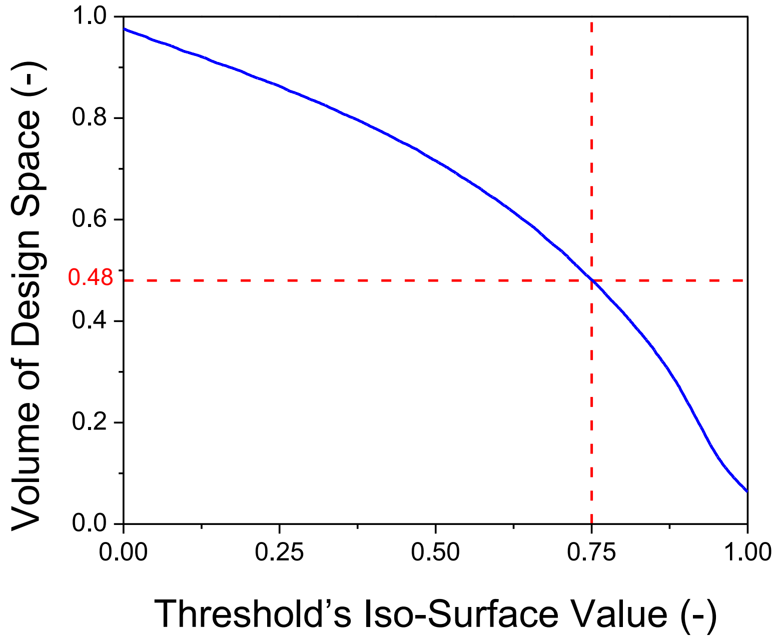

Fig. S7. Determination of a feasible design space. By selecting the iso-surfaces with different *A*-grade possibilities, the volume of a feasible design space changed accordingly. By setting the iso-surface with 75% *A*-grade possibility as the threshold, a feasible design space was defined and held ~48% of the entire design space.

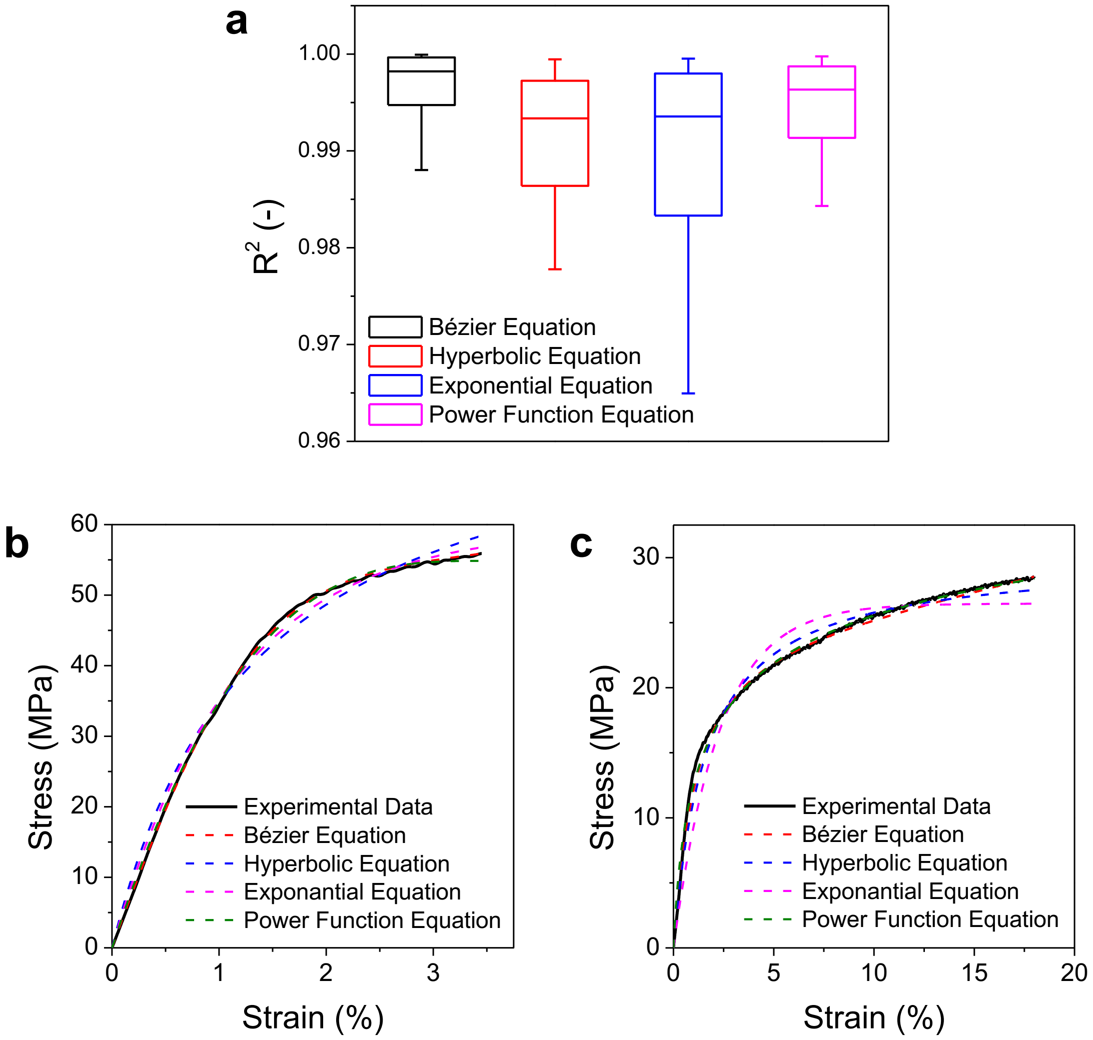

Fig. S8. Using various mathematical equations to fit stress–strain curves. Four classical mathematical equations, including cubic Bézier equation, hyperbolic equation, exponential equation, and power function equation, were used to fit the stress–strain curves of various all-natural nanocomposites. (**a**) Coefficients of determination (R^2) of four fitting equations. The box plot shows the 25th to 75th percentiles with the median at the center line, and whiskers extending to the 5th and 95th percentiles (n = 443). The cubic Bézier equation exhibited the highest R^2 of 0.995 and was adopted to fit the stress–strain curves of all-natural nanocomposites. (**b**) (**c**) Using four mathematical equations to fit the stress–strain curves of two all-natural nanocomposites.

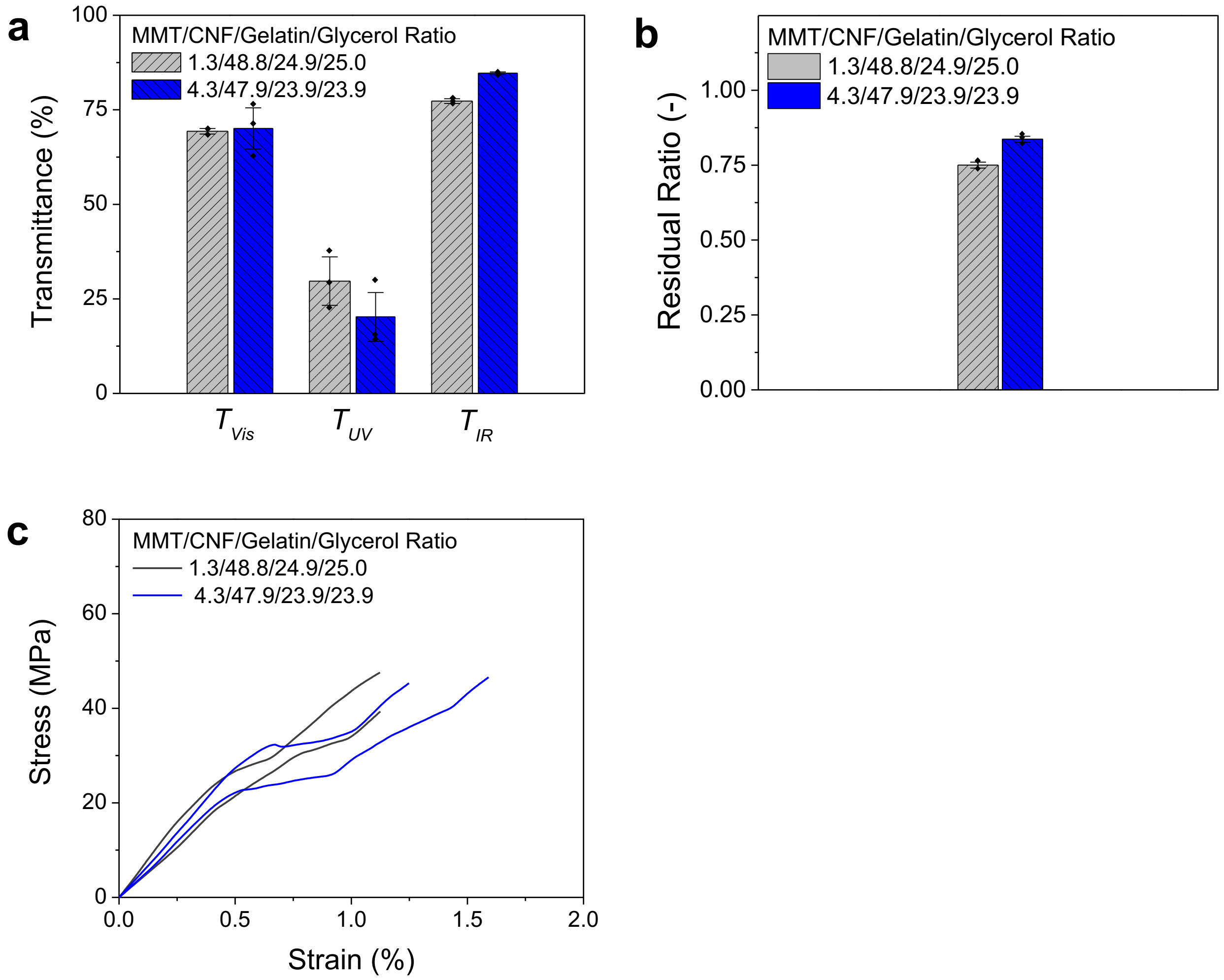

Fig. S9. Similar physicochemical properties of all-natural nanocomposites upon small composition changes. With similar MMT/CNF/gelatin/glycerol ratios of 1.3/48.8/24.9/25.0 and 4.3/47.9/23.9/23.9, two all-natural nanocomposites demonstrated similar (**a**) optical labels, (**b**) fire labels, and (**c**) mechanical labels (i.e., stress–strain curves). In each bar plots, data are presented as mean ± s.d., *n* = 3, with each independent experiment marked by a black diamond.

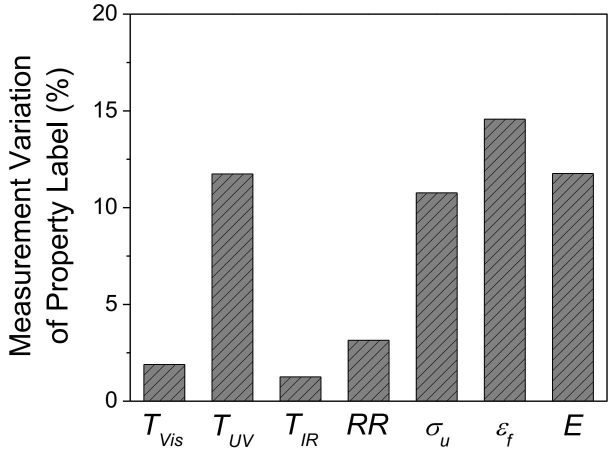

Fig. S10. Measurement variations of 9 property labels across multiple all-natural nanocomposite replicates with the same MMT/CNF/gelatin/glycerol ratios.

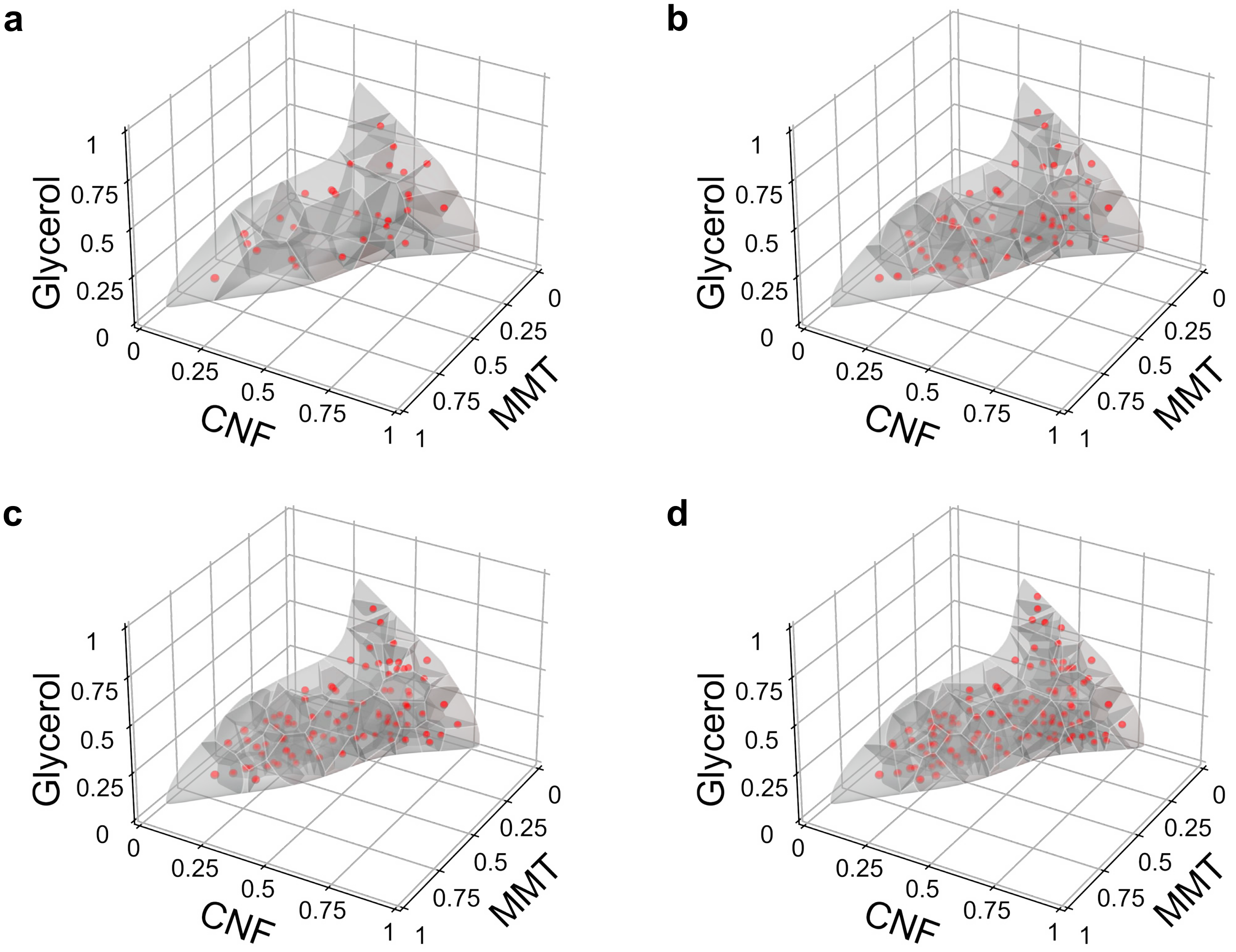

Fig. S11. 3D diagrams of Voronoi tessellation at different active learning stages. (**a**) After 3 active learning stages. (**b**) After 6 active learning stages. (**c**) After 9 active learning stages. (**d**) After 12 active learning stages.

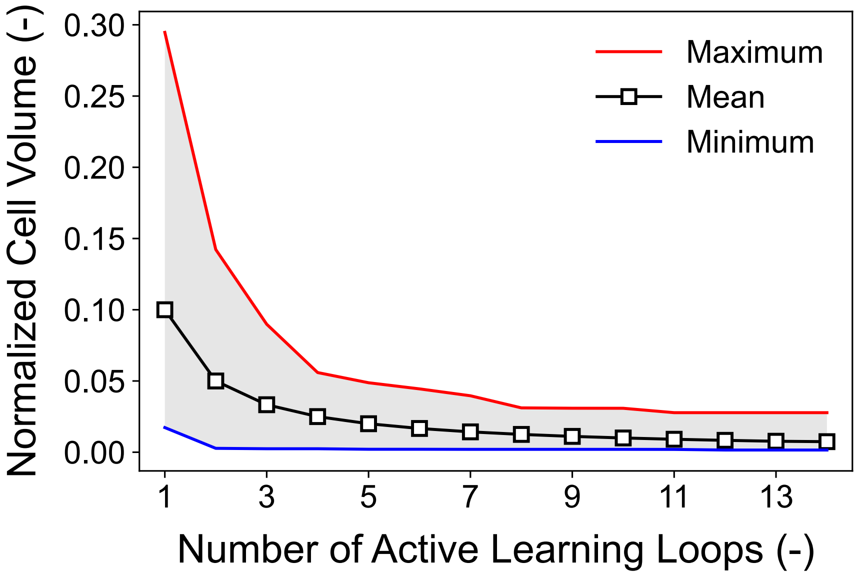

Fig. S12. Normalized average cell volumes of 3D Voronoi diagrams and their variances at different active learning stages. (**a**) As the number of active learning loops increases from 1 to 14, the normalized average cell volume decreased from 0.1 to 0.007, and (**b**) the corresponding variance decreased from 0.5 to 0.002, indicating that the prediction model was able to suggest the targeted data in different sub-regions instead of forming uninformative data clusters.

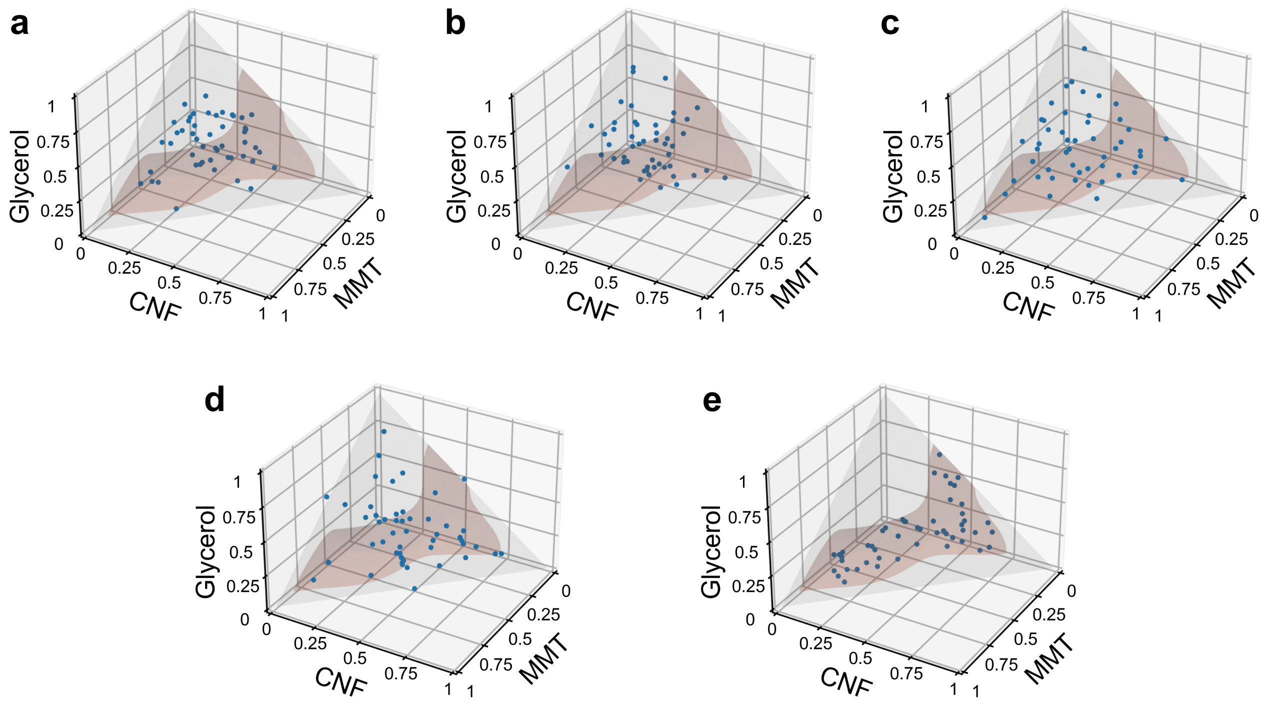

Fig. S13. Distribution profiles of data points collected from different sampling methods. (**a**) Random sampling, (**b**) Latin hypercube sampling, (**c**) distance sampling (by removing $\hat{\sigma}$ term from *A Score* acquisition function), (**d**) human intelligence sampling, and (**e**) active learning sampling. A total of 5 sampling cycles were performed, and 10 physical experiments were conducted in each cycle.

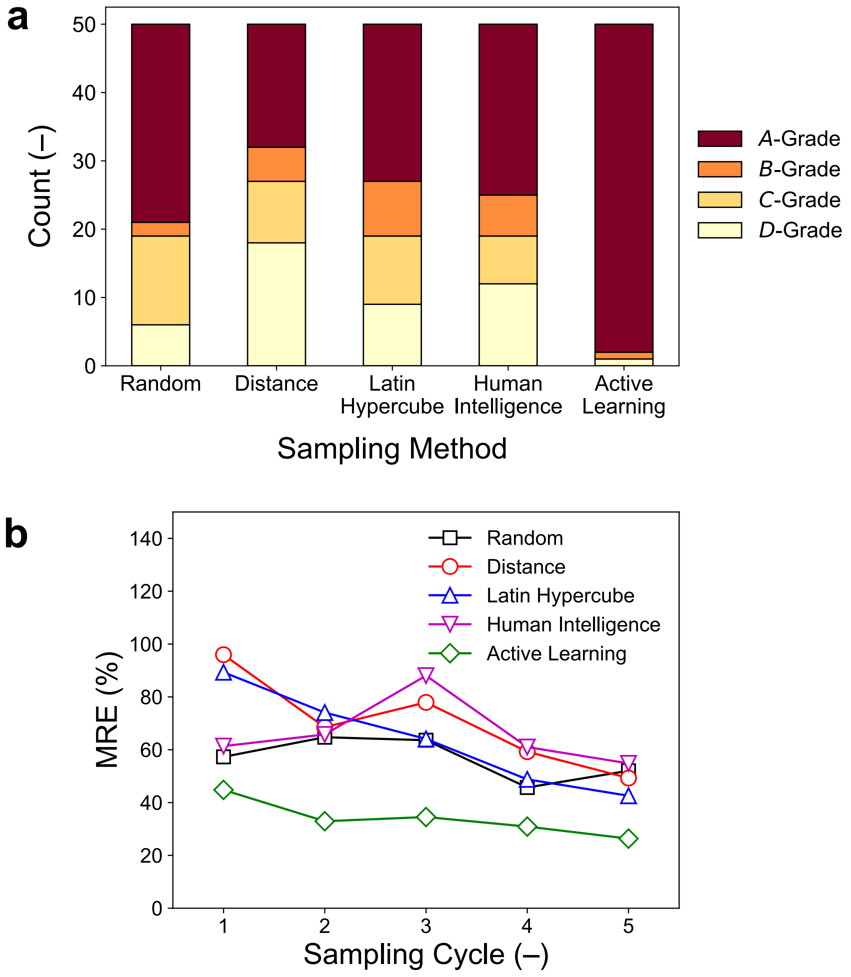

Fig. S14. Prediction model performance based on different sampling methods. (**a**) Quantity of nanocomposite films at different grades based on different sampling methods. The active learning sampling was able to recommend the MMT/CNF/gelatin/glycerol ratios with a >95% successful rate in producing *A*-grade nanocomposites. (**b**) MRE values of the prediction models based on different sampling methods. Trained by the data points collected from the active learning sampling, the prediction model demonstrated better learning efficiency and higher prediction accuracy, as evidenced by the lowest MRE of 26% after 5 cycles.

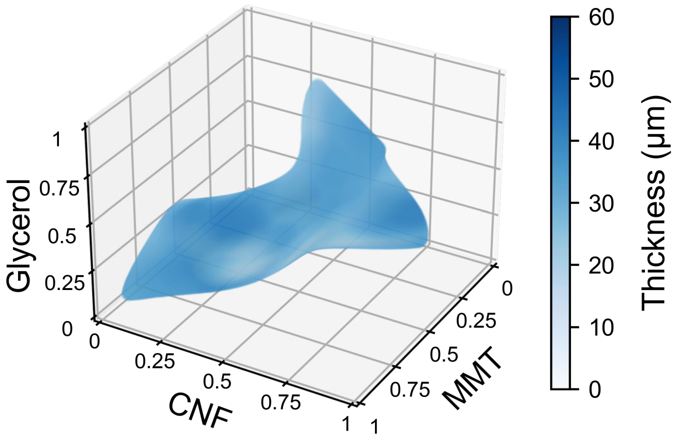

Fig. S15. Spatial distribution of model-predicted thickness labels. 3D heatmaps representing the spatial distributions of model-predicted thickness labels.

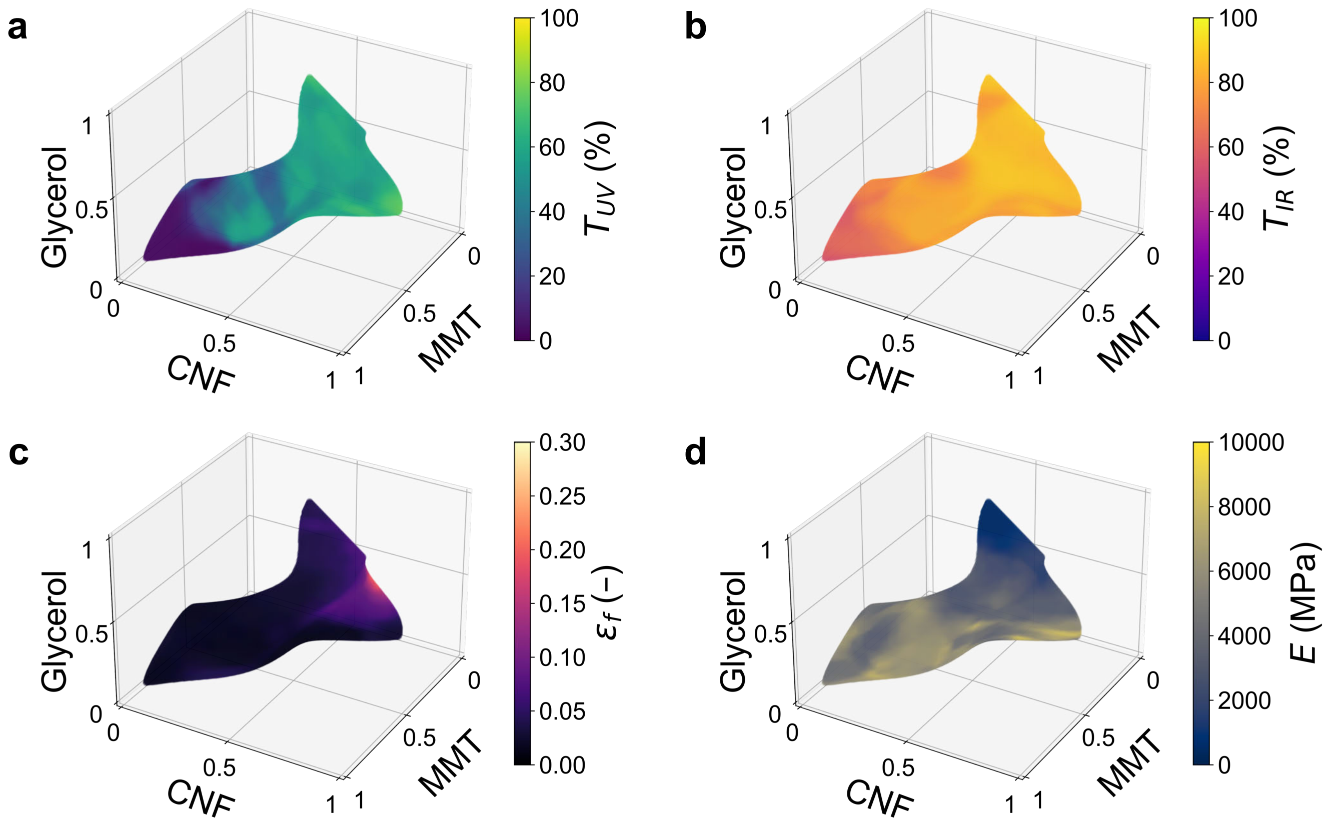

Fig. S16. 3D heatmaps representing the spatial distributions of model-predicted property labels. (**a**) T_{UV}, (**b**) T_{IR}, (**c**) ε_f, and (**d**) E labels.

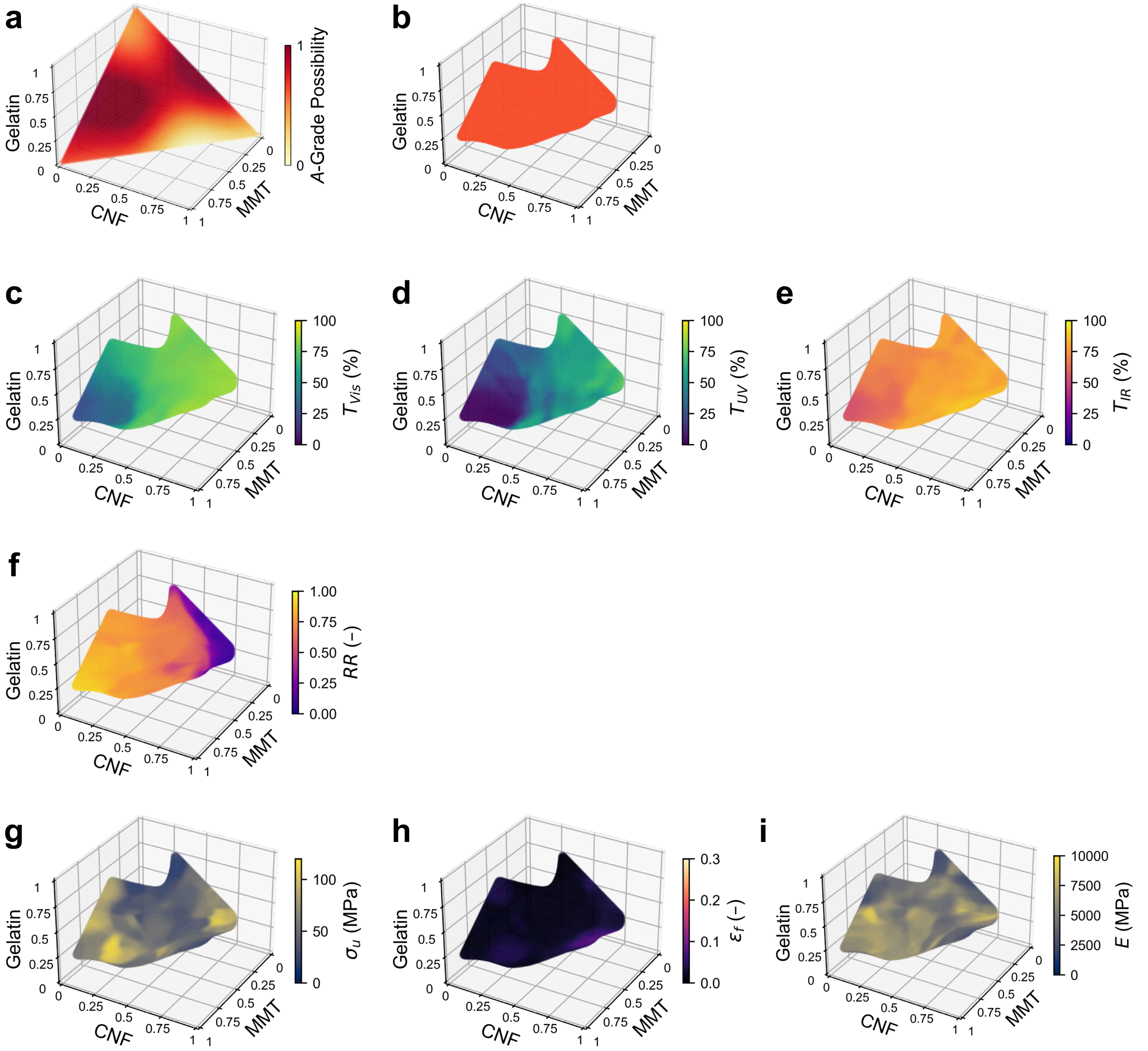

Fig. S17. 3D heatmaps with the gelatin loading shown on the *z*-axes. (**a**) 3D heatmap representing the possibility of obtaining an *A*-grade nanocomposite film at a specific MMT/CNF/gelatin/glycerol ratio. (**b**) Feasible design space. 3D heatmap representing the spatial distributions of model-predicted (**c**) T_{Vis}, (**d**) T_{UV}, (**e**) T_{IR}, (**f**) RR, (**g**) σ_u, (**h**) ε_f, and (**i**) E labels.

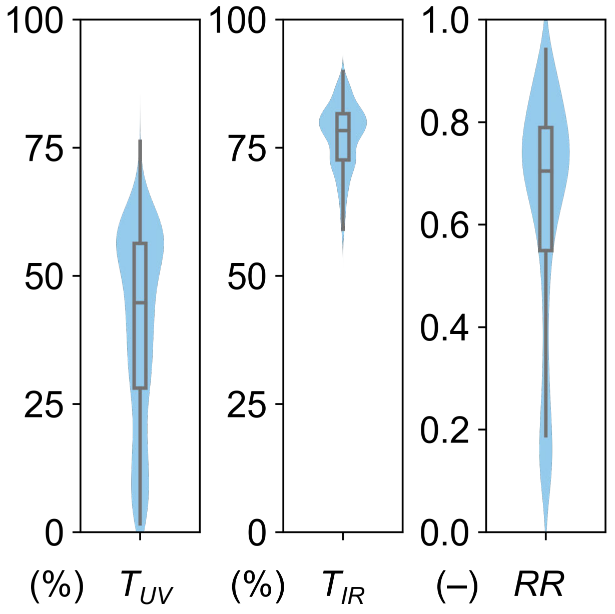

Fig. S18. Violin plots of T_{UV}, T_{IR}, and RR labels. Through simply adjusting the MMT/CNF/gelatin/glycerol ratios, the physicochemical properties of all-natural nanocomposites were highly tunable across wide ranges (e.g., 2% < T_{UV} < 76%, 54% < T_{IR} < 90%, 0 < RR < 1). The embedded box plot within each violin plot indicates the 25th and 75th percentiles with the median represented by the center line. Whiskers extend to 1.5 ×IQR from the box, n = 491,131.

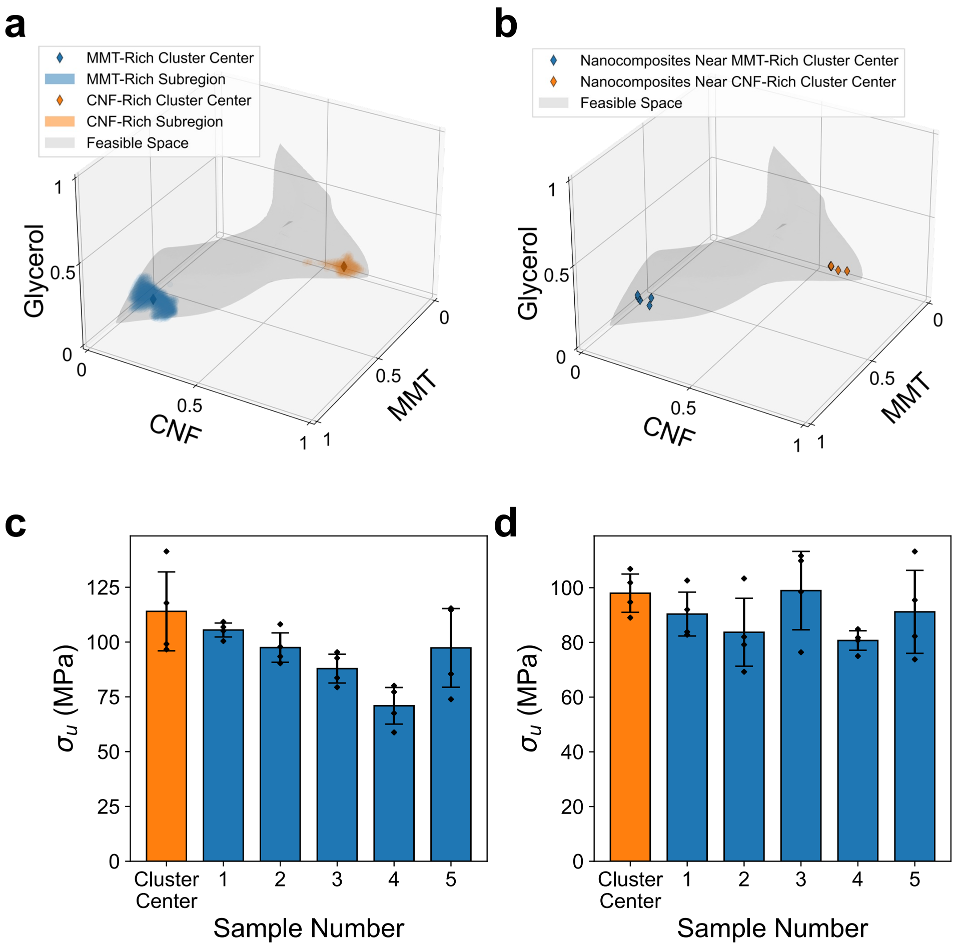

Fig. S19. Clustering analyses for discovering high-strength, all-natural nanocomposites. (a) By conducting clustering analyses, two cluster centers (e.g., MMT- and CNF-rich cluster centers) were identified, leading to the fabrication of all-natural nanocomposites with high σ_u. **(b)** Multiple model-suggested nanocomposites in MMT- and CNF-rich subregions were fabricated and characterized. **(c)** Comparison of σ_u labels of model-suggested nanocomposites near the MMT-rich cluster. Orange bar represents the all-natural nanocomposite at the MMT-rich cluster center. Blue bars represent the all-natural nanocomposites near the MMT-rich cluster center. **(d)** Comparison of σ_u labels of model-suggested nanocomposites near the CNF-rich cluster. Orange bar represents the all-natural nanocomposite at the CNF-rich cluster center. Blue bars represent

the all-natural nanocomposites near the CNF-rich cluster center. In each bar plots, data are presented as mean ± s.d., $n = 4$, with each independent experiment marked by a black diamond.

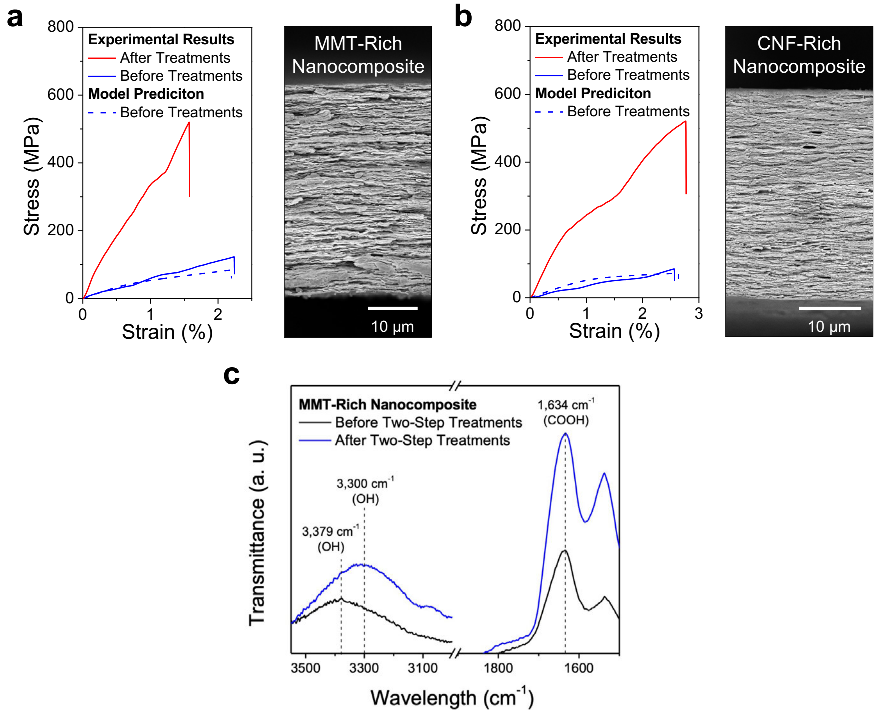

Fig. S20. Stress–strain curves, SEM images and FTIR spectra of all-natural nanocomposite before and after two-step treatments. Stress–strain curves of (**a**) the MMT-rich nanocomposites (MMT/CNF/gelatin/glycerol = 64.2/6.7/23.8/5.3) and (**b**) the CNF-rich nanocomposites (MMT/CNF/gelatin/glycerol = 3.7/61.8/28.4/6.1) before and after two-step treatments (ionic crosslinking and heat pressing). Solid lines represents the actual stress–strain curves, and dash lines represent the model-predicted stress–strain curves. The inset shows the SEM images of densified MMT- and CNF-rich nanocomposites. **(c)** After two-step treatments, it exhibited stronger (or shifted) peaks both at 1,634 cm^{-1} and 3,300 cm^{-1}, indicating that more hydrogen bonds were induced.

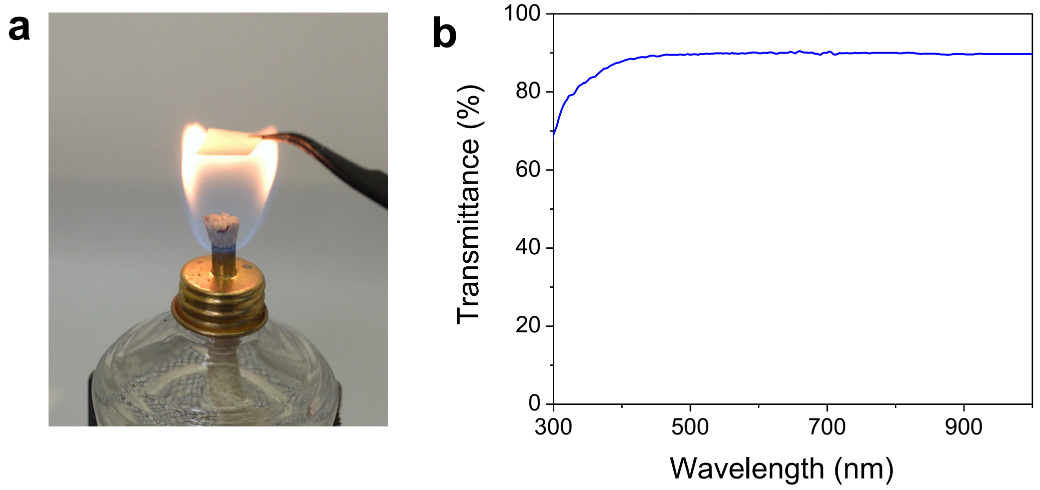

Fig. S21. Fire resistance and optical transmittance of densified all-natural nanocomposites. (a) Besides the superior σ_u value (468.6 ± 52.6 MPa), the MMT-rich nanocomposite (after two-step treatments) also showed a great fire resistance (with the RR value of 99.4 ± 7.2%). (b) Besides the superior σ_u value (463.0 ± 35.7 MPa), the CNF-rich nanocomposite (after two-step treatments) also showed a high visible-light transmittance (with the T_{Vis} value of 89.9%).

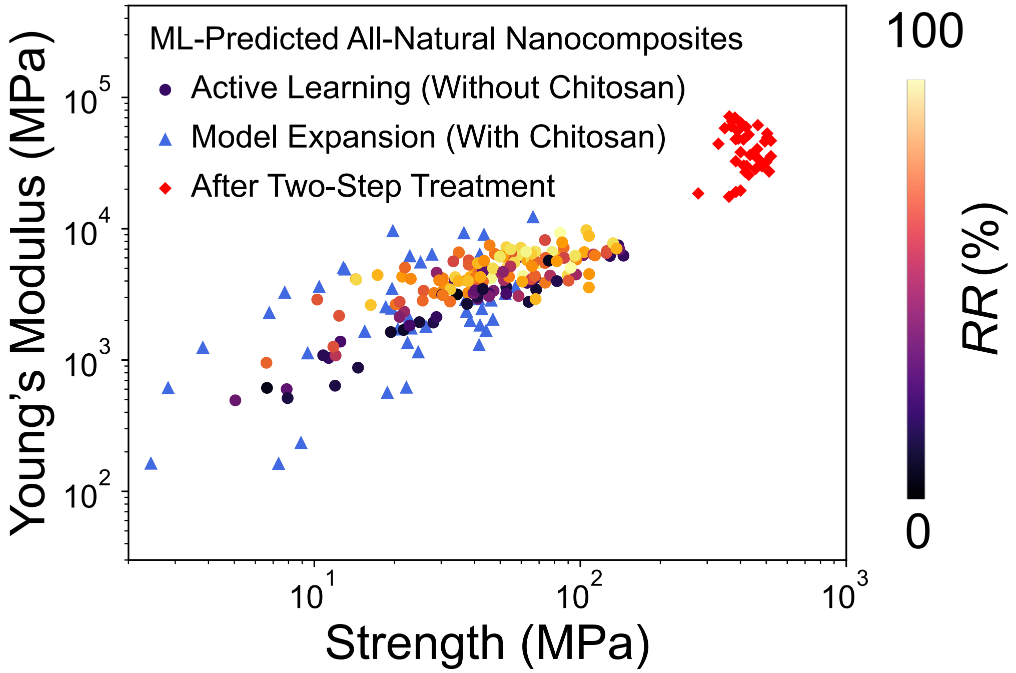

Fig. S22. σ_u–E plot of >150 all-natural nanocomposites with their *RR* values. The σ_u–E plot contains >150 all-natural nanocomposites fabricated during active learning loops, model expansion, and after two-step treatments, and the dot color represents the *RR* of each all-natural nanocomposite.

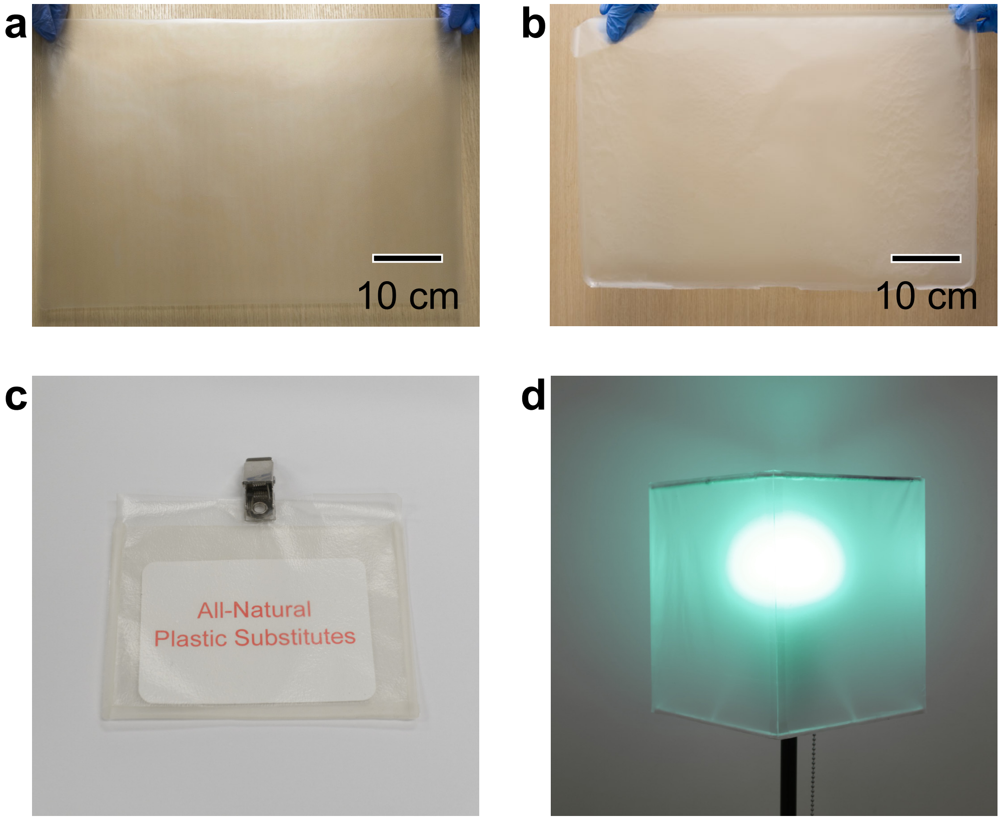

Fig. S23. Large-area production of all-natural substitutes with long shelf lives. (**a**)(**b**) Two all-natural substitute films with the MMT/CNF/gelatin/glycerol ratios of 6.0/48.8/32.2/12.0 and 26.9/23.2/40.1/9.7 were produced in large areas (with the dimensions of 53 cm × 38 cm). (**c**)(**d**) Photos of two all-natural substitutes (i.e., transparent badge holder and translucent lamp shading) after 6-month usage.

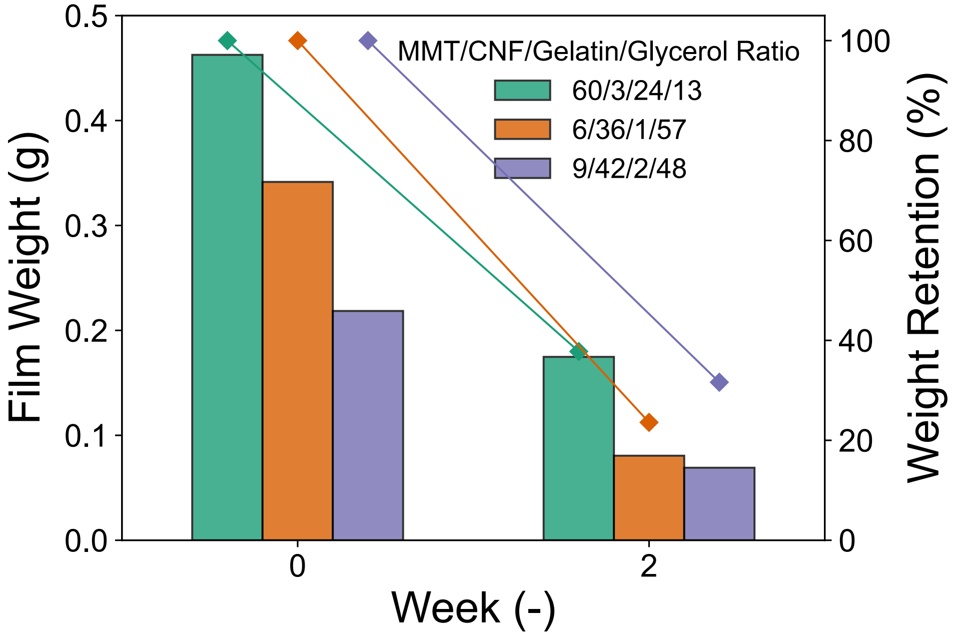

Fig. S24. Biodegradability of all-natural plastic substitutes. Sample weights of three all-natural plastic substitutes after the biodegradability tests for two weeks.

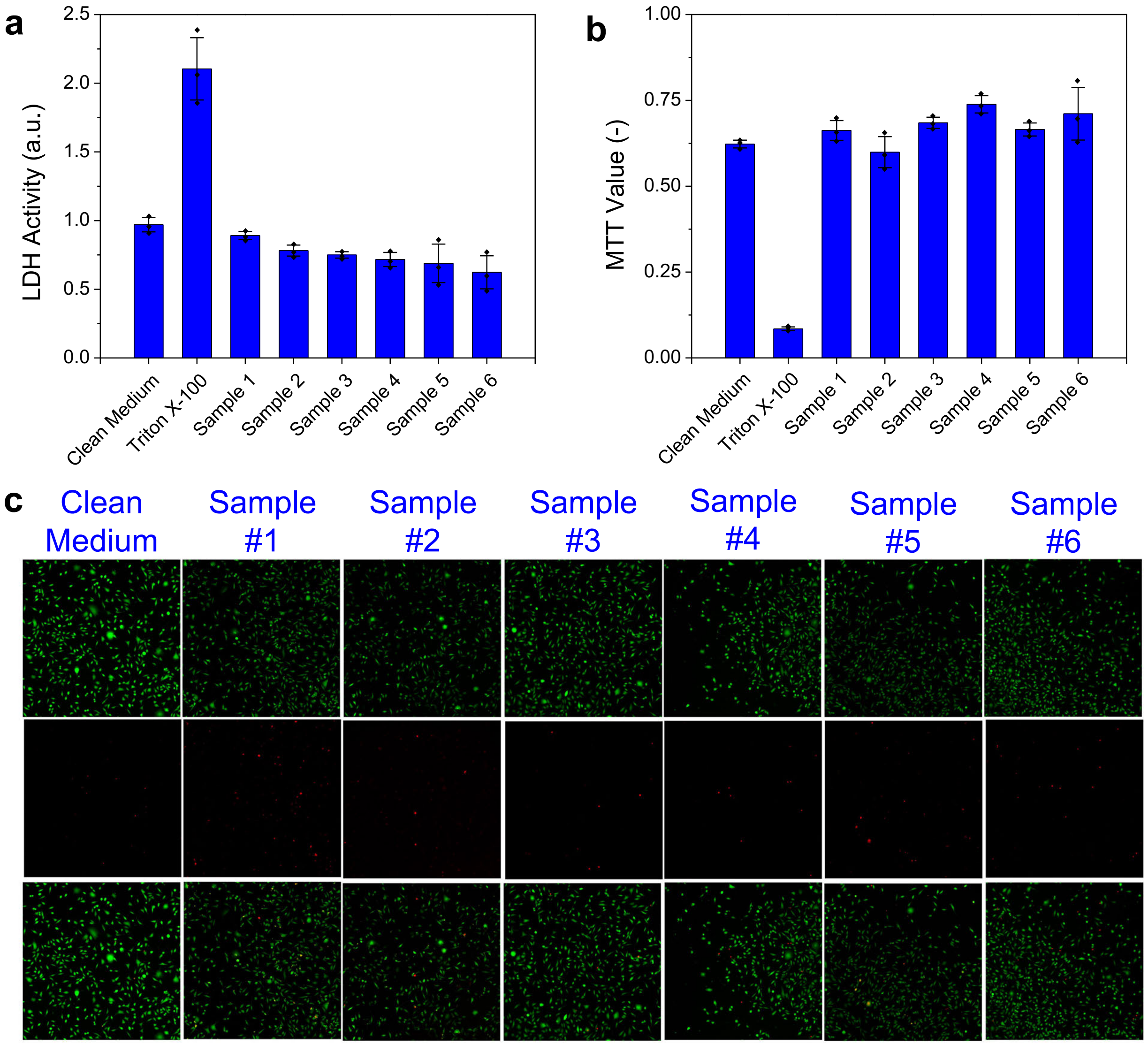

Fig. S25. Biocompatibility of all-natural plastic substitutes. (**a**) Lactate dehydrogenase (LDH) assays on L929 cells using clean culture medium, Triton X-100, and six extracts from different all-natural substitutes. (**b**) Tetrazolium-base (MTT) assays on L929 cells using clean culture medium, Triton X-100, and six extracts from different all-natural substitutes. In each bar plots, data are presented as mean ± s.d., n = 3, with each independent experiment marked by a black diamond. (**c**) Live/dead cell viability assays using six extracts from different all-natural substitutes. The MMT/CNF/gelatin/glycerol ratios of six all-natural substitutes are listed below:

Sample #1: 0.0/0.0/100.0/0.0; Sample #2: 0.0/0.0/90.0/10.0; Sample #3: 19.3/19.3/57.8/3.6; Sample #4: 18.6/18.6/55.8/7.0; Sample #5: 20.0/20.0/60.0/0.0; Sample #6: 33.3/33.3/33.4/0.0.

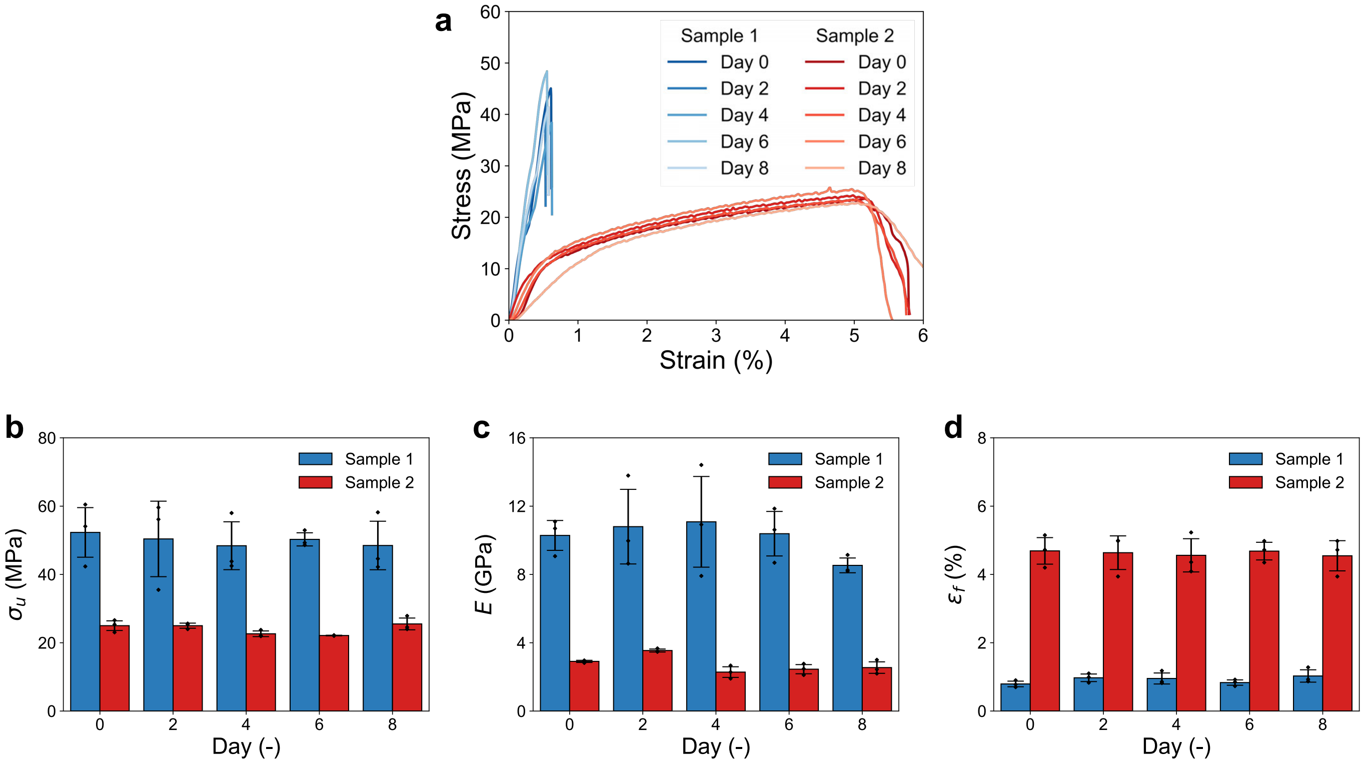

Fig. S26. Stability of all-natural plastic substitutes under sunlight. (**a**) Stress–strain curves, (**b**) σ_u, (**c**) E, and (**d**) ε_f of two all-natural plastic substitutes under sunlight for 8 days. Two all-natural substitutes with different MMT/CNF/gelatin/glycerol ratios (60/3/24/13 and 17/49/2/32) were subject to expose continuous sunlight for 8 days, and their mechanical properties remain similar over 8 days. In each bar plots, data are presented as mean ± s.d., n = 3, with each independent experiment marked by a black diamond.

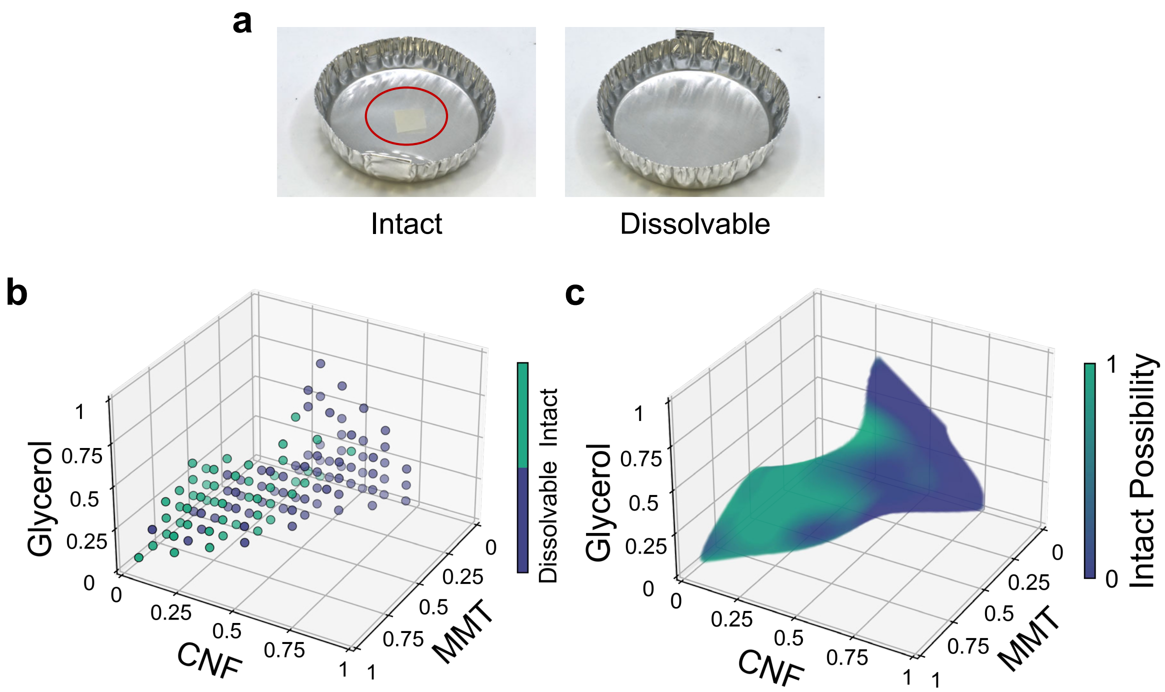

Fig. S27. Water stability levels of various all-natural nanocomposites. (**a**) Photos of all-natural nanocomposites with different water stability levels: left – intact, right – dissolvable. (**b**) 126 discrete data points representing the water stability of all-natural nanocomposites with varying MMT/CNF/gelatin/glycerol ratios. (**c**) A 3D heatmap representing the possibility of obtaining an intact-level all-natural nanocomposites at a specific MMT/CNF/gelatin/glycerol ratio.

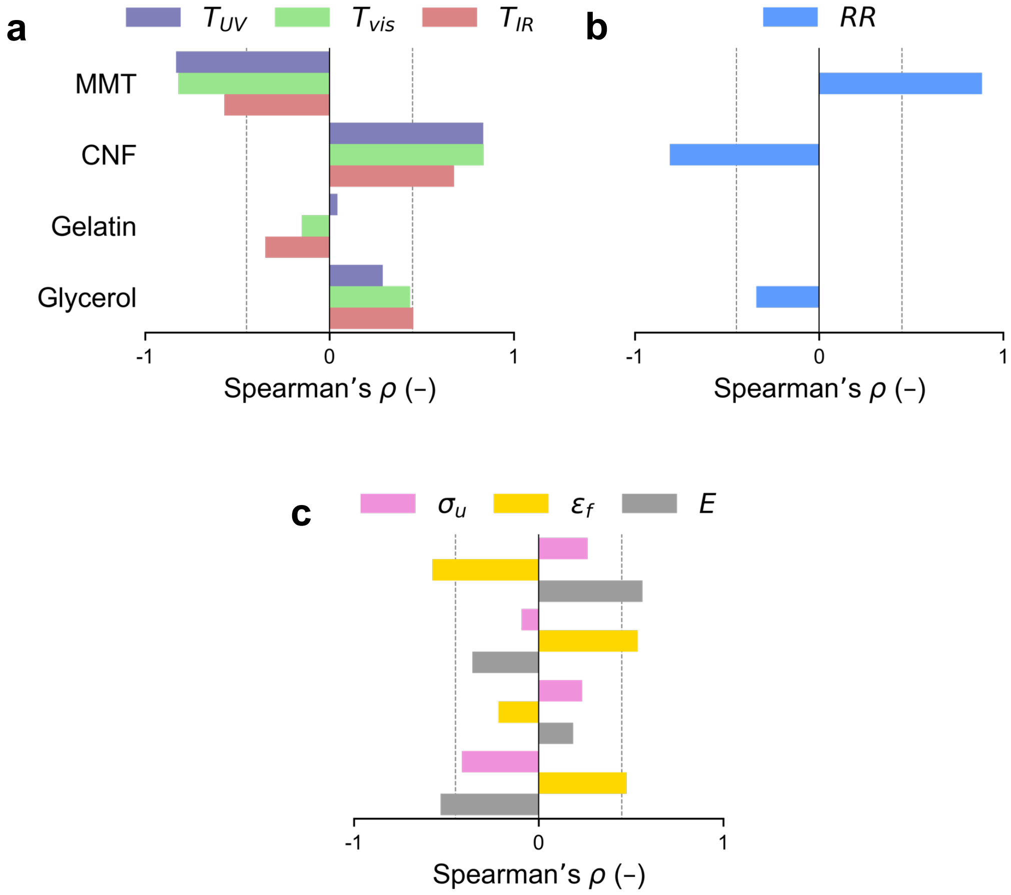

Fig. S28. Statistical analyses for all-natural nanocomposites between natural components and physicochemical properties. Spearman's rank correlation coefficients (Spearman's ρ) of MMT, CNF, gelatin, and glycerol loadings on (**a**) three spectral labels (i.e., T_{UV}, T_{Vis}, T_{IR}), (**b**) a "fire" label (i.e., RR), (**c**) three mechanical labels (i.e., ε_f, σ_u, E). As shown in **Fig. S28a**, the CNF loading showed positive and strong correlations with all three "spectral" labels, whereas the MMT loading showed negative and strong correlations with all three "spectral" labels, as the microscale platelets would induce light scattering. As shown in **Fig. S28b,** the MMT loading was positively correlated with the RR label, as the increase of MMT nanosheets would prevent oxygen penetration and reduce the flammability of all-natural nanocomposites significantly. As shown in **Fig. S28c**, the glycerol and CNF loadings were positively correlated with the ε_f label.

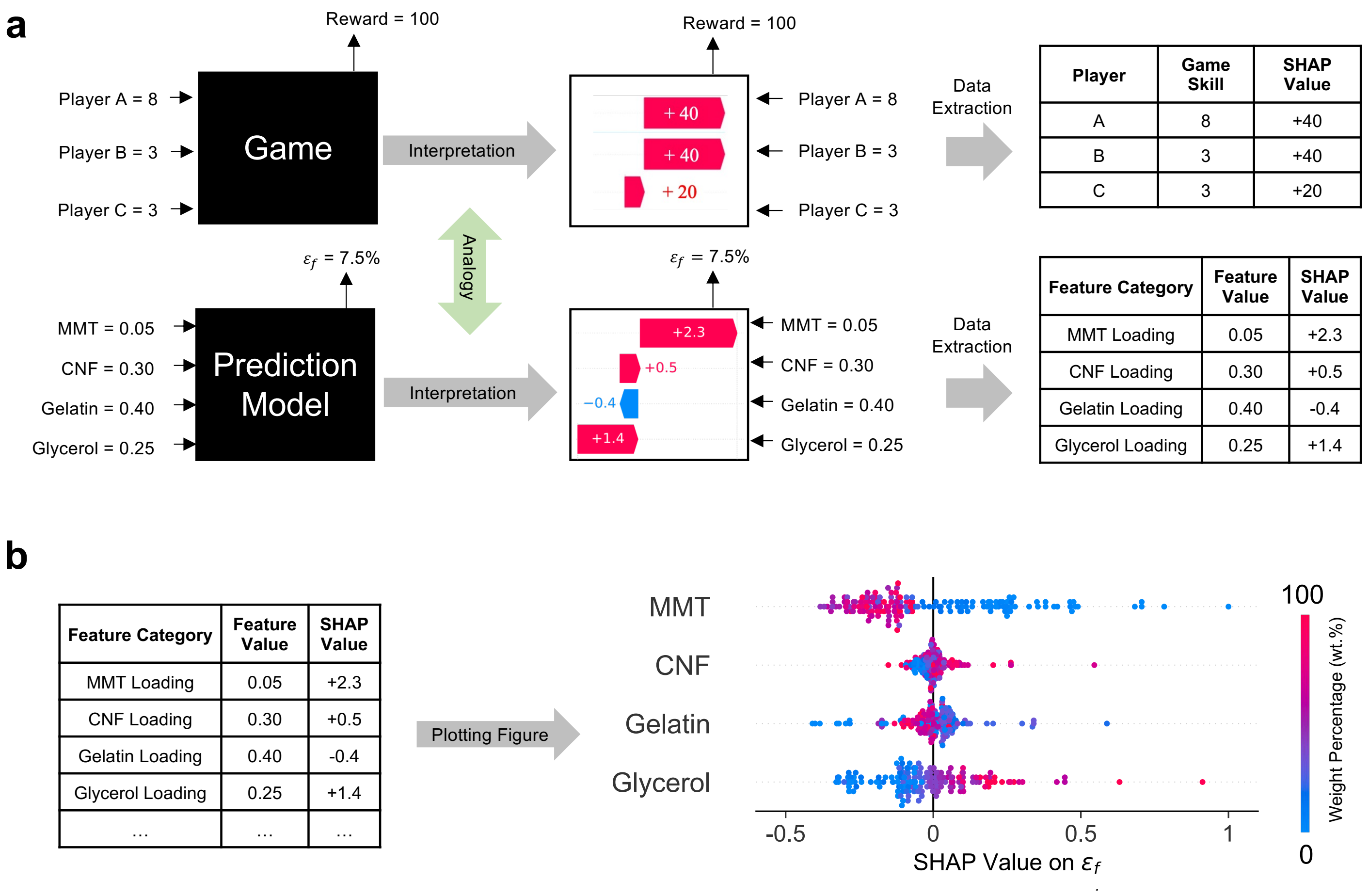

Fig. S29. Working mechanism of SHAP. (**a**) An analogy between the interpretation of a specific game and the interpretation of a prediction model. The blue and red bars are the SHAP values with positive as red and negative as blue. (**b**) Figure plotting to get the global interpretation of the prediction model by using the SHAP values of every feature for every data point.

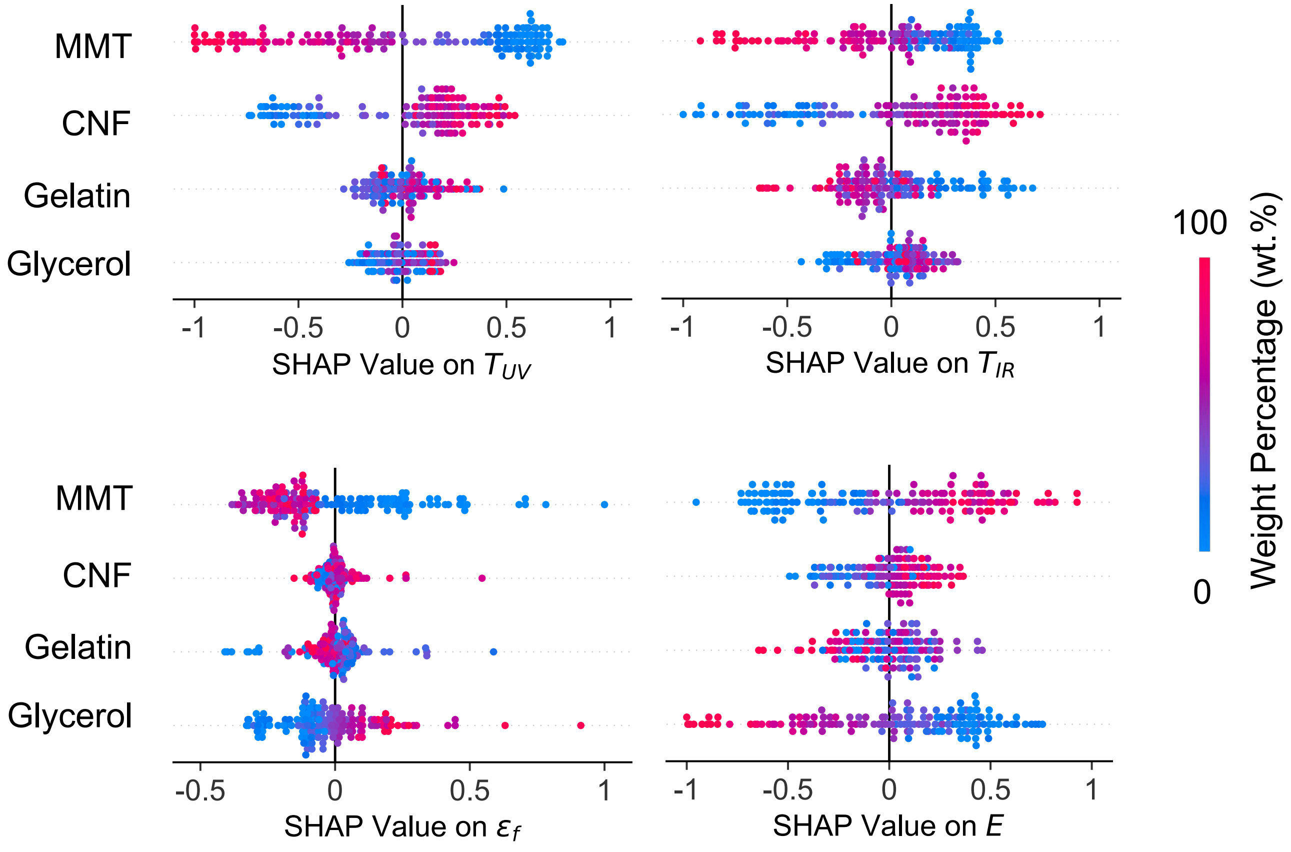

Fig. S30. SHAP values of CNF, MMT, gelatin, and glycerol loadings on various property labels, including T_{UV}, T_{IR}, ε_f, and E.

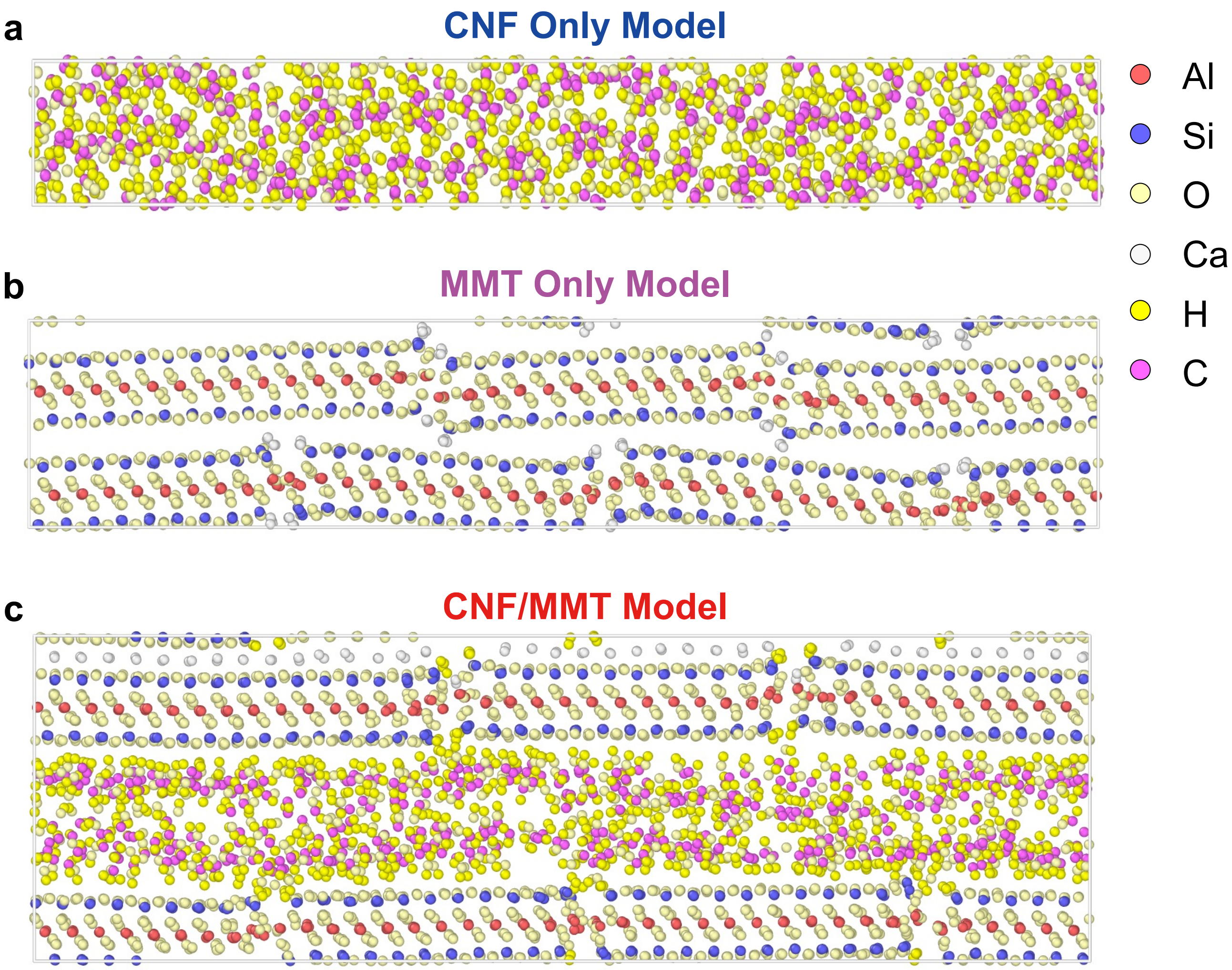

Fig. S31. Alternative presentations of CNF only, MMT only, and MMT/CNF models. Atomic structures of (**a**) CNF only, (**b**) MMT only, and (**c**) MMT/CNF models before and after tensile failure. Different colors indicate different atoms (red: aluminum; blue: silicon; light yellow: oxygen; silver: calcium; dark yellow: hydrogen; violet: carbon).

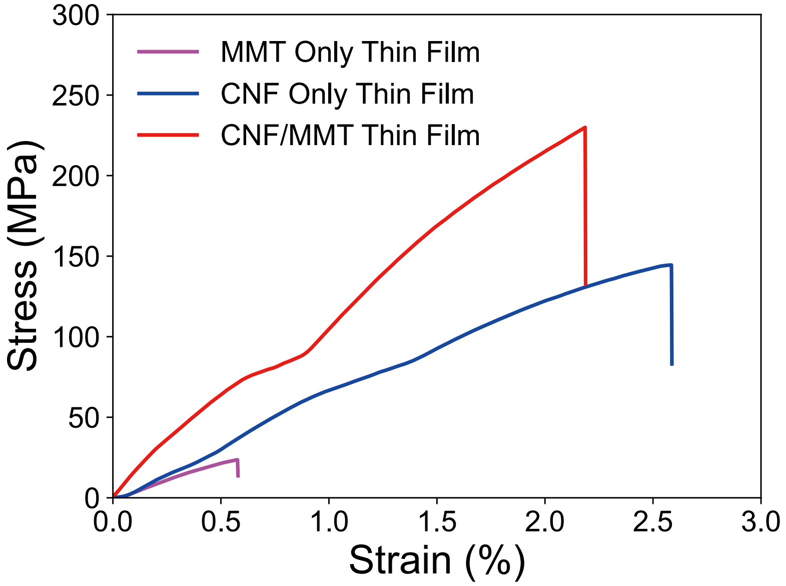

Fig. S32. Stress–strain curves of MMT only, CNF only, and MMT/CNF thin-film samples.

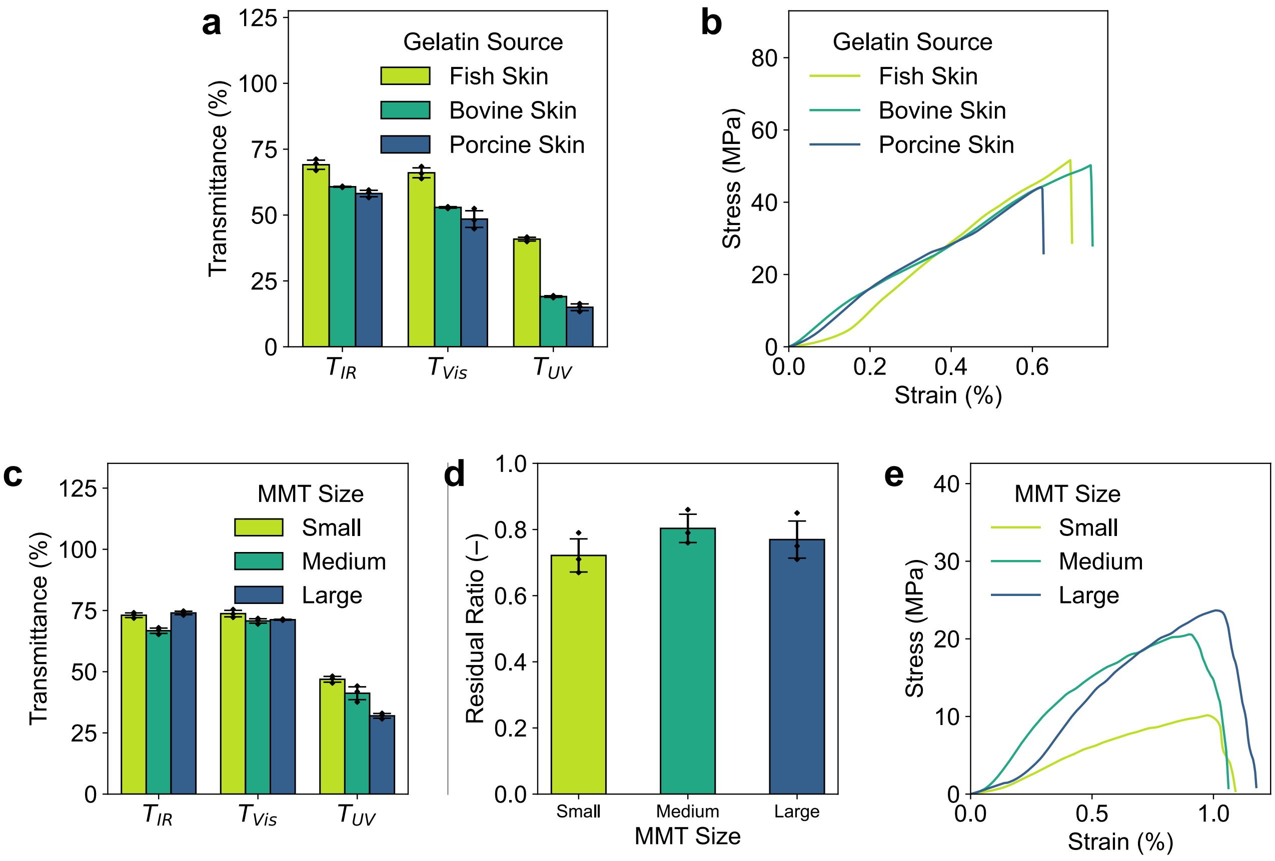

Fig. S33. Properties of all-natural nanocomposites with different gelatin sources and MMT sizes. (**a**) Optical and (**b**) mechanical properties of three all-natural nanocomposites at the same MMT/CNF/gelatin/glycerol ratio of 60.0/20.4/17.2/2.4 yet with different gelatin sources (from cold fish skin, porcine skin, and bovine skin). (**c**) Optical, (**d**) fire-resistant, and (**e**) mechanical properties of three all-natural nanocomposites at the same MMT/CNF/gelatin/glycerol ratio of 47.8/15.4/14.7/22.1 yet with different MMT dimensions. In each bar plots, data are presented as mean ± s.d., n = 3, with each independent experiment marked by a black diamond.

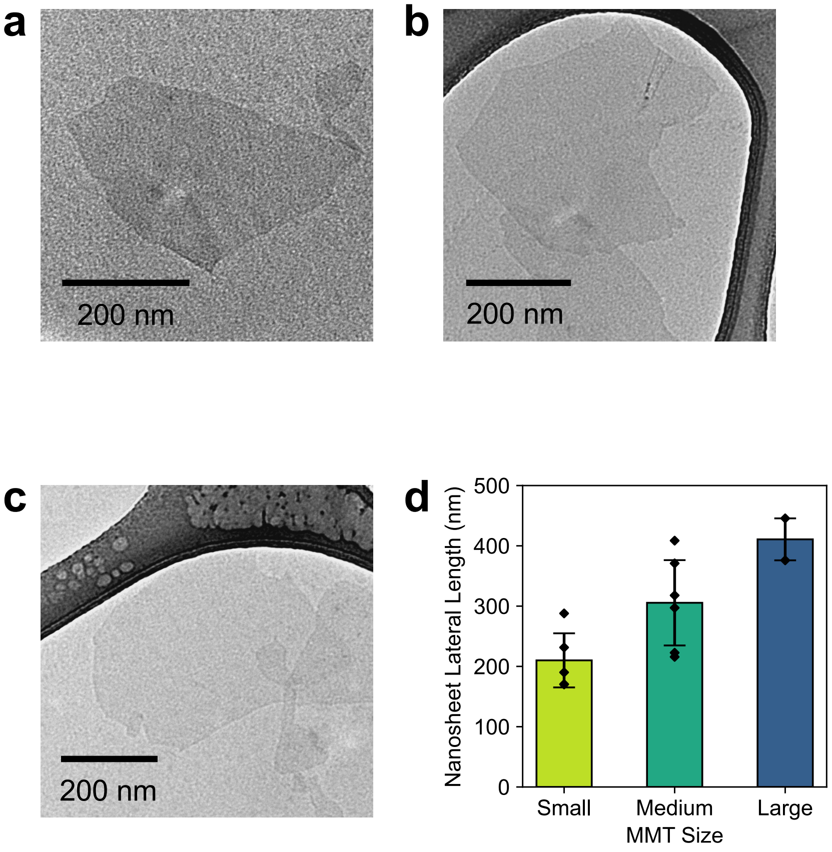

Fig. S34. Characterizations of MMT at different sizes. TEM images of (**a**) small-, (**b**) medium-, (**c**) large-sized MMT nanosheets. (**d**) Dimension distributions of three kinds of MMT nanosheets. Date are presented as mean ± s.d., with each independent experiment marked by a black diamond (n = 4 for small MMT size, 6 for medium MMT size, 2 for large MMT size).

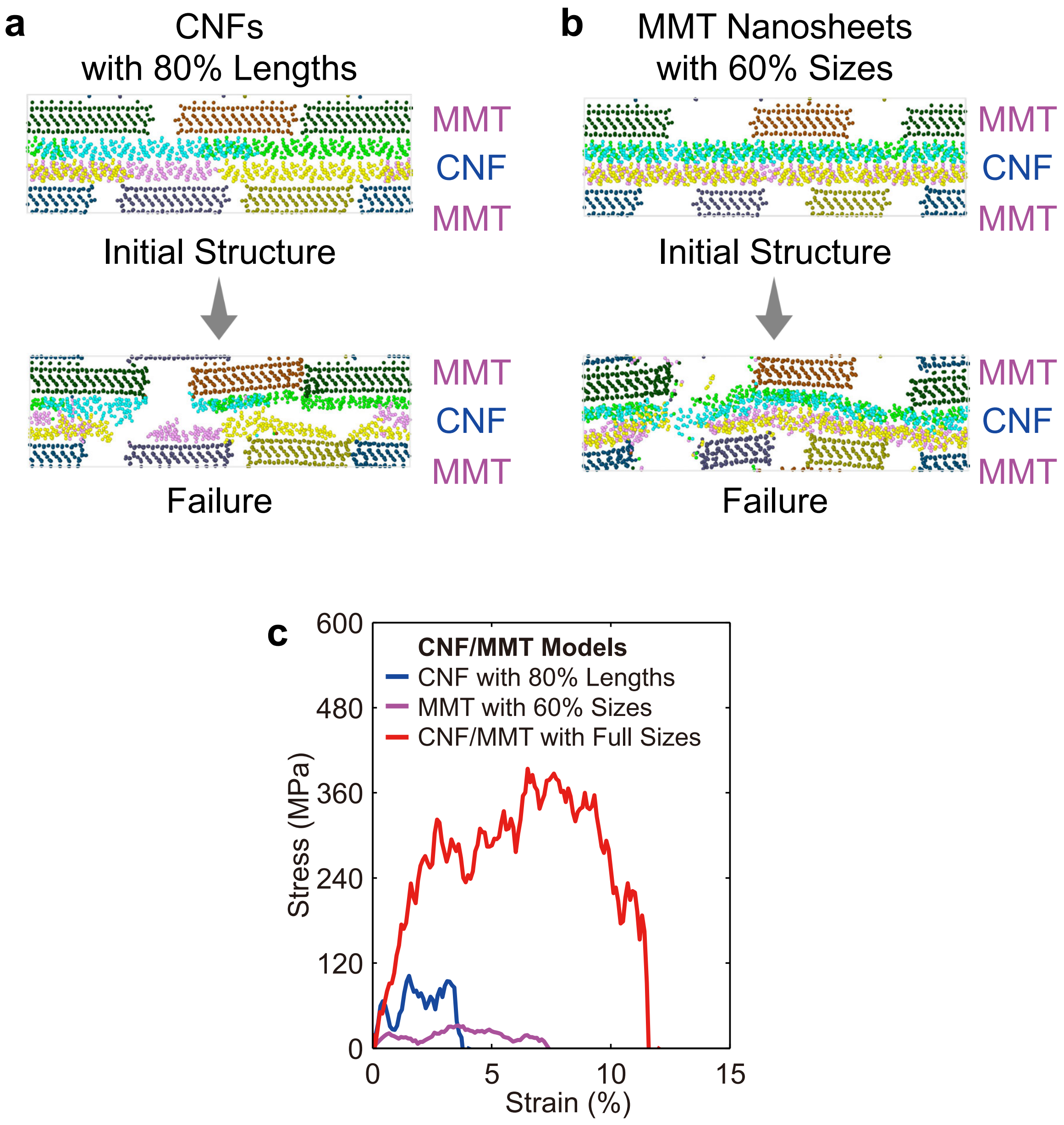

Fig. S35. MD simulations of CNF/MMT models with shorter CNF lengths and smaller MMT sizes. Atomic structures of CNF/MMT models with (**a**) CNF chains at 80% of their original lengths and (**b**) MMT nanosheets at 60% of their original sizes. (**c**) Simulated stress–strain curves of three MMT/CNF models with different CNF lengths and MMT sizes.

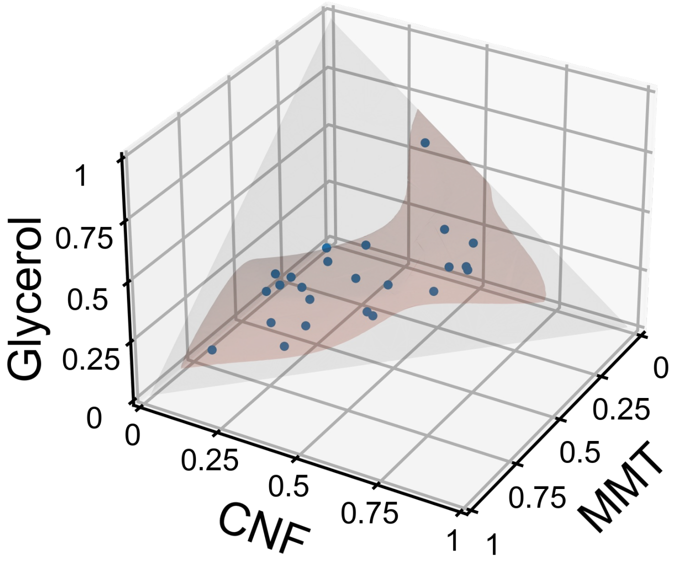

Fig. S36. 24 MMT/CNF/gelatin/glycerol ratios selected for sensitivity analyses.

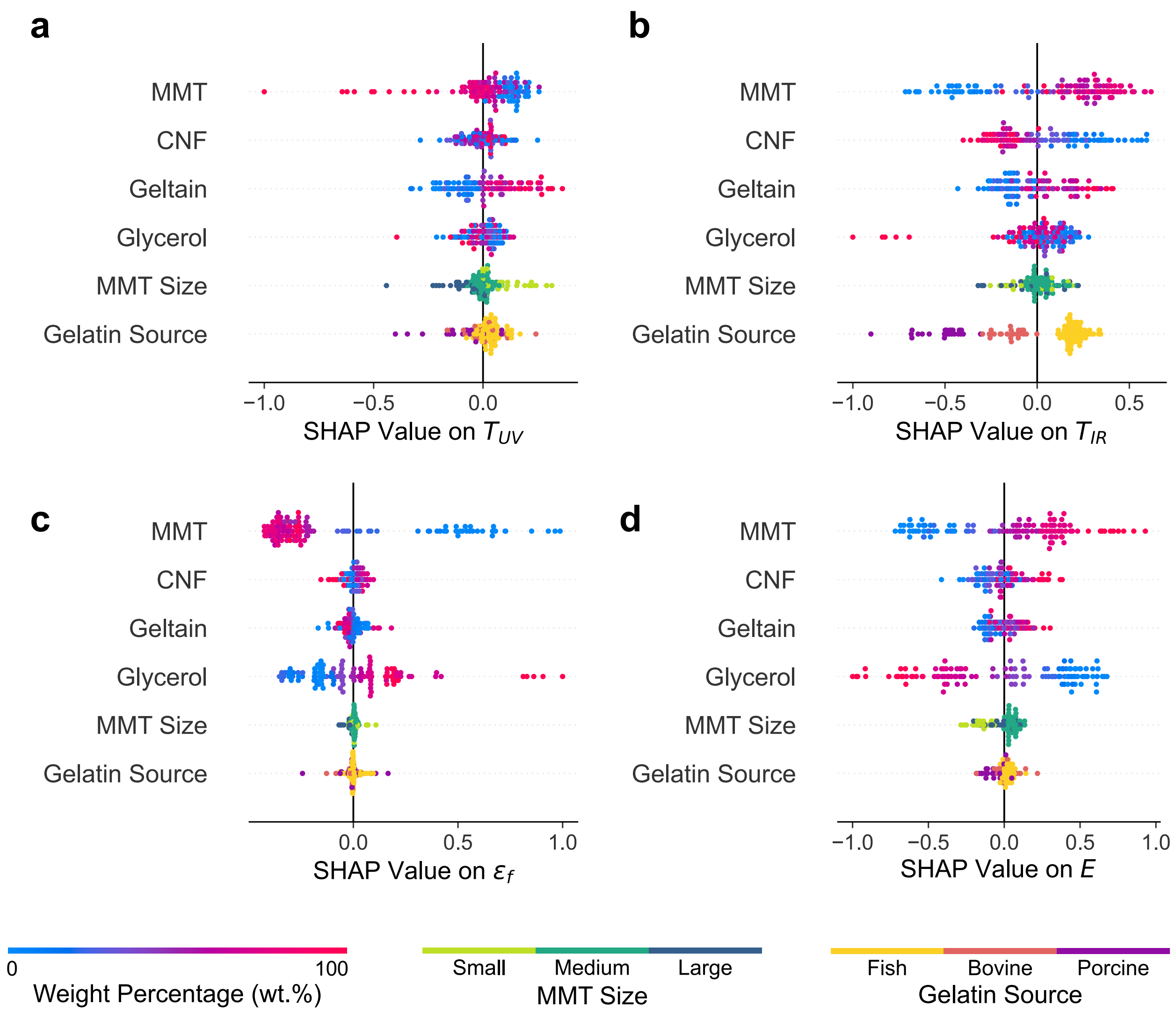

Fig. S37. Normalized SHAP values of MMT loading, CNF loading, gelatin loading, glycerol loading, gelatin source, and MMT size on T_{UV}, T_{IR}, ε_f, and E.

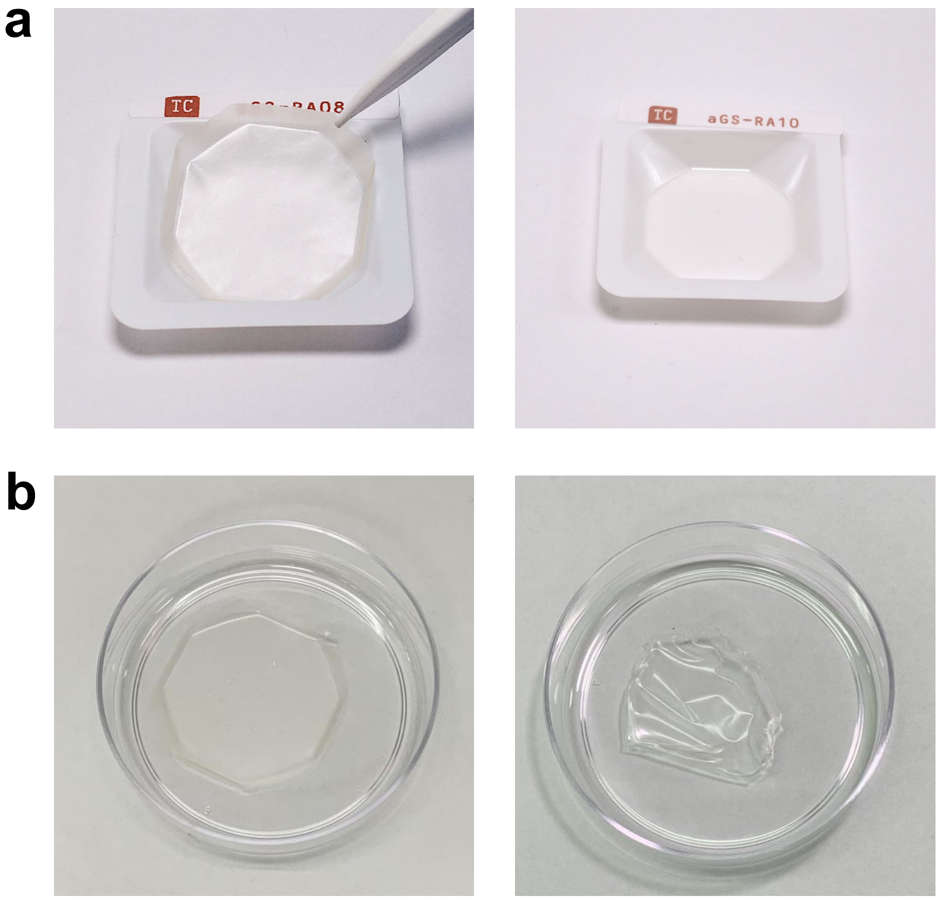

Fig. S38. Examples of all-natural nanocomposites with clearly classified detachability and flatness. (a) detachable and non-detachable, **(b)** flat and curved. At the stage of constructing the SVM model, a batch of 286 all-natural nanocomposites were fabricated. As shown in **Fig. S38a** and **Table S2**, a major portion of all-natural nanocomposites (209 out of 286 nanocomposites) were clearly classified as either “detachable” or “undetachable” features without conducting any adhesion tests. Also, as shown in **Fig. S38b** and **Table S2**, out of 184 detachable nanocomposites, 134 of them were easily classified into either the “flat” or “curved” categories without conducting laser conformal imaging. Therefore, only a portion all-natural nanocomposites (77 out of 286 nanocomposites for detachability, and 50 out of 184 for flatness tests) were required to conduct

further adhesion tests and laser conformal imaging to determine their classification at the boundary of the feasible design space.

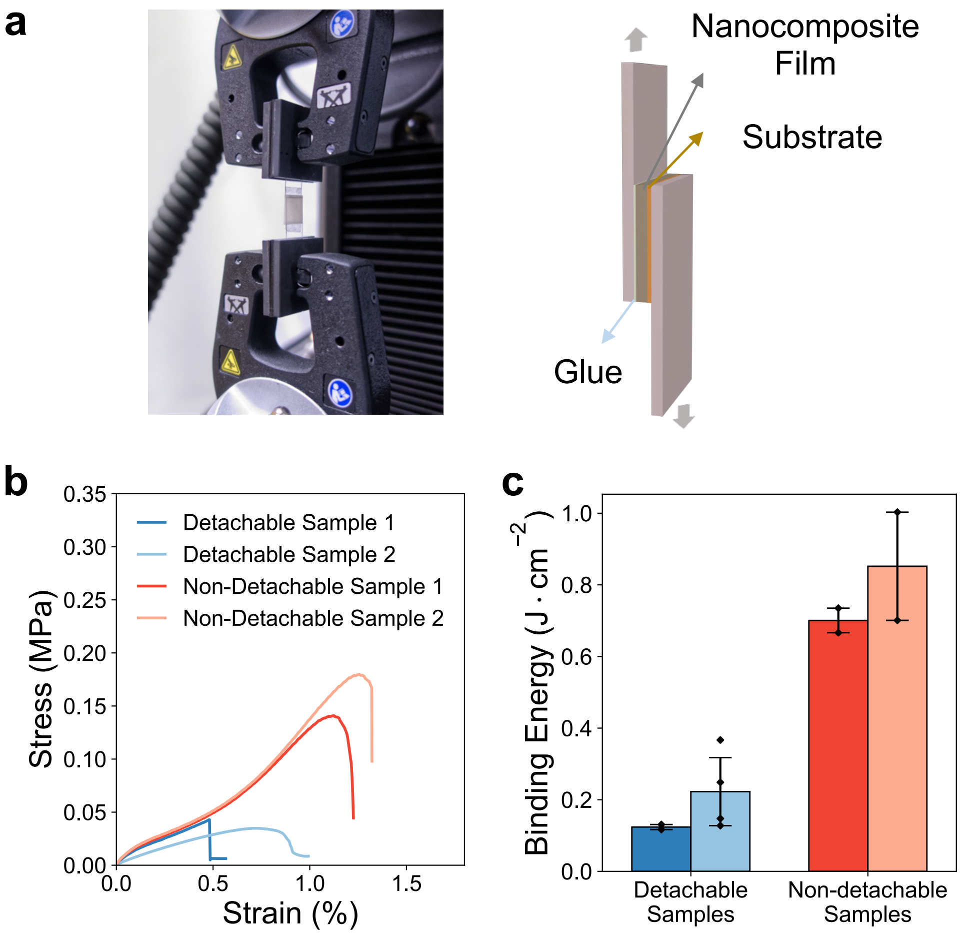

Fig. S39. Mechanical delamination tests. (**a**) Actual setups and schematic illustrations and of the mechanical delamination tests. (**b**) Mechanical delamination test results of detachable and non-detachable samples. (**c**) Binding energies of detachable and non-detachable samples. Data are presented as mean ± s.d., with each independent experiment marked by a black diamond ($n = 4$ for Detachable Sample 2, and $n = 2$ for other samples).

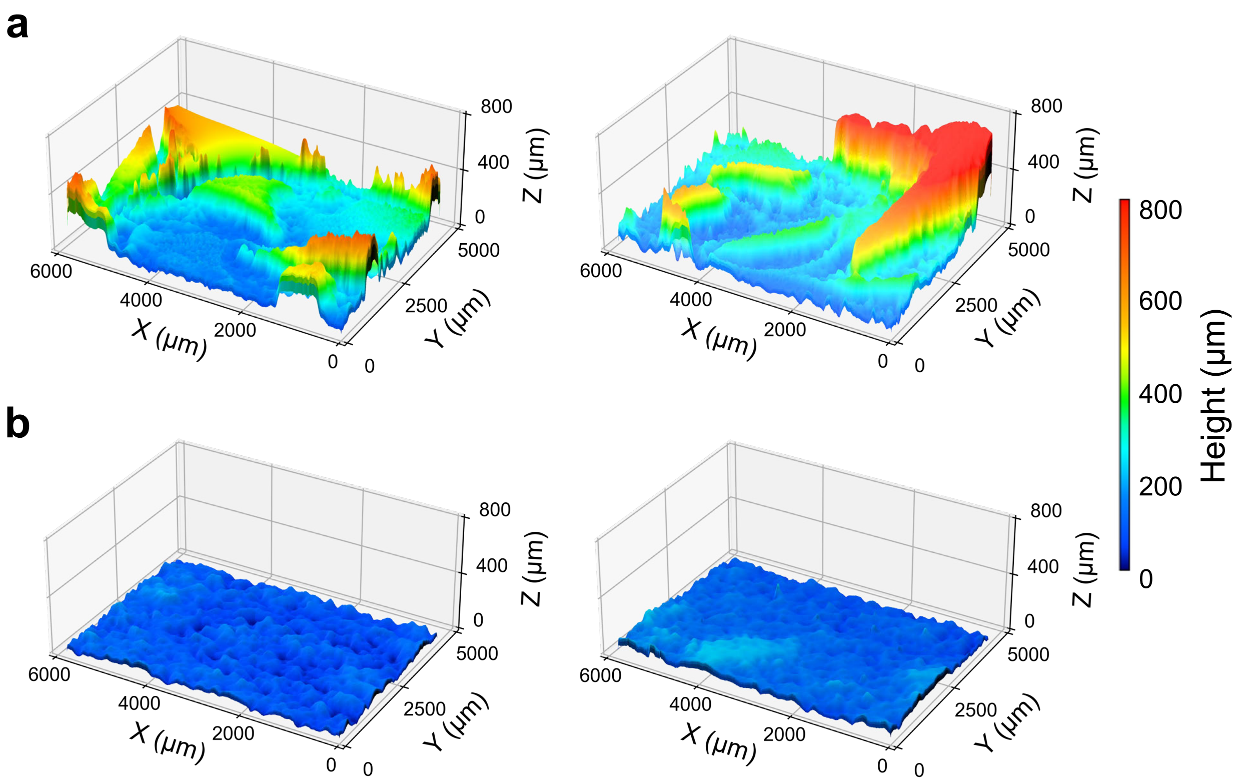

Fig. S40. Flatness classification of nanocomposite films. Confocal laser images of (**a**) curved and (**b**) flat nanocomposite films.

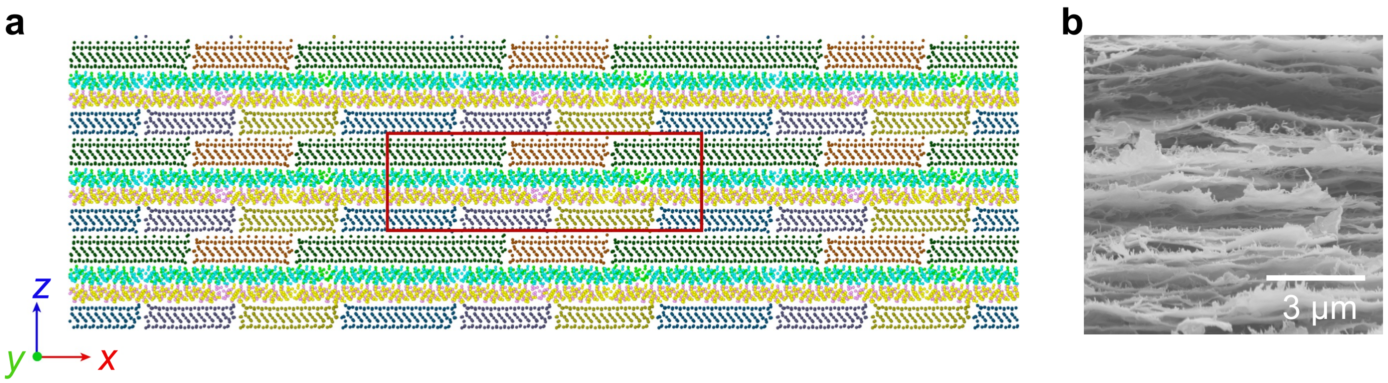

Fig. S41. Construction of a MMT/CNF model. (**a**) Atomic structure of a MMT/CNF model with periodical boundary conditions. The red rectangle represents the minimum repetitive unit presented in Fig. 5. (**b**) SEM images of a MMT/CNF thin film. It is important to note that the SEM sample was fractured within liquid nitrogen, a procedure that made the CNFs more distinguishable.

Note S1. Selection of natural components for all-natural plastic substitutes.

Natural components can serve as important renewable and environmentally degradable resources. The initial selection of 2D MMT nanosheets for the fabrication of all-natural plastic substitutes was due to the frequent use as low-cost, sustainable nanofillers within polymeric matrices.[1] As shown in **Fig. S1**, the MMT only thin films were rigid and lacked mechanical resilience, largely attributed to the weak interactions between nanosheets. Next, to stabilize these MMT nanosheets, we incorporated gelatin as the second building block. Gelatin has long been explored as a safe and biodegradable component for plastic replacement, which is often involved to stabilize and strengthen the assembled nanocomposites.[2] Adjusting the MMT/gelatin ratios yielded the all-natural substitutes with tunable tensile strengths, all of which were higher than the tensile strength of the MMT only one. Furthermore, inspired by several important articles, integrating 1D CNFs, which contained with rich functional groups, could boost hydrogen bonding interactions and improve the tensile strengths of nanocomposites. Also, CNF is the major component of natural plant fibers and possesses a high tensile strength, a high modulus, a low coefficient of thermal expansion, and an easily modifiable surface.[3] In our experiments, introducing CNFs to the MMT/gelatin matrix further improved the tensile strength of resulting nanocomposites, outperforming the binary cases. However, these nanocomposites still exhibited limited the ultimate strains <2.5%. Therefore, glycerol was selected as the fourth building blocks to enhance the flexibility and ultimate strains of assembled nanocomposites.[4] In this work, four natural components were selected, including MMT nanosheets, CNFs, gelatin, and glycerol.

Note S2. Estimated number of experiments required to build an extensive dataset for all-natural nanocomposites.

Three degrees of freedom (DOF) were recognized in the compositions of all-natural plastic substitutes, including CNF, MMT, and gelatin loadings. Once these three loadings were fixed, the glycerol loading would be determined. If we set 2.0 wt.% as the step size for all CNF, MMT, gelatin, and glycerol loadings, the total steps are calculated to be 23,426 for varying the MMT/CNF/gelatin/glycerol ratios across three DOFs. Also, 9 property labels (including T_{UV}, T_{Vis}, T_{IR}, RR, σ_u, ε_f, E, α, and β) are required to be collected for each all-natural plastic substitute from its transmittance spectrum, fire test result, and stress–strain curve.

Note S3. Influence of evaporation substrates on the grades of nanocomposite films.

As shown in **Fig. S6a**, when we switched from hydrophobic polystyrene substrates to polycarbonate petri dishes, fewer nanocomposite films were detached and achieved *A*-grade conditions. As shown in **Fig. S6b**, the use of hydrophobic polystyrene substrates led to 32% of *A*-grade nanocomposites (18 out of 56 MMT/CNF/gelatin/glycerol ratios). In contrast, the use of polycarbonate petri dishes yielded only 18% *A*-grade nanocomposites (10 out of 56 ratios, **Fig. S6c**). As a result, using polycarbonate petri dishes led to a smaller feasible design space (**Fig. S6d**). To broaden the feasible design space, we used the hydrophobic polystyrene substrates for preparing all-natural plastic substitutes in subsequent active learning loops.

Note S4. Training of a support-vector machine (SVM) classifier.

To ensure the prediction model to suggest the MMT/CNF/gelatin/glycerol ratios toward *A*-grade nanocomposite films, a SVM classifier was trained to categorize the all-natural nanocomposites into four cases (i.e., *A*-, *B*-, *C*-, and *D*-grades). Three steps were involved in constructing a SVM classifier, including (1) selecting a kernel function, (2) optimizing SVM hyperparameters, and (3) retraining the SVM classifier with all 286 data points.

In this work, as the collected grades were not shown to be linear, we decided to use a kernel function to map low-dimension data points into a higher dimensional feature space to find the optimal hyperplanes with maximal margin distances.[5] For the first step, a radial basis function (RBF) was selected as the kernel function to deal with the non-linear data points. Afterwards Bayesian optimization involving Gaussian processes and a 5-fold cross validation was used to adjust the hyperparameter values.[6] For the last step, the optimal SVM classifier was trained by inputting 286 grades and achieved a testing accuracy of 93% (using 35 testing data points). The open source code to train the SVM model in Python is provided in **GitHub** (https://github.com/chentl/MatAL/blob/master/design_boundary.ipynb).

Note S5. Necessity of multi-stage ML framework.

To demonstrate the necessity of each method used in the multi-stage ML framework, detailed justifications are provided here to explain their purposes and why they are superior to other simple/standard methods.

SVM classifier: SVM classifier served as a critical unit in the active learning loops, which performed as a screening layer and drove the prediction model to only recommend the MMT/CNF/gelatin/glycerol ratios with high possibilities of getting *A*-grade nanocomposite films. Unlike other data-rich systems with higher tolerance of experiment failure, it would take much time and effort to re-do the fabrication of all-natural nanocomposites, if the prediction model suggests the MMT/CNF/gelatin/glycerol ratios that are on the margin of the discrete phase diagram. Therefore, SVM is necessary to transform the discrete grade diagram into a possibility heat map.

ANN committee: According to **Fig. 2e**, the prediction model based on linear regression cannot accurately predict the property labels of all-natural nanocomposites and showed high MREs of 38.3% after active learning loops. In comparison, the prediction model (consisting of a ANN committee) demonstrated a much lower MRE of 17.0% after active learning loops, clearly demonstrating the necessity of a ANN committee for such a non-linear and multi-DOF system.

Data augmentation: The implementation of data augmentation is to address the major challenge of data scarcity and resolve the issues of potential overfitting, especially upon the use of a small dataset. The data augmentation methods (e.g., UIP method) are used to create adequate virtual data points and enable the prediction model to be trained with both real and virtual data points, resulting in a higher prediction accuracy. As shown in **Fig. 2f**, after data augmentation, the model prediction accuracy was improved in terms of MRE from >55.0% to 17.0%.

Overall, we believe that the multi-stage ML framework composed of SVM classifier, active learning (with a ANN committee), and data augmentation can create strong synergy to address the data scarcity challenges of building the prediction model for all-natural nanocomposites, where simple methods or single ML tool/method cannot achieve.

Note S6. User Input Principle (UIP) method.

In order to address potential overfitting issues upon the use of a small dataset, the data points collected during active learning are augmented by using a widely used method, User Input Principle (UIP). The UIP method is based on the natural principles proposed by expert users. For instance, the property labels of an all-natural nanocomposites stay approximately constant over very small variations across specific composition label(s). As shown in **Fig. S9**, when the MMT/CNF/gelatin/glycerol ratio varied from 1.3/48.8/24.9/25.0 to 4.3/47.9/23.9/23.9, the resultant all-natural nanocomposites exhibited similar property labels. Also, there were inevitable measurement variations of 9 property labels. As shown in **Fig. S10**, even under the same composition labels, the collected property labels could have 10%–20% variances across multiple all-natural nanocomposite replicates. In this work, based on 135 data points collected during active learning, we used the UIP method to synthesize 1,000-fold virtual data by introducing Gaussian noises into all composition and property labels. The open source code to implement the UIP method in Python is provided in **GitHub** (https://github.com/chentl/MatAL/blob/master/data_augmentation.ipynb).

Note S7. Calculation of *A Score* acquisition function.

A suitable acquisition function was introduced in the active learning loops to suggest the targeted data points with the highest uncertainty in the feasible design space. We defined the acquisition function as *A Score* in **Equation S1**,

$$A\,Score = L_2 \times \hat{\sigma} \qquad \textbf{(S1)}$$

, where L_2 denotes the shortest mathematical distance (also called Euclidian distance) between current composition labels (within the dataset of prediction model) and targeted composition labels (not yet included in the dataset of prediction model). In particular, L_2 is calculated by **Equation S2**,

$$L_2 = \sqrt[2]{\min_{i \in N}\left[\left(\begin{bmatrix} CNF_i \\ MMT_i \\ GEL_i \\ GLY_i \end{bmatrix} - \begin{bmatrix} CNF_j \\ MMT_j \\ GEL_j \\ GLY_j \end{bmatrix}\right)^2\right]} \qquad \textbf{(S2)}$$

, where N is the cumulative number of data points in current dataset, CNF_i, MMT_i, GEL_i, and GLY_i represent CNF loading, MMT loading, gelatin loading, and glycerol loading of one known data point (i) within the prediction model, and CNF_j, MMT_j, GEL_j, and GLY_j are the composition labels of one targeted data point (j) outside the prediction model.

On the other hand, $\hat{\sigma}$ denotes the variance of predicted property labels from the ANN committee, which is defined in **Equation S3**,

$$\hat{\sigma} = \sqrt[2]{\frac{1}{M}\sum_{j=1}^{M}\left(\begin{bmatrix} Output_{T_{UV}}^{j} \\ Output_{T_{Vis}}^{j} \\ Output_{T_{IR}}^{j} \\ Output_{RR}^{j} \\ Output_{\epsilon_f}^{j} \\ Output_{\sigma_u}^{j} \\ Output_{E}^{j} \\ Output_{\alpha}^{j} \\ Output_{\beta}^{j} \end{bmatrix} - \begin{bmatrix} Output_{T_{UV}}^{Ave} \\ Output_{T_{Vis}}^{Ave} \\ Output_{T_{IR}}^{Ave} \\ Output_{RR}^{Ave} \\ Output_{\varepsilon_f}^{Ave} \\ Output_{\sigma_u}^{Ave} \\ Output_{E}^{Ave} \\ Output_{\alpha}^{Ave} \\ Output_{\beta}^{Ave} \end{bmatrix}\right)^2} \quad \textbf{(S3)}$$

, where M is the total ANN number in the committee ($M = 5$), $Output_{T_{UV}}^{j}$, $Output_{T_{Vis}}^{j}$, $Output_{T_{IR}}^{j}$, $Output_{RR}^{j}$, $Output_{\varepsilon_f}^{j}$, $Output_{\sigma_u}^{j}$, $Output_{E}^{j}$, $Output_{\alpha}^{j}$, and $Output_{\beta}^{j}$ are the output property labels predicted by the jth decision program on basis of the composition labels of a targeted data point, $Output_{T_{UV}}^{Ave}$, $Output_{T_{Vis}}^{Ave}$, $Output_{T_{IR}}^{Ave}$, $Output_{RR}^{Ave}$, $Output_{\varepsilon_f}^{Ave}$, $Output_{\sigma_u}^{Ave}$, $Output_{E}^{Ave}$, $Output_{\alpha}^{Ave}$, and $Output_{\beta}^{Ave}$ are the average property labels predicted by the ANN committee on basis of the composition labels of a targeted data point. The open source code to implement *A Score*-based active learning loops in Python is provided in **GitHub** (https://github.com/chentl/MatAL/blob/master/active_leaerning.ipynb).

Note S8. Comparative studies of active learning sampling method.

Fig. S13 compares the active learning sampling with other methods, including random sampling, distance sampling, Latin hypercube sampling, and human intelligence sampling (done by a Ph.D. student). As shown in the distribution profiles of collected data points (**Fig. S13a–e**), a total of 5 sampling cycles were performed for each method, and 10 physical experiments were conducted in each cycle. As shown in **Fig. S14a** and **Table S6**, the active learning sampling recommended the MMT/CNF/gelatin/glycerol ratios with a >95% successful rate in producing *A*-grade nanocomposites. In contrast, other sampling methods exhibited much lower successful rates <60%. Subsequently, the UIP method was conducted at an 1-to-1,000 ratio, and the data points from different sampling methods were input to train multiple ANN models. Trained by the data points from the active learning sampling, the ANN model demonstrated higher learning efficiency and the lowest MRE values, as evidenced in **Fig. S14b**.

Note S9. Estimated time for the fabrication and characterization processes for all-natural nanocomposites.

Prior to the construction of the SVM classifier and ANN-based prediction model, about 2.0 liters of MMT dispersion (~8 mg mL^{-1}), CNF dispersion (~10 mg mL^{-1}), gelatin solution (~10 mg mL^{-1}), glycerol solution (~10 mg mL^{-1}) were required to be prepared in advance.

- Preparation of MMT dispersion: 3 days.
- Preparation of CNF dispersion: 4 days.
- Preparation of gelatin solution: 6 hours.
- Preparation of glycerol solution: 3 hours.

For the construction of the SVM classifier, 286 all-natural nanocomposites were fabricated, graded, and characterized in about 1 weeks.

- OT-2 robot-assisted mixture preparation: 6 hours.
- Air drying: 24 hours.
- Visual grading: 1 day.
- Detachability and flatness characterizations: 3 days.

For the construction of an ANN-based prediction model, one loop of active learning and data augmentation took approximately 2.5 days on average, and a total of 14 loops were conducted.

- OT-2 robot-assisted mixture preparation: 3 hours.
- Air drying: 24 hours.
- Transmittance spectrum measurements: 5 minutes for each of the three replicate samples.

- Fire resistance measurements: 10 minutes for each of the three replicate samples.
- Tensile tests: 15 minutes for each of the three replicate samples.
- Data analysis and model training: 8 hours.

Note S10. Working mechanism of clustering analysis.

In this work, we use the DBSCAN algorithm to search the clusters with high σ_u values in the feasible design space. The acronym stands for "Density-based Spatial Clustering of Applications with Noise". The central component to the DBSCAN algorithm is the concept of "core samples", which are the samples in the high-density areas. There are two crucial parameters to the DBSCAN algorithm, (1) $min\_samples$ and (2) eps. Higher $min\_samples$ or lower eps indicate higher density necessary to form a cluster. In this work, we set the $min\_samples$ and eps values to be 20 and 0.15, respectively. The *eps* parameter was chosen appropriately, which was used to control the local neighborhood of the data points. When chosen too small, most data points will not be clustered at all. When chosen too large, it causes close clusters to be merged into one cluster, and eventually the entire data set to be returned as a single cluster. The open source code to implement clustering analyses in Python is provided in **GitHub** (https://github.com/chentl/MatAL/blob/master/reverse_design.ipynb). It should be noted that before the model is employed to identify any champion samples through clustering analysis, the MRE (using an independent testing data points) needs to be sufficiently low. Afterward, a certain amount of experimental validation can be conducted near the model-suggested clusters with global maximum. Through both approaches, one can gain more confidence in the discovery of functional materials with superior properties.

Note S11. Two-step treatments of all-natural nanocomposites.

To further strengthen the MMT-rich and CNF-rich nanocomposites, two-step treatments were conducted, including (*1*) ionic crosslinking using divalent cations (i.e., Ca^{2+}) and (*2*) heat pressing (at 80 °C under 40 MPa). As shown in the cross-sectional SEM images (the inset of **Fig. 3h** and **3i**) and the Fourier transform infrared (FTIR) spectra (**Fig. S20**), both MMT-rich and CNF-rich nanocomposites were largely densified after the two-step treatments, with more hydrogen bonds induced. After the two-step treatments, the average σ_u values were significantly improved to 468.6 ± 52.6 MPa (from 7 densified MMT-rich nanocomposites, with the highest σ_u of 520.7 MPa) and 463.0 ± 35.7 MPa (from 9 densified CNF-rich nanocomposites, with the highest σ_u of 521.0 MPa). Additionally, the densified MMT-rich nanocomposites showed great fire resistance (with the RR of 0.99, **Fig. S21a**), while the densified CNF-rich nanocomposites showed high visible-light transmittance (with the T_{Vis} of 89.9%, **Fig. S21b**). However, it is important to note that the prediction model was not trained with the data points in the DOFs of ionic crosslinking and heat pressing, it is highly possible that the MMT-rich and CNF-rich ones did not exhibit the highest σ_u after two-step treatments. The primary reason for not training the prediction model with the data points in the DOFs of crosslinking and heat pressing was the extended processing time involved. Normally, the ionic crosslinking reactions typically lasted 12 hours, followed by additional 8 hours for heat pressing. The extended processing time considerably slowed our data acquisition rate during active learning loops.

Note S12. Perpectives on model expansion method.

Through strategic selections of new components or structural/physical parameters combined with the model expansion method, the prediction model continually expanded its design space and broadened the range of achievable functions. However, expanding the model necessitates more active loops, leading to higher time and cost implications. As such, creating autonomous fabrication and characterization platforms could enhance data collection efficiency, and establishing an open database for the community would also be a worthwhile endeavor.

Note S13. Biocompatibility of all-natural plastic substitutes.

To examine the biocompatibility of all-natural substitutes, multiple cytotoxicity experiments were conducted on L929 cells. These experiments included lactate dehydrogenase (LDH) assays, 3-(4,5-dimethylthiazol-2-yl)-2,5-diphenyltetrazolium bromide (MTT) assays, and live/dead cell assays. For the LDH and MTT assays (**Fig. S25a,b**), six all-natural substitutes were immersed in culture media overnight. Then, the media were extracted to culture L929 cells, and clean culture medium and triton X-100 were used as the negative and positive controls, respectively. All of the all-natural substitute extracts exhibited similar LDH and MTT levels as clean culture medium. For the live/dead cell assays (**Fig. S25c**), >95% of live cells were observed using all-natural substitute extracts, indicating that no cytotoxic substances were released to affect cell viability and survival.

Note S14. Water stability levels of various all-natural nanocomposites.

We executed the water stability tests for 132 kinds of all-natural nanocomposites spanning the entire feasible design space. The water stability tests involved immersing the all-natural nanocomposites in 1 vol.% of acetic acid solution and subjecting them to continuous shaking for 2 days. Afterwards, the shape retention conditions of 126 all-natural nanocomposites were evaluated. As shown in **Fig. S27a**, only when the all-natural nanocomposites stayed intact and preserved their original shapes, their water stability was categorized to the "intact" level. On the other hand, once the samples were fractured, partially or completely dissolved, their water stability was categorized to be the "dissolvable" level. **Table S1** and **Fig. S27b** summarize the water stability data of 132 all-natural nanocomposites with different MMT/CNF/gelatin/glycerol ratios. Then, these water stability data were input to train a SVM classifier, which generated a heatmap indicating the possibility of producing an all-natural nanocomposite with high water stability (**Fig. S27c**). This SVM model could be useful for tailoring the design of more water-stable, all-natural nanocomposites or developing the ones degrading more readily in water.

Note S15. Spearman's rank correlation coefficients.

To improve model's interpretability, multiple data analysis methods were implemented on over 150 data collected during active learning loops. Spearman's rank correlation coefficients (abbreviated as Spearman's ρ) were used to statistically assess the non-linear correlations between composition and property labels.[7] Meanwhile, p value was calculated to evaluate whether a significant correlation existed. A strong correlation can be confirmed if there is a high absolute value of Spearman's ρ (|Spearman's ρ|) and p value $\leq 10^{-2}$. For the spectral labels (T_{UV}, T_{Vis}, T_{IR}) (**Fig. S28a**), the CNF loading exhibited positive and strong correlations, while the MMT loading exhibited negative and strong correlations, as more micro-sized platelets induced light scattering. For the RR label (**Fig. S28b**), a positive and strong correlation was found with the MMT loading, which retarded oxygen permeation and thus decreased flammability.[8,9] For the mechanical labels (**Fig. S28c**), the CNF loading was positively correlated with ε_f, cohering with the literature.[10]

Note S16. Shapley Additive exPlanations (SHAP).

The SHAP analysis is a game theoretic approach to explain the output of any ML model (including the ensemble models). Basically, SHAP contains a permutation explainer program, and it works by iterating over complete permutations of the features. By continuing to conduct the iterations, the SHAP values are then calculated to approximate the contribution of each component loading to a specific property. It can find the feature importance inside the ML models, thus enabling the users to interpret the models. The process to find the feature importance is similar to finding the contribution of each player in a collaborative game. To understand how the feature importance is found in SHAP, an example is presented below.

As shown in **Fig. S29a**, there are three players A, B, and C. They collaborate with each other to play a game. When all of them join the game, based on their different skills in a specific game (e.g., 8, 3 and 3 for players A, B, and C.), they can achieve a 100 reward. The task is to quantify how important each player is in getting the reward.

To solve this, we assume that the group members join in a specific sequence (e.g., player A first, then player B, next player C) and the marginal rewards of each player is recorded. For example, player A is the first member with a reward of 50, then player B joins the game and brings the reward to 90, and next player C joins to bring the reward to 100. Therefore, the players' respective marginal rewards are "player A, B, C = 50, 40, 10". However, the calculated marginal reward may not accurately represent the contribution of each player. For example, when the sequence is changed from player A, B, C to player A, C, B, if players B and C have a similar skill set, the rewards are still 50, 40, and 10 after player A, C, B joins the game, respectively. Then the players' respective marginal rewards are changed to "player A, B, C = 50, 10, 40". Therefore, the sequence of how the players join the game is important.

In order to get a more accurate reward of each individual player, we need to find out the marginal reward of each player under every possible sequence. The reward for each individual player then can be the sum of these marginal rewards over the number of possible sequences (the calculation for a specific player is illustrated in **Equation S4**). For example, in the case outlined above, we can simulate the arrival sequences: ABC, ACB, BCA, BAC, CAB, and CBA, and the marginal reward of each player is recorded for each sequence. Then, by averaging all of these rewards, we obtain the reward contributed from each player. This reward is the SHAP value.

$$\text{SHAP value of a player} = \frac{Sum\ of\ mariginal\ rewards\ of\ a\ specific\ player\ under\ all\ possible\ sequences}{number\ of\ total\ possible\ sequences}$$

(S4)

Back to the feature importance analysis of our developed prediction model, we can take the problem as an analogy to the above case. All composition labels (CNF loading, MMT loading, gelatin loading, and glycerol loading) are regarded as players, which are fed into the prediction model to obtain the property labels. The prediction process is treated as the game, and the deviation (between the predicted property label of a specific data point and the average property label from all data points) is treated as the reward. By following **Equation S6**, the SHAP value of each composition label on a specific property label can be calculated, and this value is used to measure the feature importance.

$$\text{SHAP value of a composition label} = \frac{Sum\ of\ mariginal\ reward\ of\ a\ compoisiton\ label\ on\ a\ specific\ property\ label\ under\ all\ possible\ sequences}{Number\ of\ total\ possible\ sequences}$$

(S5)

The above process is the interpretation of the prediction model on a specific data point which is called the local interpretation. To get the global interpretation of the prediction model

over all data points, we can plot the SHAP values of every composition label for every data point, as shown in **Fig. S29b**. A wider range of the SHAP value for a specific feature indicates a higher importance, and *vice versa*. The open source code to implement SHAP analyses in Python is provided in **GitHub** (https://github.com/chentl/MatAL/blob/master/plot_shaps.ipynb).

Note S17. Comparison of Young's moduli of CNF only and MMT only models.

In both our experimental and simulation results, we indeed observed that the Young's moduli of MMT only samples were lower than those of CNF only samples. These trends can be elucidated as follows. Crystalline MMT exhibits an exceptionally high Young's modulus of 178 GPa, which is notably higher than that of most biopolymers.[11] However, natural MMT exhibits a range of Young's moduli, varying from 6 to 63 GPa, due to the presence of defects and weak interlayer interactions.[12] In this study, the MMT only thin films were fabricated by initially exfoliating natural MMT crystals, a process during which the interfacial ions were dissolved in the solvent (e.g., water)[13]. Following the production of MMT nanosheets, we assembled them into a multilayer configuration. Consequently, the self-assembled MMT multilayers exhibited a significant reduction in their Young's moduli, primarily attributing to the presence of defects and the absence of interfacial ions. To simulate the thin-film preparation conditions, we constructed the MMT only model with partial removal of interfacial ions between MMT layers, resulting in a lower Young's modulus of approximately 1.4 GPa. In contrast, the CNF only model contained cellulose chains rich in hydroxyl groups, which facilitated the formation of hydrogen bonds between neighboring CNF units, leading to strong interlayer interactions. Similar findings are supported by the literature. For instance, bacterial cellulose films have been reported to achieve a high Young's modulus of 48.1 GPa.[14] Additionally, anisotropic CNF only films exhibited a high Young's modulus of 57.8 GPa, which is nearly 55 times higher than that of isotropic paper (1.1 GPa).[15] In summary, the Young's moduli of self-assembled MMT multilayers could be lower than that of crystalline MMT and comparable to CNF only thin films.

The substantial difference in stiffness can be understood as follows. The stiffness of CNF only and MMT only samples is largely dictated by the initial elastic response of the inter-fiber

interactions of CNFs and the inter-particle interactions of MMT nanosheets. In contrast, due to the electronegativity of the MMT surface, the polar groups on the CNF chains can be bound tightly to MMT nanosheets, leading to strong interactions at the CNF/MMT interfaces. Such strong CNF/MMT bonding effectively constrains the inter-fiber deformation in CNFs and the inter-particle deformation in MMT nanosheets upon tension. Instead, the elongation of the MMT/CNF model is accommodated by stretching individual CNF fiber and MMT particle, which lead to a stiffness significantly higher than the CNF only and MMT only models.

Note S18. Potential approaches to evaluating the structural and physical attributes of building blocks.

Molecular string representations. Utilizing molecular string representations (like SMILES, SELFIES, or mol2vec) allows for capturing the complex chemical structure of each building block, including bonds, functional groups, chirality, and more.[16,17] In our preliminary studies, glycerol and gelatin can be denoted using SMILES as "C(C(CO)O)O" and "[H][C@@]1(C[C@@](C)(OC)[C@@H](O)[C@H](C)O1)O[C@H]1[C@H](C)[C@@H](O[C@]2([H])O[C@H](C)C[C@@H]([C@H]2O)N(C)C)[C@](C)(O)C[C@@H](C)C(=O)[C@H](C)[C@@H](O)[C@](C)(O)[C@@H](CC)OC(=O)[C@@H]1C", respectively. However, significant challenges exist in describing gelatin from various sources and representing structural nanomaterials (such as MMT nanosheets or CNFs). To the best of our knowledge, it remains limited to describe the nanoscale interactions between building blocks using molecular string representations.

MD simulations. MD simulations provide valuable insights into the intricate molecular interactions among various building blocks. By incorporating the MD simulation results into the prediction model, we can assess both the molecular interactions between building blocks and their structural and physical influences. In our preliminary studies, we constructed two distinct MMT/CNF models: one comprising CNF chains at 80% of their original lengths (**Fig. S35a**) and another with MMT nanosheets at 60% of their original sizes (**Fig. S35b**). As shown in **Fig. S35c**, we simulated the stress–strain curves for these two MMT/CNF models. Both models, with shorter CNF chains and smaller MMT nanosheets, exhibited much reduced mechanical resilience, which aligns well with the experimental evidence shown in **Fig. S33e**. These initial studies showcased the potential of using MD simulations to incorporate the structural and physical influences of

building blocks into the prediction model. However, running MD simulations for the systems that include three or more building blocks at various ratios requires considerable computational resources, which could hinder the model's learning efficiency.

Movie S1. Automated pipetting robot (i.e., OT-2 robot) for preparing various MMT/CNF/gelatin/glycerol mixtures.

Movie S2. Deformation and tensile failure processes of the CNF only model.

Movie S3. Deformation and tensile failure processes of the MMT only model.

Movie S4. Deformation and tensile failure processes of the CNF/MMT model.

Table S1. Grades of 286 nanocomposites with different MMT/CNF/gelatin/glycerol ratios. For column "Grade": "*A*-grades" refer to the conditions that the nanocomposite films were detachable and flat. "*B*-grades" refer to the conditions that the nanocomposite films were detachable yet curved. "*C*-grades" refer to the conditions that the nanocomposite films were detachable yet fractured. "*D*-grades" refer to the conditions that the nanocomposite films were non-detachable. For column "Water Stability": "I" refers to the "intact" cases. "S" refers to the "dissolvable" cases.

ID	MMT/CNF/Gelatin/Glycerol Ratio				Grade	Water Stability
	MMT	CNF	Gelatin	Glycerol		
1	0.0	0.0	0.6	0.4	D	–
2	0.0	0.0	1.0	0.0	A	I
3	0.0	0.2	0.8	0.0	A	S
4	0.0	0.2	0.6	0.2	A	S
5	0.0	0.2	0.4	0.4	B	–
6	0.0	0.4	0.2	0.4	B	–
7	0.0	0.4	0.4	0.2	A	S
8	0.0	0.4	0.6	0.0	A	S
9	0.0	0.6	0.0	0.4	B	–
10	0.0	0.6	0.2	0.2	B	–
11	0.0	0.6	0.4	0.0	A	S
12	0.0	0.8	0.0	0.2	B	–
13	0.0	0.8	0.2	0.0	B	–
14	0.0	1.0	0.0	0.0	B	–
15	0.2	0.0	0.4	0.4	D	–
16	0.2	0.0	0.6	0.2	D	–
17	0.2	0.0	0.8	0.0	C	–
18	0.2	0.2	0.2	0.4	C	–
19	0.2	0.2	0.4	0.2	D	–
20	0.2	0.2	0.6	0.0	C	–
21	0.2	0.4	0.0	0.4	C	–
22	0.2	0.4	0.2	0.2	A	S
23	0.2	0.4	0.4	0.0	A	S
24	0.2	0.6	0.0	0.2	C	–
25	0.2	0.6	0.2	0.0	B	–
26	0.2	0.8	0.0	0.0	B	–
27	0.4	0.0	0.2	0.4	A	I
28	0.4	0.0	0.4	0.2	A	I
29	0.4	0.0	0.6	0.0	C	–
30	0.4	0.2	0.0	0.4	A	I
31	0.4	0.2	0.2	0.2	A	I
32	0.4	0.2	0.4	0.0	A	S
33	0.4	0.4	0.0	0.2	B	–
34	0.4	0.4	0.2	0.0	B	–
35	0.4	0.6	0.0	0.0	B	–
36	0.6	0.0	0.0	0.4	C	–
37	0.6	0.0	0.2	0.2	B	–
38	0.6	0.0	0.4	0.0	A	I
39	0.6	0.2	0.0	0.2	B	–
40	0.6	0.2	0.2	0.0	A	I
41	0.6	0.4	0.0	0.0	B	–
42	0.8	0.0	0.0	0.2	C	–
43	0.8	0.0	0.2	0.0	A	I
44	0.8	0.2	0.0	0.0	C	–
45	1.0	0.0	0.0	0.0	C	–
46	0.0	0.0	0.8	0.2	A	S
47	0.2	0.7	0.1	0.0	B	–
48	0.3	0.0	0.3	0.4	A	I
49	0.3	0.0	0.4	0.3	D	–
50	0.3	0.0	0.5	0.2	C	–
51	0.3	0.0	0.6	0.1	A	I
52	0.3	0.0	0.7	0.0	A	I

ID	MMT/CNF/Gelatin/Glycerol Ratio				Grade	Water Stability
	MMT	CNF	Gelatin	Glycerol		
53	0.3	0.1	0.2	0.4	D	–
54	0.3	0.1	0.3	0.3	D	–
55	0.3	0.1	0.4	0.2	D	–
56	0.3	0.1	0.5	0.1	A	S
57	0.3	0.1	0.6	0.0	A	S
58	0.3	0.2	0.1	0.4	D	–
59	0.3	0.2	0.2	0.3	A	I
60	0.3	0.2	0.3	0.2	A	S
61	0.3	0.2	0.4	0.1	A	S
62	0.3	0.2	0.5	0.0	A	S
63	0.3	0.3	0.0	0.4	A	I
64	0.3	0.3	0.1	0.3	A	I
65	0.3	0.3	0.2	0.2	A	S
66	0.3	0.3	0.3	0.1	A	S
67	0.3	0.3	0.4	0.0	A	S
68	0.3	0.4	0.0	0.3	A	S
69	0.3	0.4	0.1	0.2	A	I
70	0.3	0.4	0.2	0.1	A	S
71	0.3	0.4	0.3	0.0	A	S
72	0.3	0.5	0.0	0.2	B	–
73	0.3	0.5	0.1	0.1	B	–
74	0.3	0.5	0.2	0.0	B	–
75	0.3	0.6	0.0	0.1	B	–
76	0.3	0.6	0.1	0.0	B	–
77	0.3	0.7	0.0	0.0	B	–
78	0.4	0.0	0.3	0.3	A	I
79	0.4	0.0	0.5	0.1	A	I
80	0.4	0.1	0.1	0.4	D	–
81	0.4	0.1	0.2	0.3	A	I
82	0.4	0.1	0.3	0.2	A	S
83	0.4	0.1	0.4	0.1	A	S
84	0.4	0.1	0.5	0.0	A	S
85	0.4	0.2	0.1	0.3	A	I
86	0.4	0.2	0.3	0.1	A	I
87	0.4	0.3	0.0	0.3	A	S
88	0.4	0.3	0.1	0.2	A	I
89	0.4	0.3	0.2	0.1	A	I
90	0.4	0.3	0.3	0.0	A	I

ID	MMT/CNF/Gelatin/Glycerol Ratio				Grade	Water Stability
	MMT	CNF	Gelatin	Glycerol		
91	0.4	0.4	0.1	0.1	A	S
92	0.4	0.5	0.0	0.1	C	–
93	0.4	0.5	0.1	0.0	B	–
94	0.5	0.0	0.1	0.4	A	I
95	0.5	0.0	0.2	0.3	A	I
96	0.5	0.0	0.3	0.2	A	I
97	0.5	0.0	0.4	0.1	C	–
98	0.5	0.0	0.5	0.0	A	S
99	0.5	0.1	0.0	0.4	D	–
100	0.5	0.1	0.1	0.3	A	I
101	0.5	0.1	0.2	0.2	A	I
102	0.5	0.1	0.3	0.1	C	–
103	0.5	0.1	0.4	0.0	C	–
104	0.5	0.2	0.0	0.3	C	–
105	0.5	0.2	0.1	0.2	A	S
106	0.5	0.2	0.2	0.1	A	S
107	0.5	0.2	0.3	0.0	A	I
108	0.5	0.3	0.0	0.2	A	I
109	0.5	0.3	0.1	0.1	A	I
110	0.5	0.3	0.2	0.0	A	I
111	0.5	0.4	0.0	0.1	C	–
112	0.5	0.4	0.1	0.0	A	S
113	0.5	0.5	0.0	0.0	B	–
114	0.6	0.0	0.1	0.3	A	S
115	0.6	0.0	0.3	0.1	A	I
116	0.6	0.1	0.0	0.3	C	–
117	0.6	0.1	0.1	0.2	A	I
118	0.6	0.1	0.2	0.1	A	S
119	0.6	0.1	0.3	0.0	A	S
120	0.6	0.2	0.1	0.1	A	I
121	0.6	0.3	0.0	0.1	A	S
122	0.6	0.3	0.1	0.0	A	S
123	0.7	0.0	0.0	0.3	D	–
124	0.7	0.0	0.1	0.2	C	–
125	0.7	0.0	0.2	0.1	A	I
126	0.7	0.0	0.3	0.0	A	I
127	0.7	0.1	0.0	0.2	A	I
128	0.7	0.1	0.1	0.1	A	I

ID	MMT/CNF/Gelatin/Glycerol Ratio				Grade	Water Stability
	MMT	CNF	Gelatin	Glycerol		
129	0.7	0.1	0.2	0.0	A	S
130	0.7	0.2	0.0	0.1	A	I
131	0.7	0.2	0.1	0.0	B	–
132	0.7	0.3	0.0	0.0	B	–
133	0.8	0.0	0.1	0.1	A	S
134	0.8	0.1	0.0	0.1	C	–
135	0.8	0.1	0.1	0.0	A	I
136	0.9	0.0	0.0	0.1	A	I
137	0.9	0.0	0.1	0.0	A	I
138	0.9	0.1	0.0	0.0	C	–
139	0.0	0.0	0.7	0.3	D	–
140	0.0	0.0	0.9	0.1	A	S
141	0.0	0.1	0.5	0.4	D	–
142	0.0	0.1	0.6	0.3	C	–
143	0.0	0.1	0.7	0.2	D	–
144	0.0	0.1	0.8	0.1	C	–
145	0.0	0.1	0.9	0.0	B	–
146	0.0	0.2	0.5	0.3	D	–
147	0.0	0.2	0.7	0.1	A	S
148	0.0	0.3	0.3	0.4	D	–
149	0.0	0.3	0.4	0.3	C	–
150	0.0	0.3	0.5	0.2	A	S
151	0.0	0.3	0.6	0.1	A	S
152	0.0	0.3	0.7	0.0	A	I
153	0.0	0.4	0.3	0.3	A	S
154	0.0	0.4	0.5	0.1	A	S
155	0.0	0.5	0.1	0.4	A	S
156	0.0	0.5	0.2	0.3	A	S
157	0.0	0.5	0.3	0.2	A	S
158	0.0	0.5	0.4	0.1	A	S
159	0.0	0.5	0.5	0.0	A	S
160	0.0	0.6	0.1	0.3	A	S
161	0.0	0.6	0.3	0.1	A	S
162	0.0	0.7	0.0	0.3	C	–
163	0.0	0.7	0.1	0.2	A	S
164	0.0	0.7	0.2	0.1	A	S
165	0.0	0.7	0.3	0.0	A	S
166	0.0	0.8	0.1	0.1	A	S

ID	MMT/CNF/Gelatin/Glycerol Ratio				Grade	Water Stability
	MMT	CNF	Gelatin	Glycerol		
167	0.0	0.9	0.0	0.1	B	–
168	0.0	0.9	0.1	0.0	A	S
169	0.1	0.0	0.5	0.4	D	–
170	0.1	0.0	0.6	0.3	D	–
171	0.1	0.0	0.7	0.2	D	–
172	0.1	0.0	0.8	0.1	D	–
173	0.1	0.0	0.9	0.0	C	–
174	0.1	0.1	0.4	0.4	D	–
175	0.1	0.1	0.5	0.3	D	–
176	0.1	0.1	0.6	0.2	D	–
177	0.1	0.1	0.7	0.1	C	–
178	0.1	0.1	0.8	0.0	D	–
179	0.1	0.2	0.3	0.4	D	–
180	0.1	0.2	0.4	0.3	C	–
181	0.1	0.2	0.5	0.2	A	I
182	0.1	0.2	0.6	0.1	C	–
183	0.1	0.2	0.7	0.0	C	–
184	0.1	0.3	0.2	0.4	D	–
185	0.1	0.3	0.3	0.3	A	S
186	0.1	0.3	0.4	0.2	A	S
187	0.1	0.3	0.5	0.1	A	I
188	0.1	0.3	0.6	0.0	D	–
189	0.1	0.4	0.2	0.3	D	–
190	0.1	0.4	0.3	0.2	A	S
191	0.1	0.4	0.4	0.1	A	S
192	0.1	0.4	0.5	0.0	D	–
193	0.1	0.5	0.0	0.4	A	S
194	0.1	0.5	0.1	0.3	A	I
195	0.1	0.5	0.2	0.2	A	S
196	0.1	0.5	0.3	0.1	A	S
197	0.1	0.5	0.4	0.0	D	–
198	0.1	0.6	0.0	0.3	A	S
199	0.1	0.6	0.1	0.2	A	S
200	0.1	0.6	0.2	0.1	A	S
201	0.1	0.6	0.3	0.0	A	S
202	0.1	0.7	0.0	0.2	B	–
203	0.1	0.7	0.1	0.1	B	–
204	0.1	0.7	0.2	0.0	A	S

ID	MMT/CNF/Gelatin/Glycerol Ratio				Grade	Water Stability
	MMT	CNF	Gelatin	Glycerol		
205	0.1	0.8	0.0	0.1	B	–
206	0.1	0.8	0.1	0.0	B	–
207	0.1	0.9	0.0	0.0	B	–
208	0.2	0.0	0.5	0.3	D	–
209	0.2	0.0	0.7	0.1	C	–
210	0.2	0.1	0.3	0.4	D	–
211	0.2	0.1	0.4	0.3	D	–
212	0.2	0.1	0.5	0.2	D	–
213	0.2	0.1	0.6	0.1	C	–
214	0.2	0.1	0.7	0.0	C	–
215	0.2	0.2	0.3	0.3	D	–
216	0.2	0.2	0.5	0.1	A	I
217	0.2	0.3	0.1	0.4	D	–
218	0.1	0.4	0.1	0.4	D	–
219	0.2	0.3	0.2	0.3	D	–
220	0.2	0.3	0.3	0.2	A	S
221	0.2	0.3	0.4	0.1	A	I
222	0.2	0.3	0.5	0.0	C	–
223	0.2	0.4	0.1	0.3	A	S
224	0.2	0.4	0.3	0.1	A	S
225	0.2	0.5	0.0	0.3	A	S
226	0.2	0.5	0.1	0.2	A	S
227	0.2	0.5	0.2	0.1	B	–
228	0.2	0.5	0.3	0.0	A	S
229	0.2	0.6	0.1	0.1	B	–
230	0.2	0.7	0.0	0.1	B	–
231	0.0	0.0	0.0	1.0	D	–
232	0.0	0.0	0.3	0.7	D	–
233	0.0	0.0	0.4	0.6	D	–
234	0.0	0.0	0.5	0.5	D	–
235	0.0	0.1	0.0	0.9	D	–
236	0.0	0.1	0.1	0.8	C	–
237	0.0	0.1	0.2	0.7	D	–
238	0.0	0.1	0.3	0.6	D	–
239	0.0	0.1	0.4	0.5	D	–
240	0.0	0.2	0.0	0.8	C	–
241	0.0	0.2	0.1	0.7	C	–
242	0.0	0.2	0.2	0.6	C	–

ID	MMT/CNF/Gelatin/Glycerol Ratio				Grade	Water Stability
	MMT	CNF	Gelatin	Glycerol		
243	0.0	0.2	0.3	0.5	C	–
244	0.0	0.3	0.0	0.7	A	S
245	0.0	0.3	0.1	0.6	A	S
246	0.0	0.3	0.2	0.5	A	S
247	0.0	0.4	0.0	0.6	A	S
248	0.0	0.4	0.1	0.5	A	S
249	0.0	0.5	0.0	0.5	A	S
250	0.1	0.0	0.0	0.9	D	–
251	0.1	0.0	0.1	0.8	D	–
252	0.1	0.0	0.2	0.7	D	–
253	0.1	0.0	0.3	0.6	D	–
254	0.1	0.0	0.4	0.5	D	–
255	0.1	0.1	0.0	0.8	D	–
256	0.1	0.1	0.1	0.7	D	–
257	0.1	0.1	0.2	0.6	C	–
258	0.1	0.1	0.3	0.5	C	–
259	0.1	0.2	0.0	0.7	C	–
260	0.1	0.2	0.1	0.6	D	–
261	0.1	0.2	0.2	0.5	C	–
262	0.1	0.3	0.0	0.6	A	S
263	0.1	0.3	0.1	0.5	A	S
264	0.1	0.4	0.0	0.5	A	S
265	0.2	0.0	0.0	0.8	D	–
266	0.2	0.0	0.1	0.7	D	–
267	0.2	0.0	0.2	0.6	D	–
268	0.2	0.0	0.3	0.5	D	–
269	0.2	0.1	0.0	0.7	D	–
270	0.2	0.1	0.1	0.6	D	–
271	0.2	0.1	0.2	0.5	D	–
272	0.2	0.2	0.0	0.6	C	–
273	0.2	0.2	0.1	0.5	D	–
274	0.2	0.3	0.0	0.5	A	I
275	0.3	0.0	0.0	0.7	D	–
276	0.3	0.0	0.1	0.6	D	–
277	0.3	0.0	0.2	0.5	D	–
278	0.3	0.1	0.0	0.6	C	–
279	0.3	0.1	0.1	0.5	D	–
280	0.3	0.2	0.0	0.5	A	I

ID	MMT/CNF/Gelatin/Glycerol Ratio				Grade	Water Stability
	MMT	CNF	Gelatin	Glycerol		
281	0.4	0.0	0.0	0.6	D	–
282	0.4	0.0	0.1	0.5	D	–
283	0.4	0.1	0.0	0.5	C	–
284	0.5	0.0	0.0	0.5	D	–
285	0.0	0.0	0.1	0.9	D	–
286	0.0	0.0	0.2	0.8	D	–

Table S2: Photos of 286 nanocomposites with different MMT/CNF/gelatin/glycerol ratios.

1	2	3	4	5
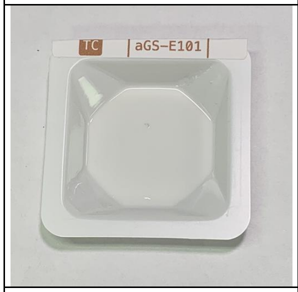	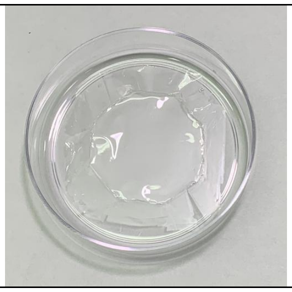	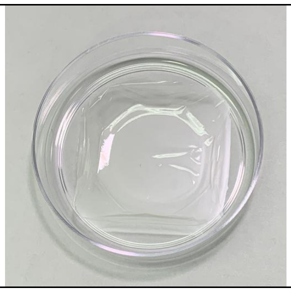	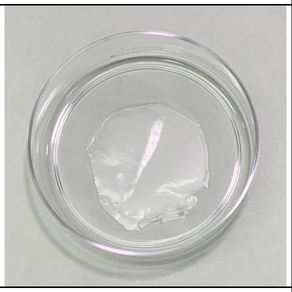	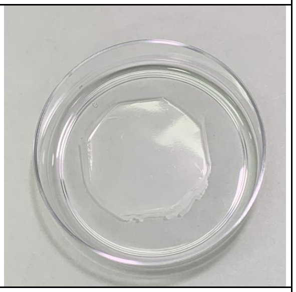
6	7	8	9	10
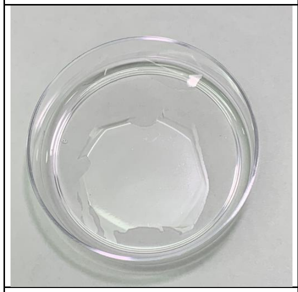	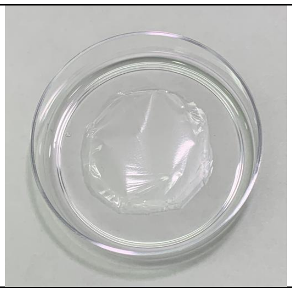	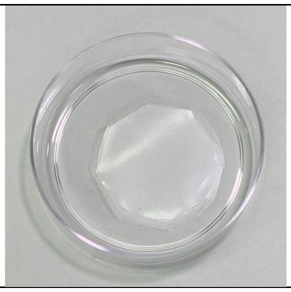	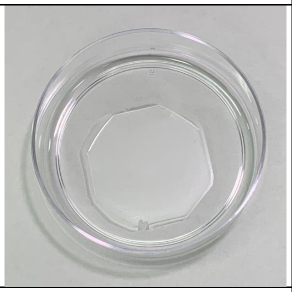	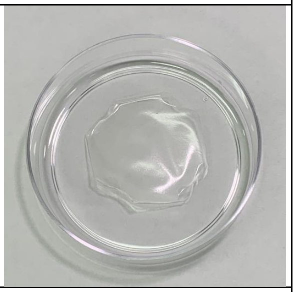
11	12	13	14	15
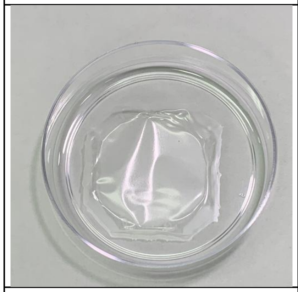	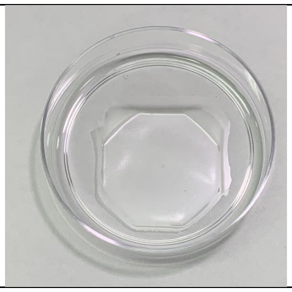	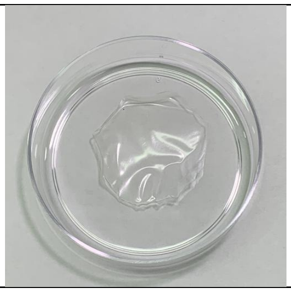	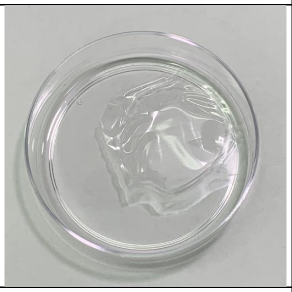	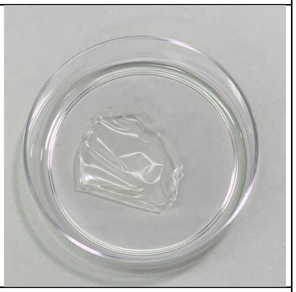
16	17	18	19	20
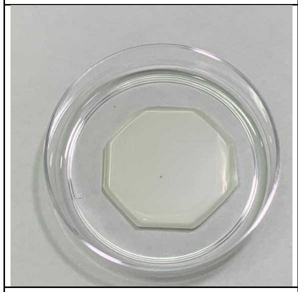	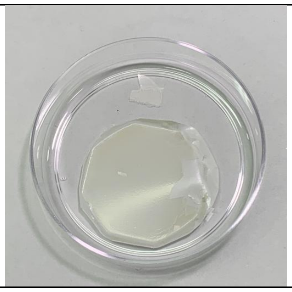	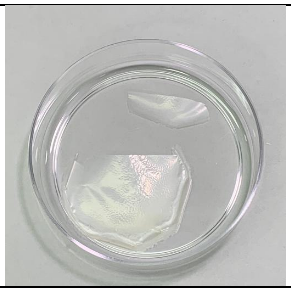	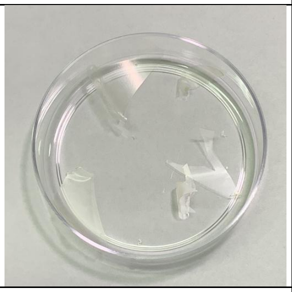	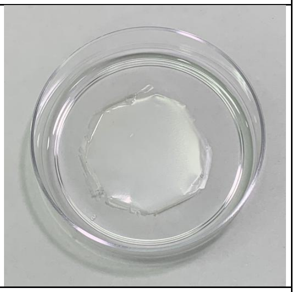
21	22	23	24	25
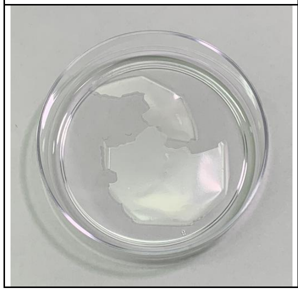	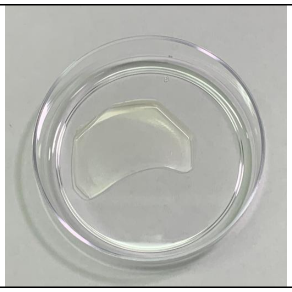	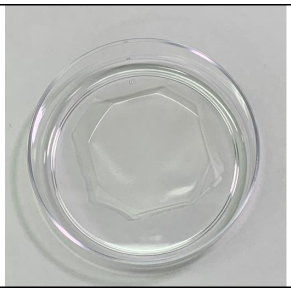	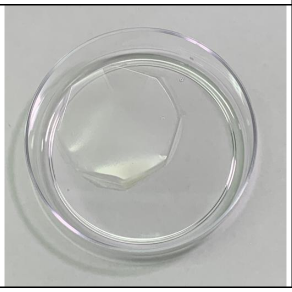	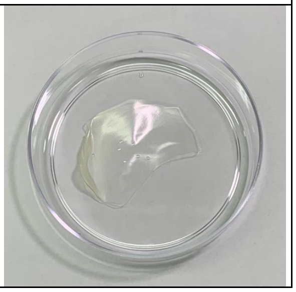

26	27	28	29	30
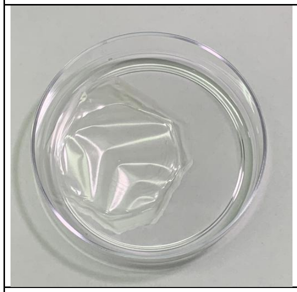	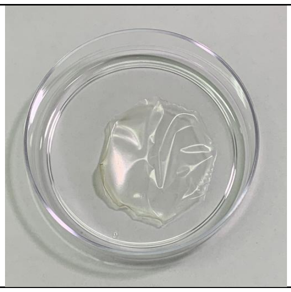	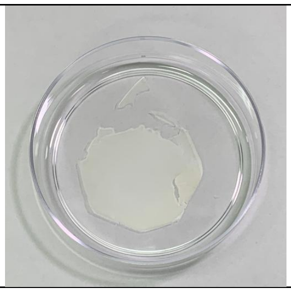	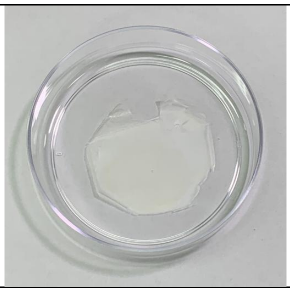	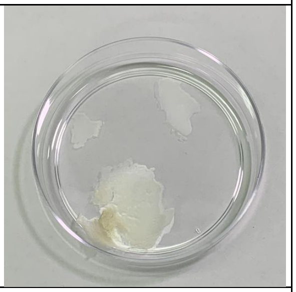
31	32	33	34	35
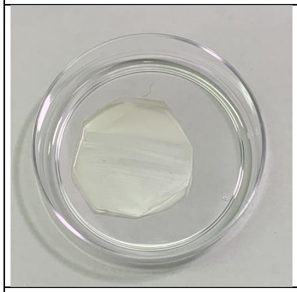	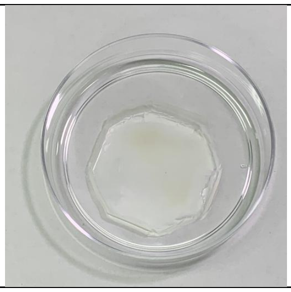	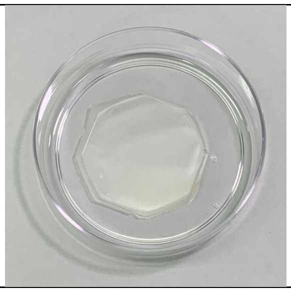	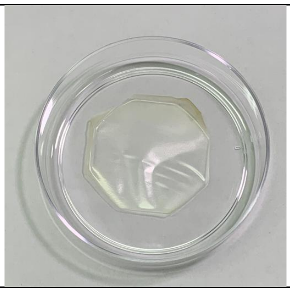	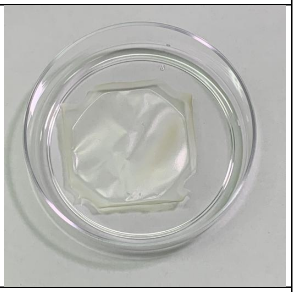
36	37	38	39	40
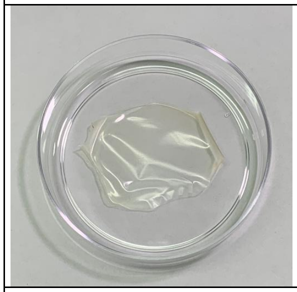	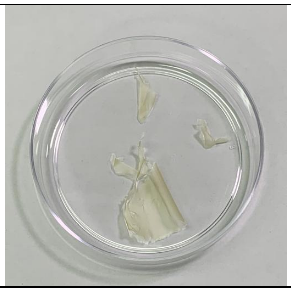	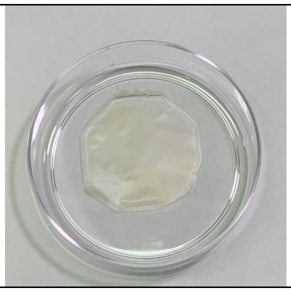	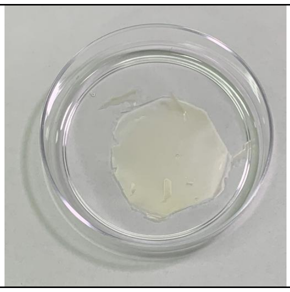	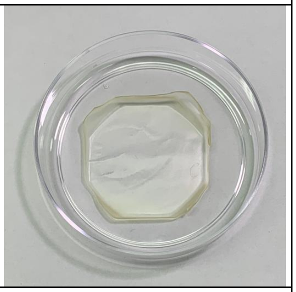
41	42	43	44	45
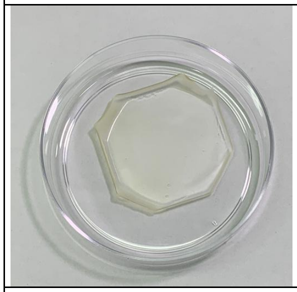	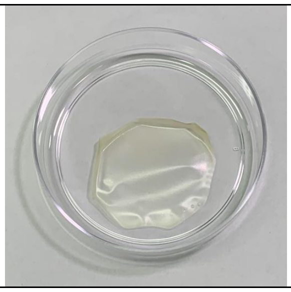	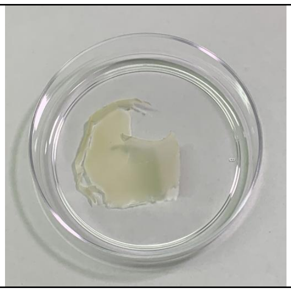	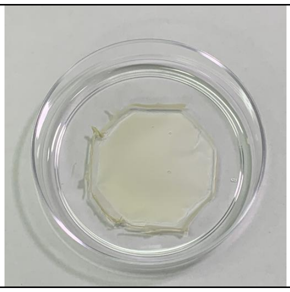	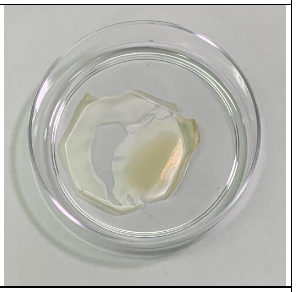
46	47	48	49	50
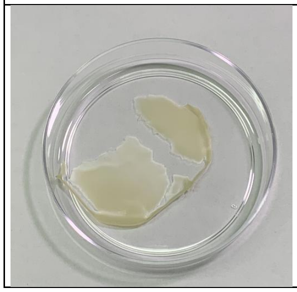	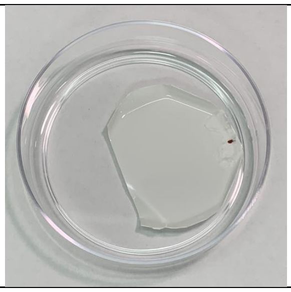	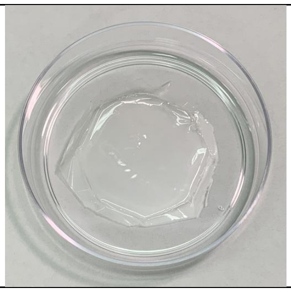	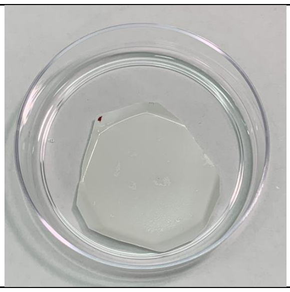	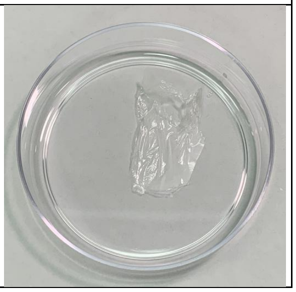

51	52	53	54	55
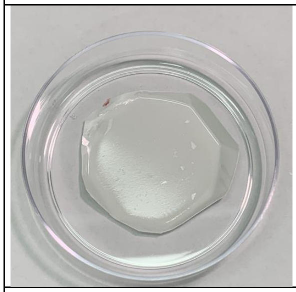	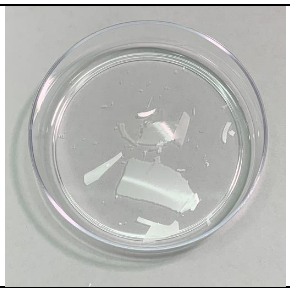	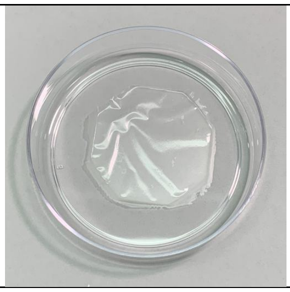	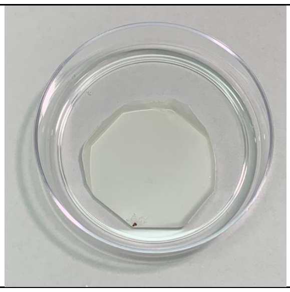	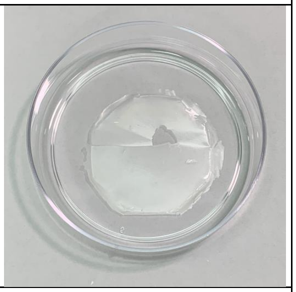
56	57	58	59	60
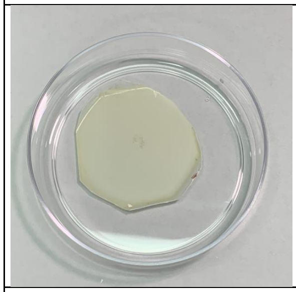	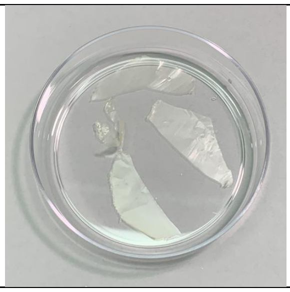	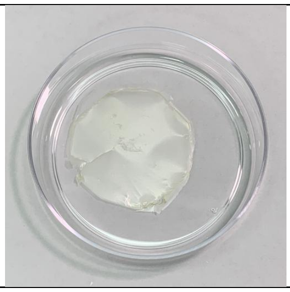	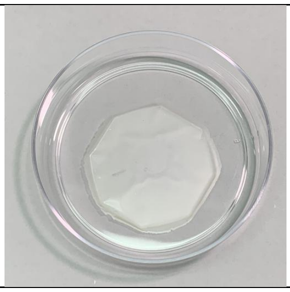	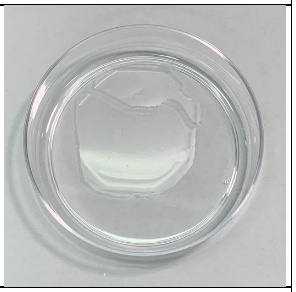
61	62	63	64	65
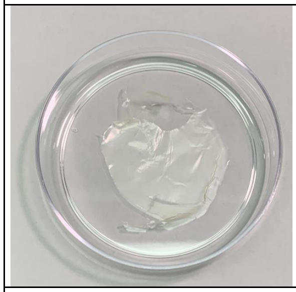	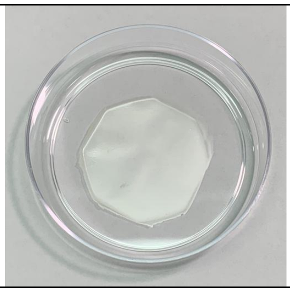	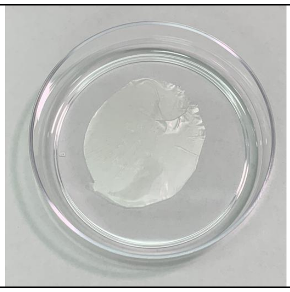	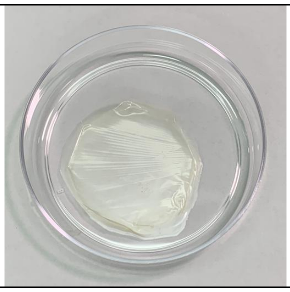	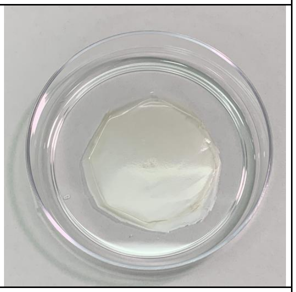
66	67	68	69	70
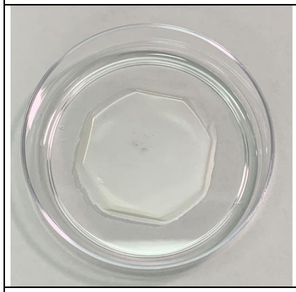	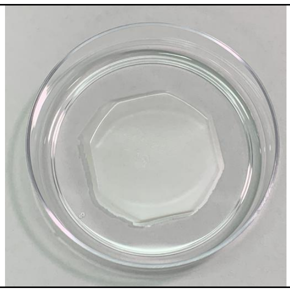	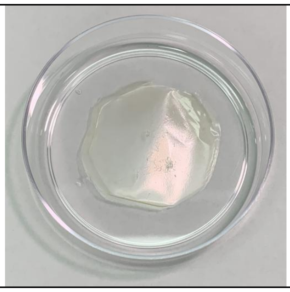	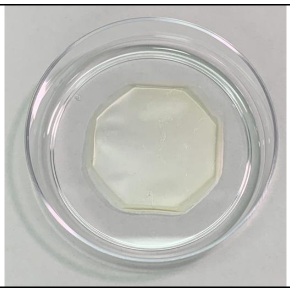	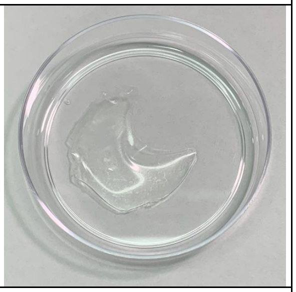
71	72	73	74	75
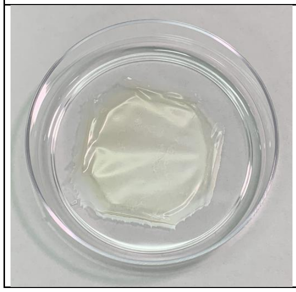	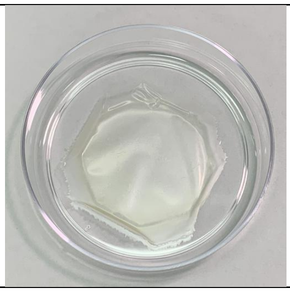	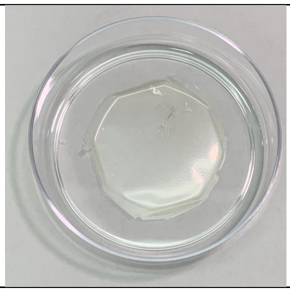	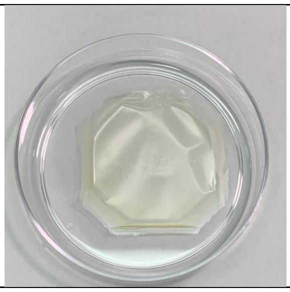	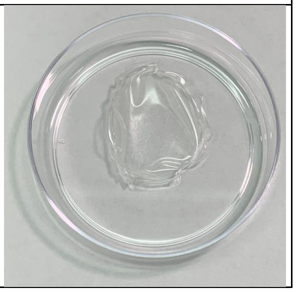

76	77	78	79	80
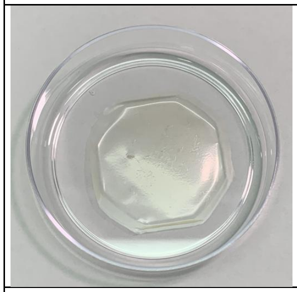	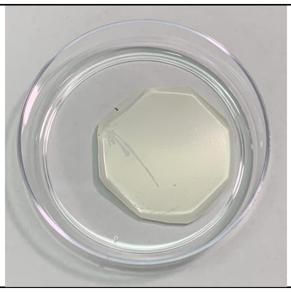	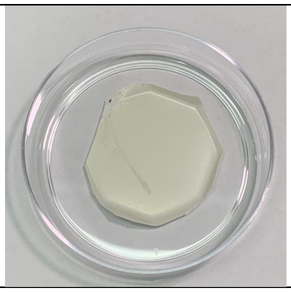	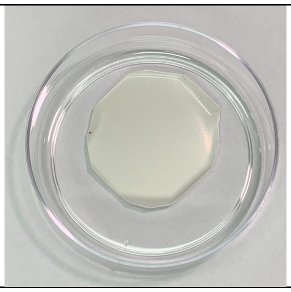	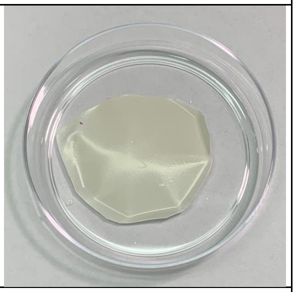
81	82	83	84	85
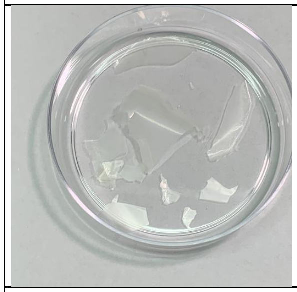	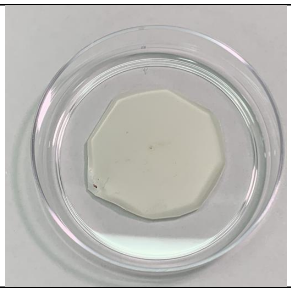	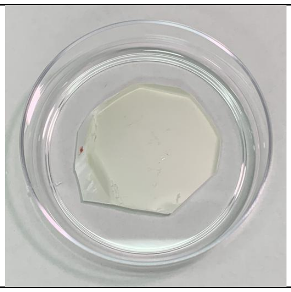	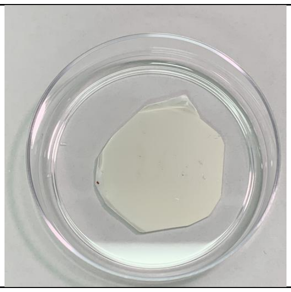	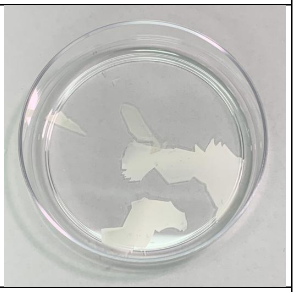
86	87	88	89	90
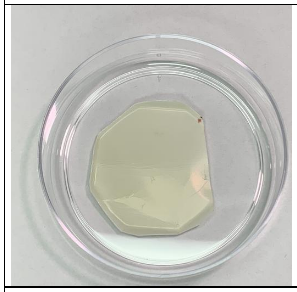	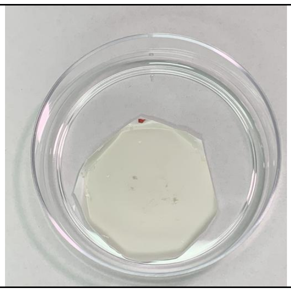	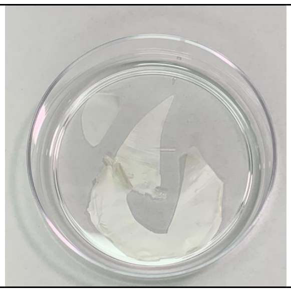	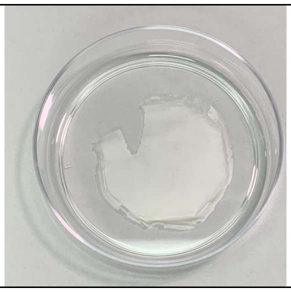	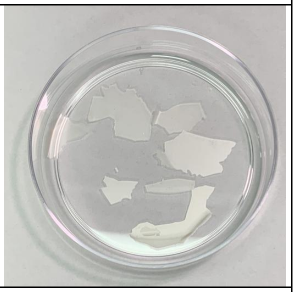
91	92	93	94	95
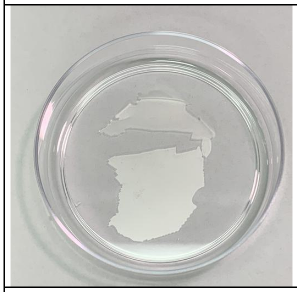	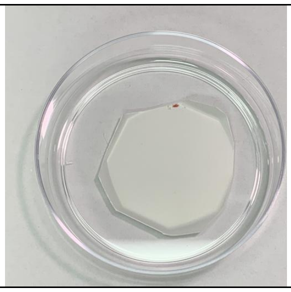	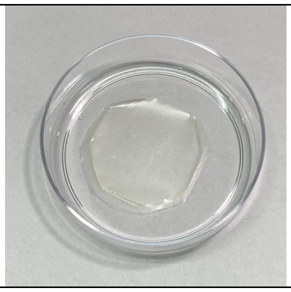	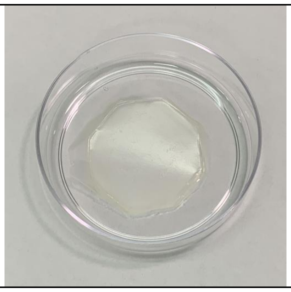	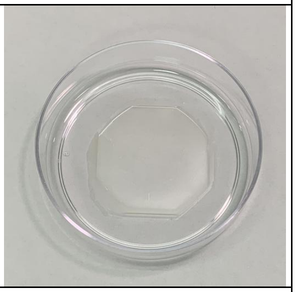
96	97	98	99	100
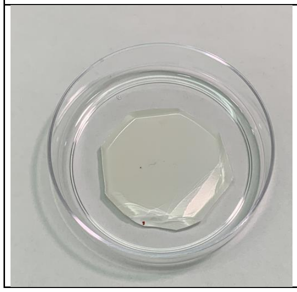	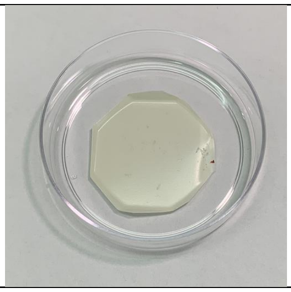	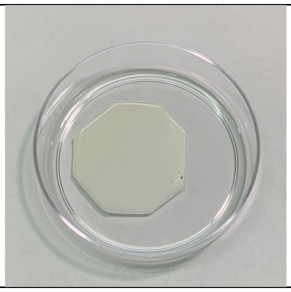	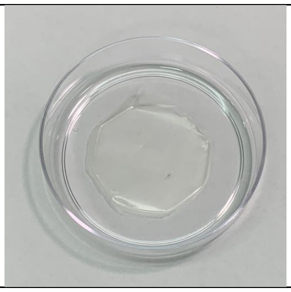	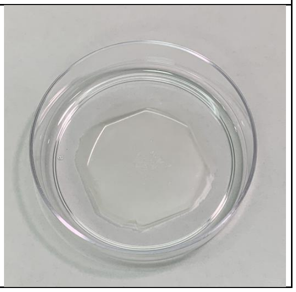

101	102	103	104	105
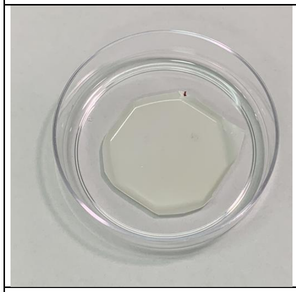	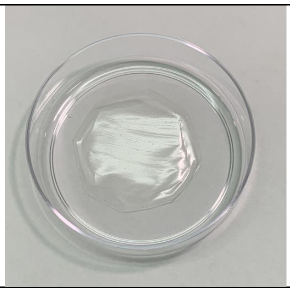	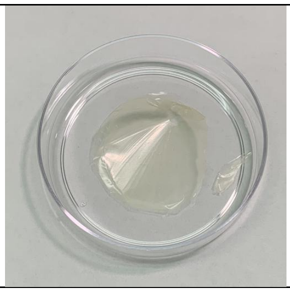	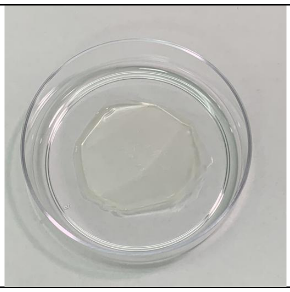	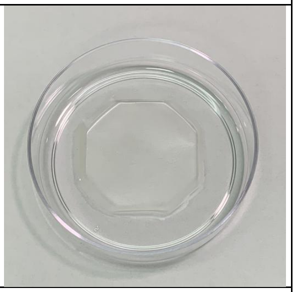
106	107	108	109	110
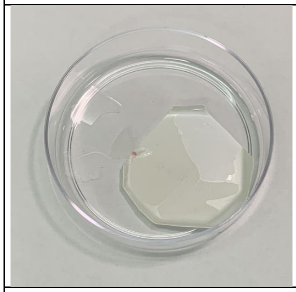	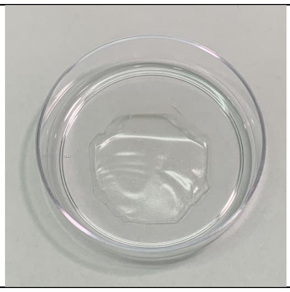	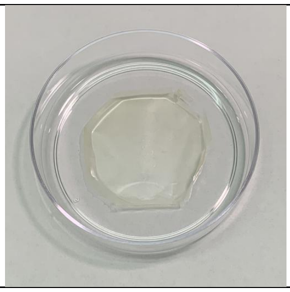	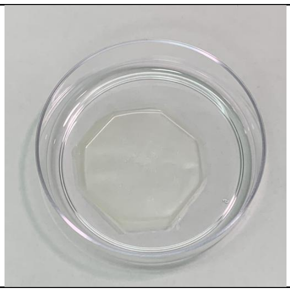	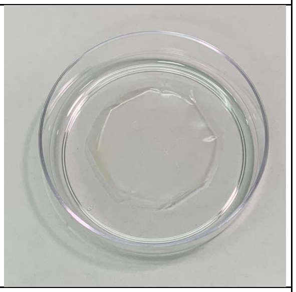
111	112	113	114	115
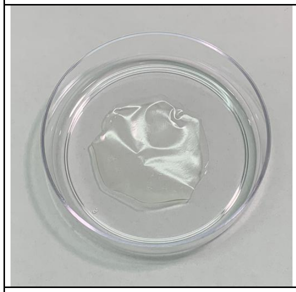	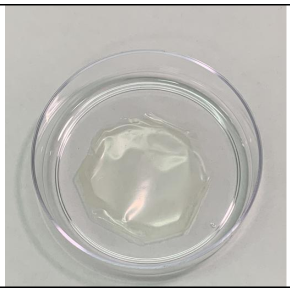	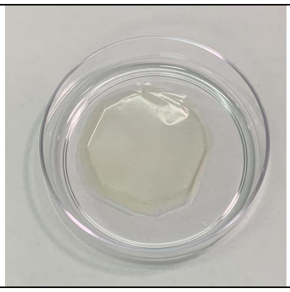	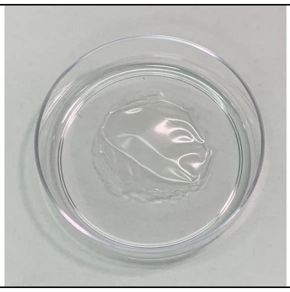	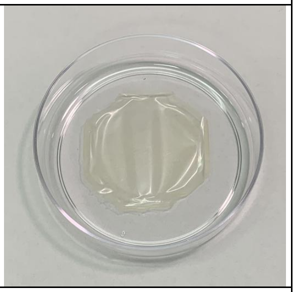
116	117	118	119	120
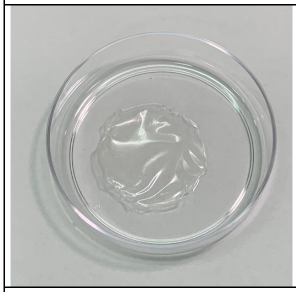	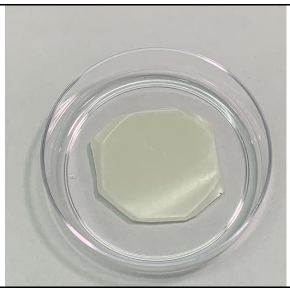	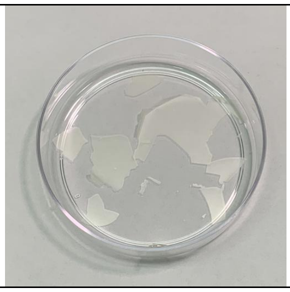	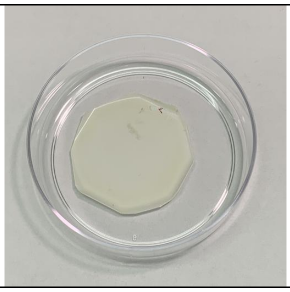	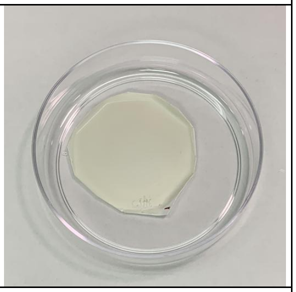
121	122	123	124	125
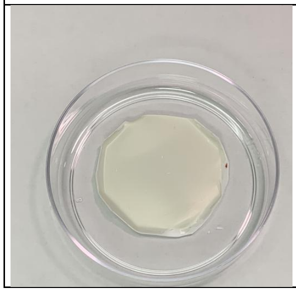	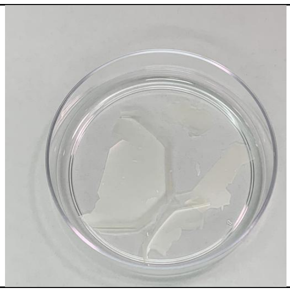	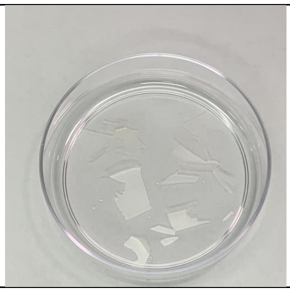	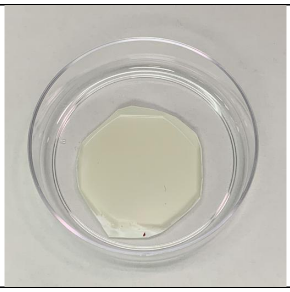	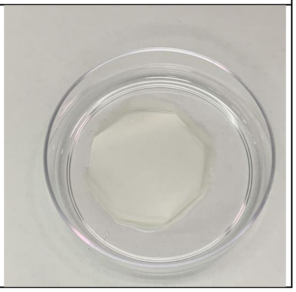

126	127	128	129	130
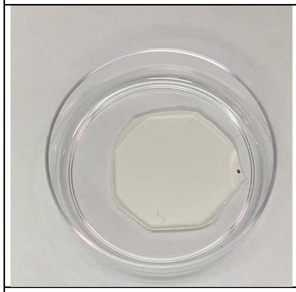	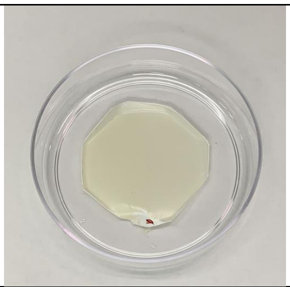	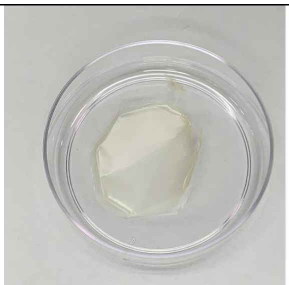	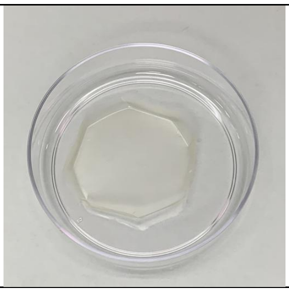	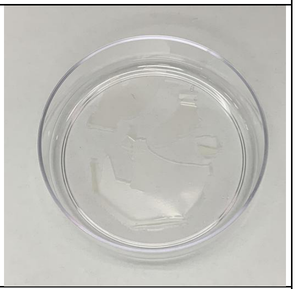
131	132	133	134	135
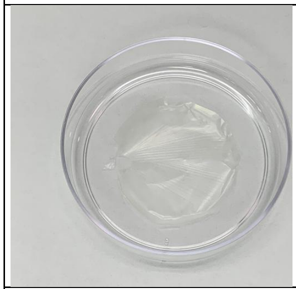	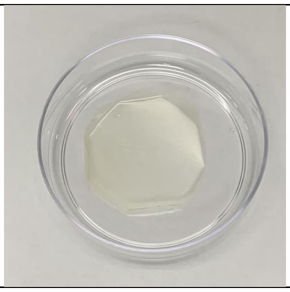	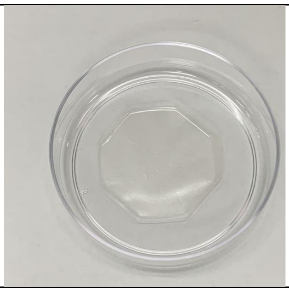	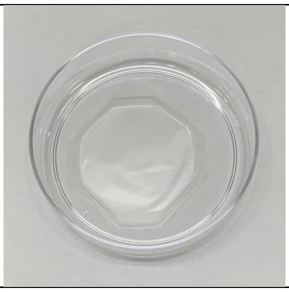	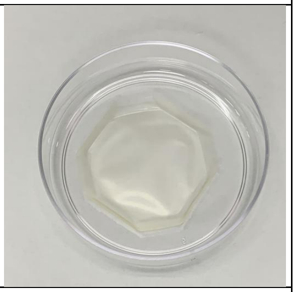
136	137	138	139	140
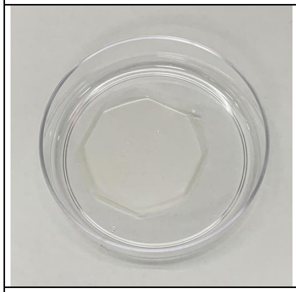	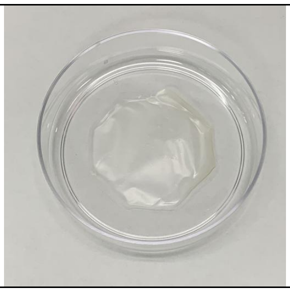	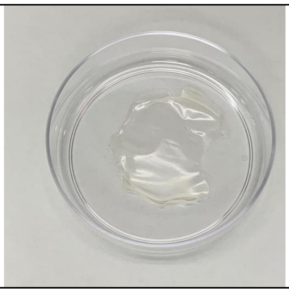	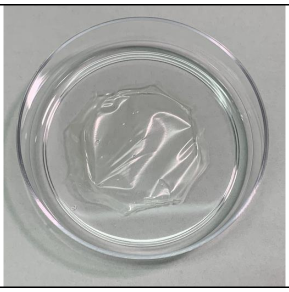	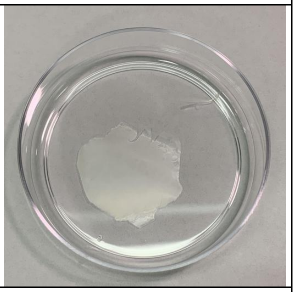
141	142	143	144	145
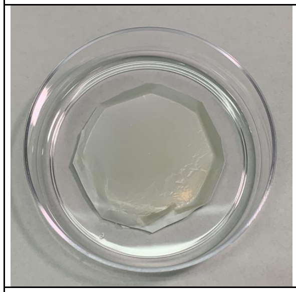	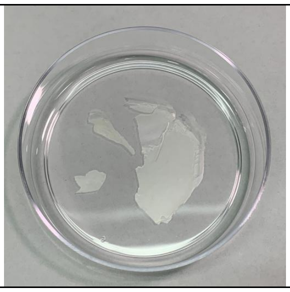	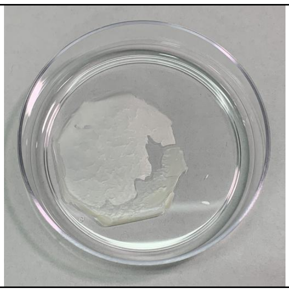	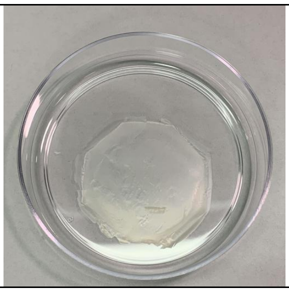	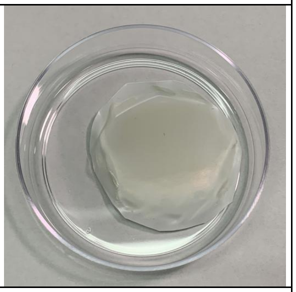
146	147	148	149	150
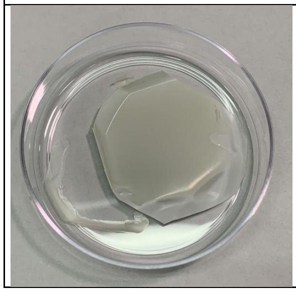	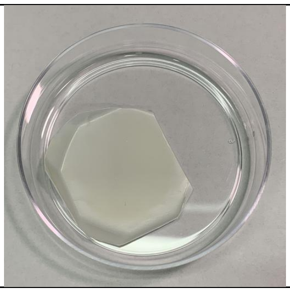	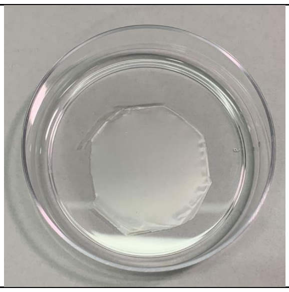	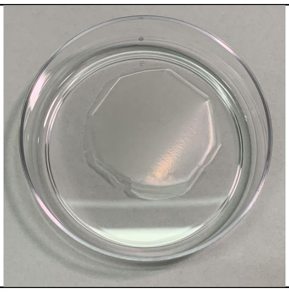	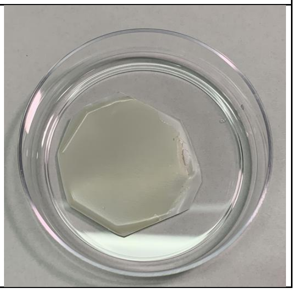

151	152	153	154	155
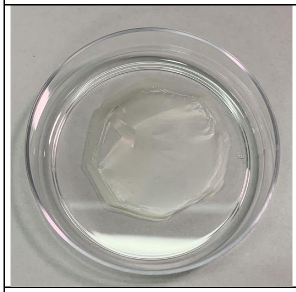	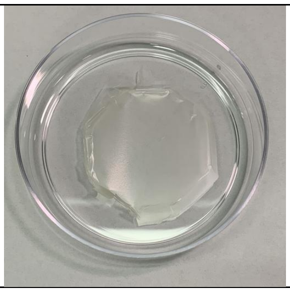	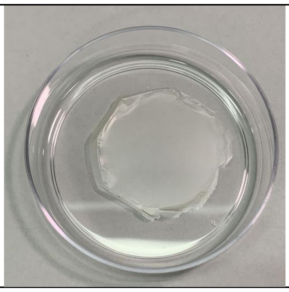	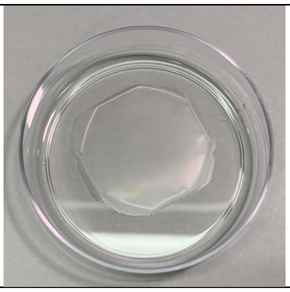	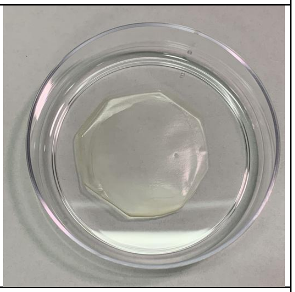
156	157	158	159	160
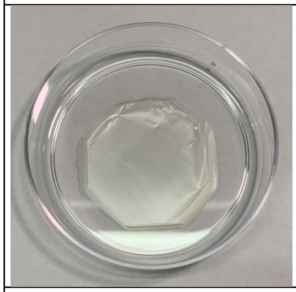	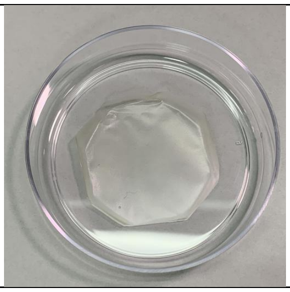	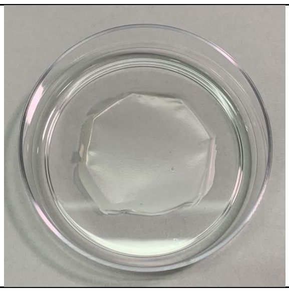	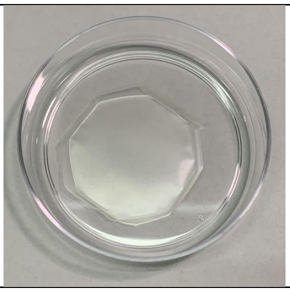	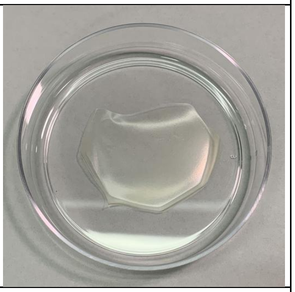
161	162	163	164	165
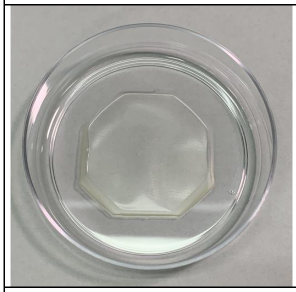	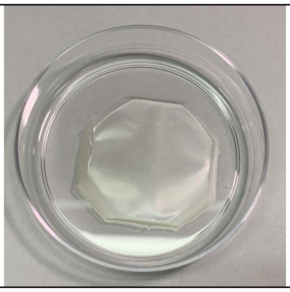	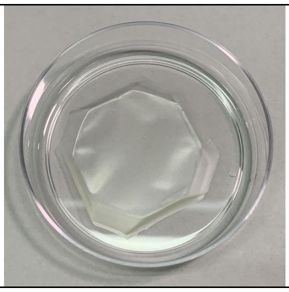	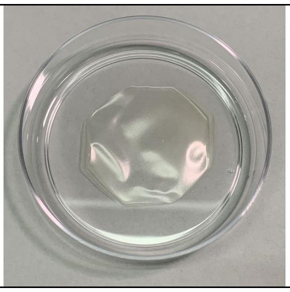	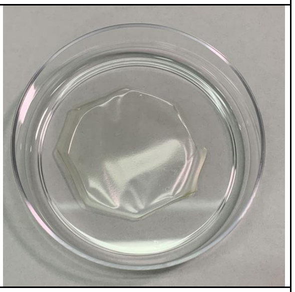
166	167	168	169	170
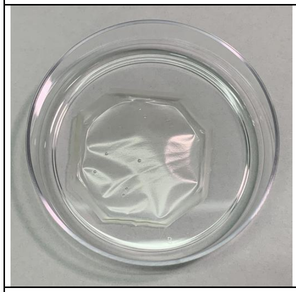	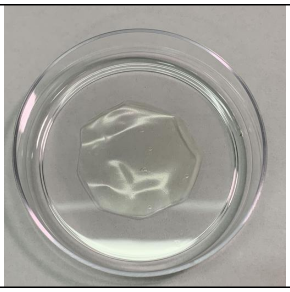	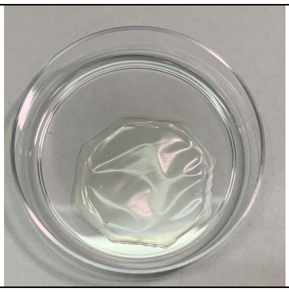	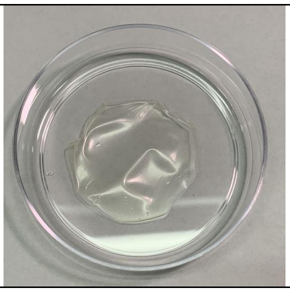	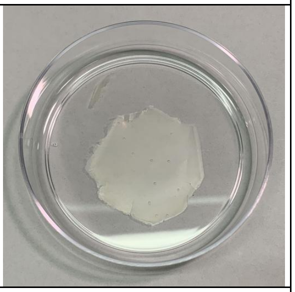
171	172	173	174	175
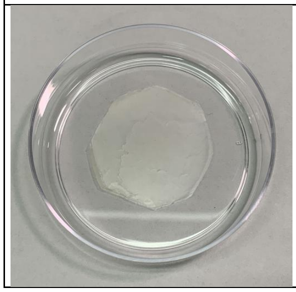	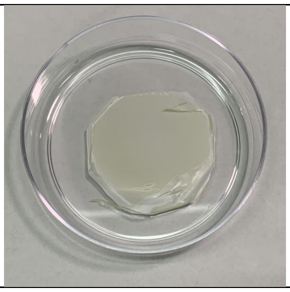	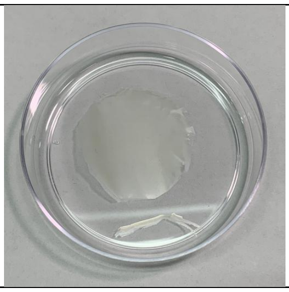	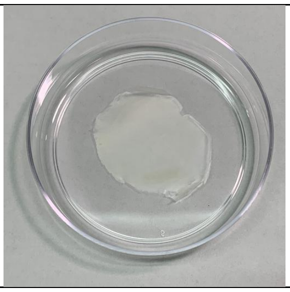	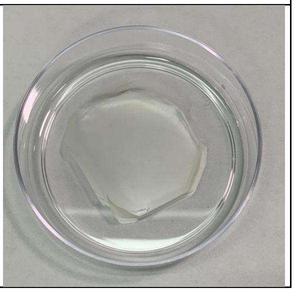

176	177	178	179	180
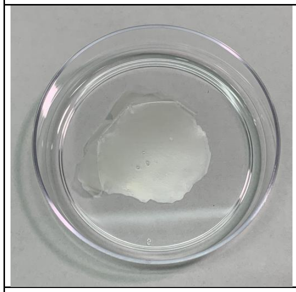	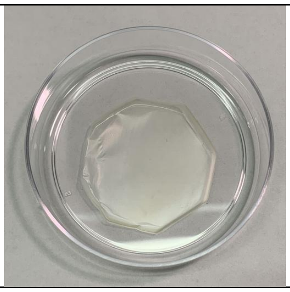	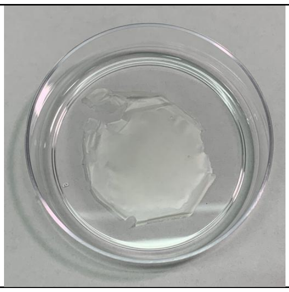	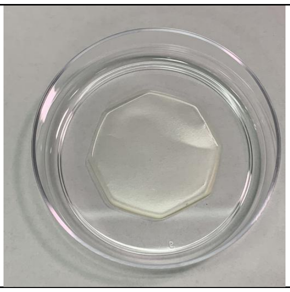	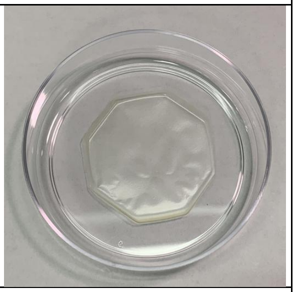
181	182	183	184	185
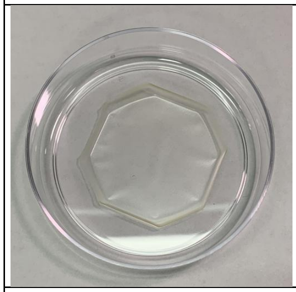	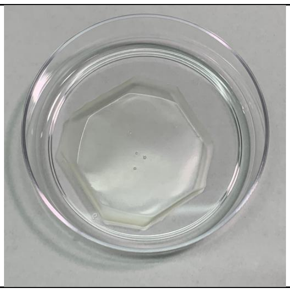	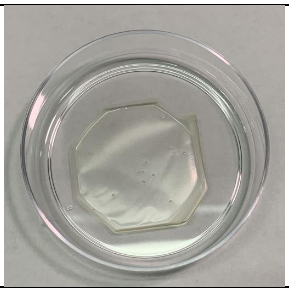	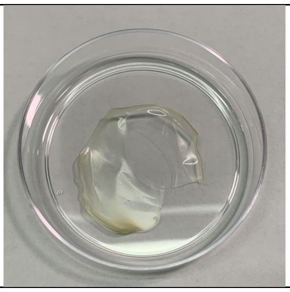	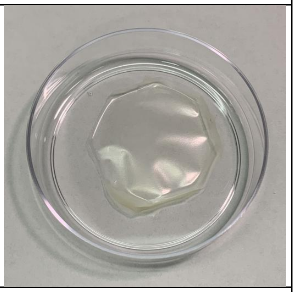
186	187	188	189	190
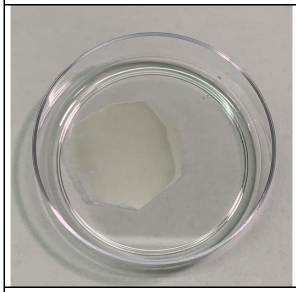	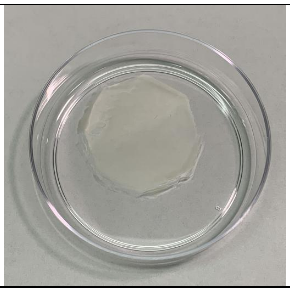	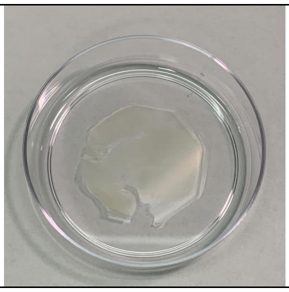	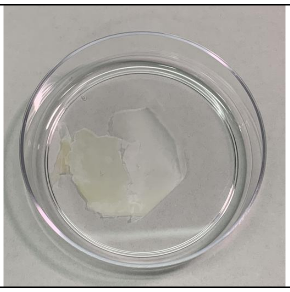	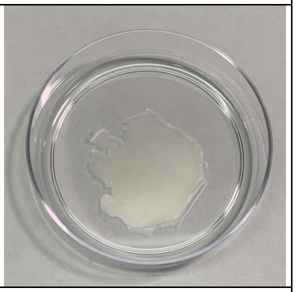
191	192	193	194	195
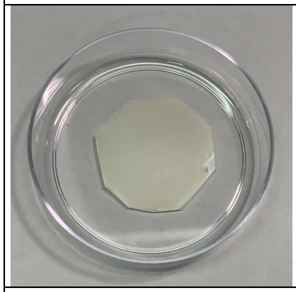	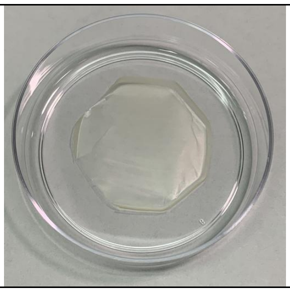	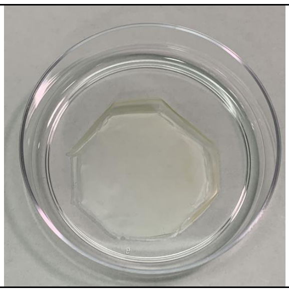	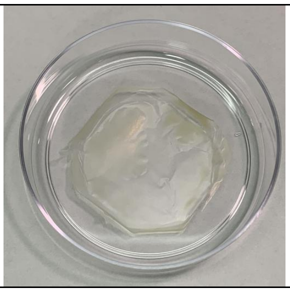	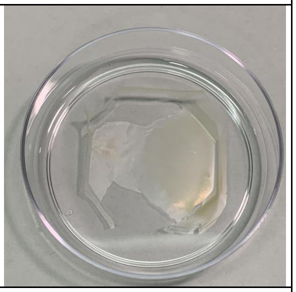
196	197	198	199	200
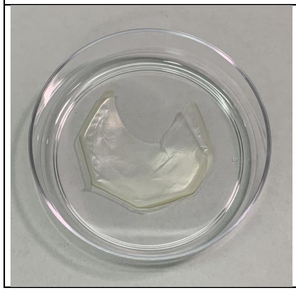	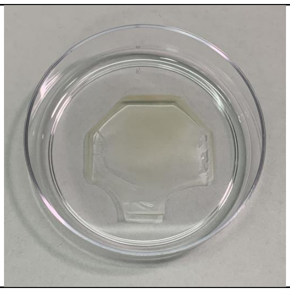	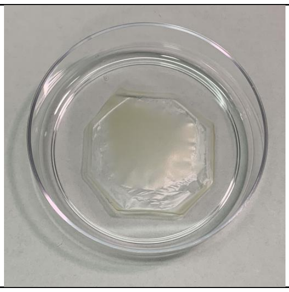	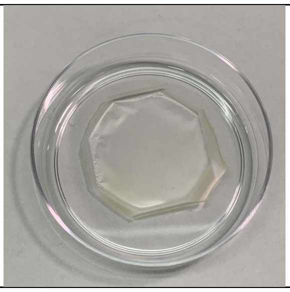	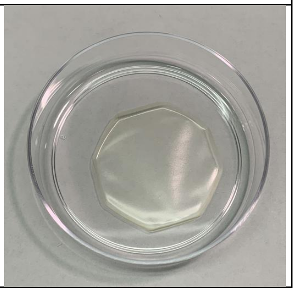

201	202	203	204	205
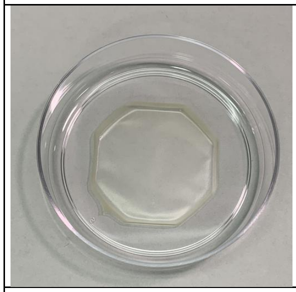	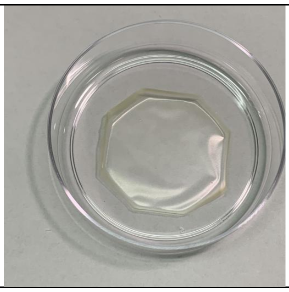	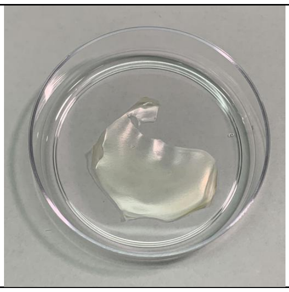	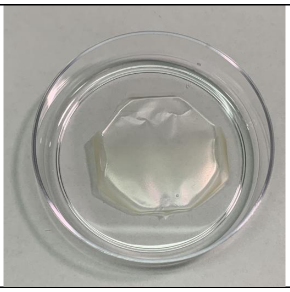	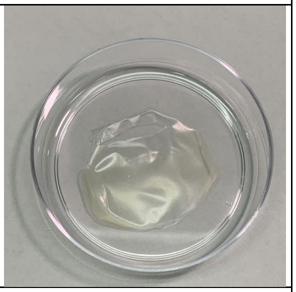
206	207	208	209	210
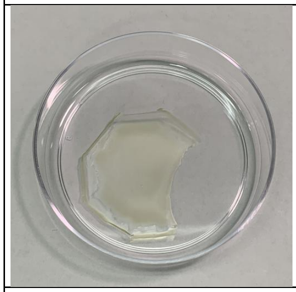	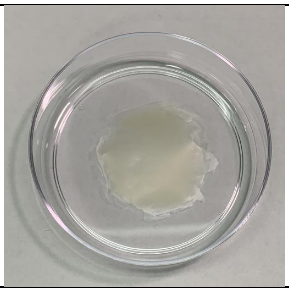	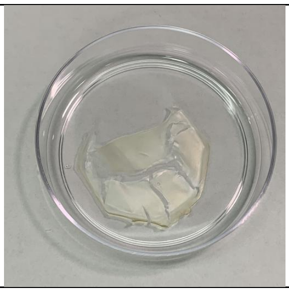	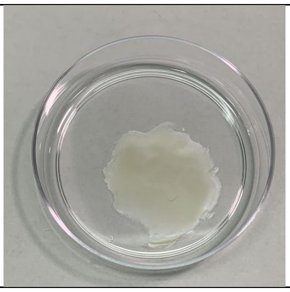	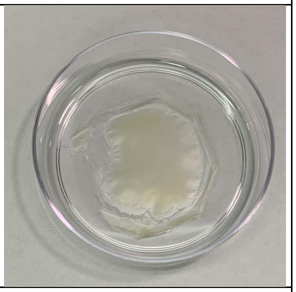
211	212	213	214	215
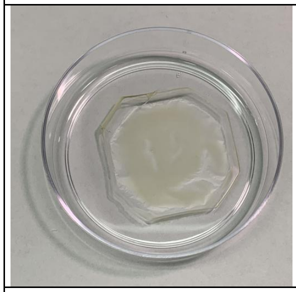	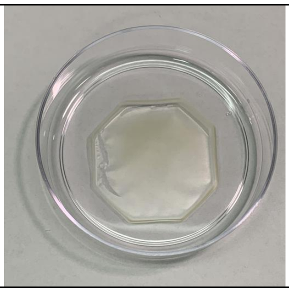	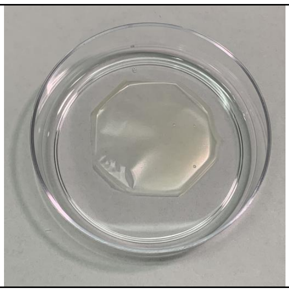	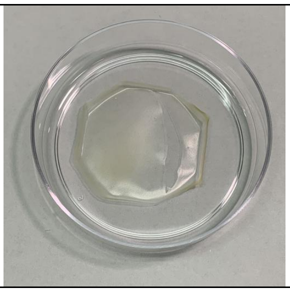	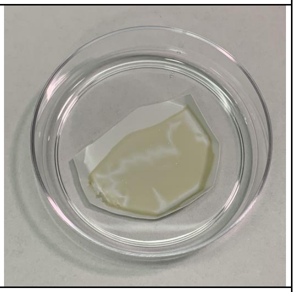
216	217	218	219	220
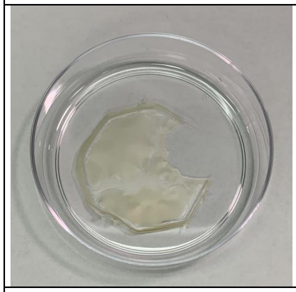	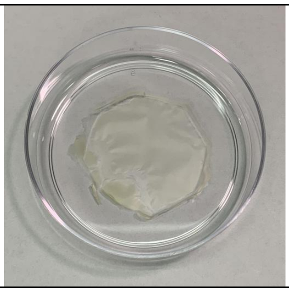	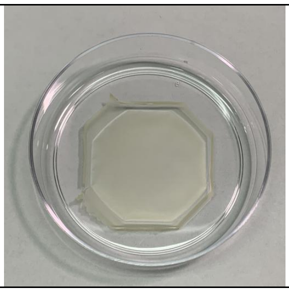	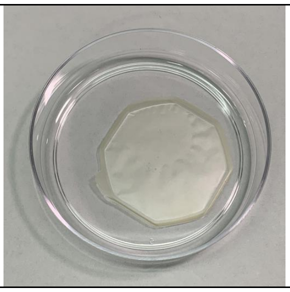	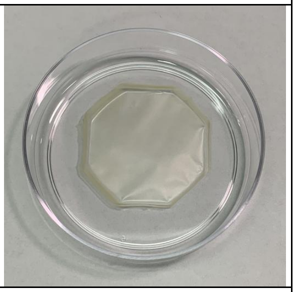
221	222	223	224	225
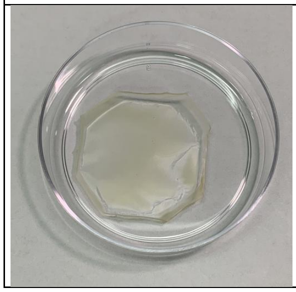	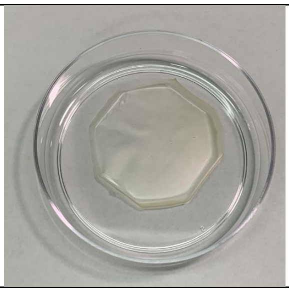	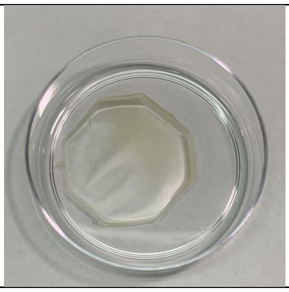	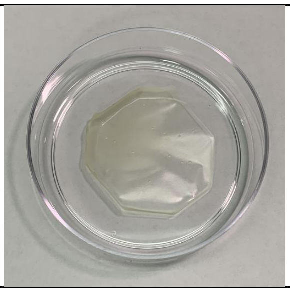	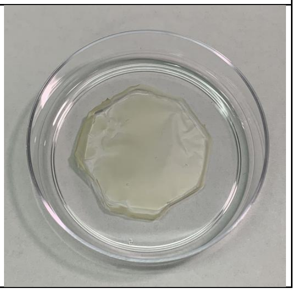

226	227	228	229	230
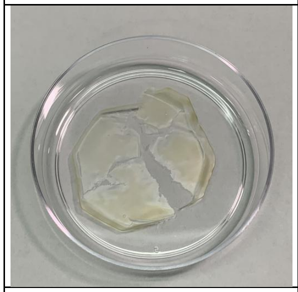	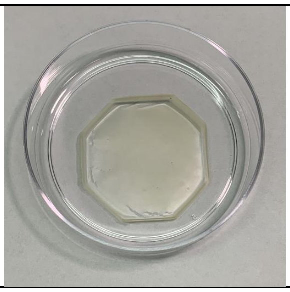	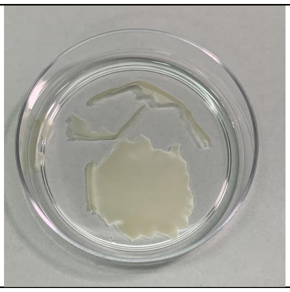	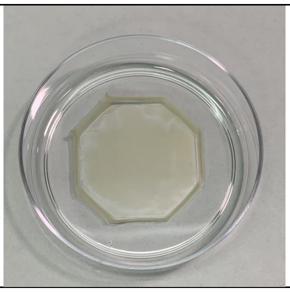	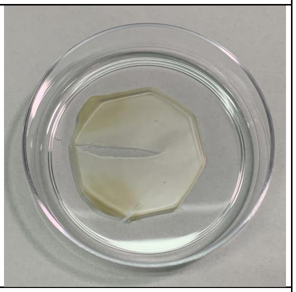
231	232	233	234	235
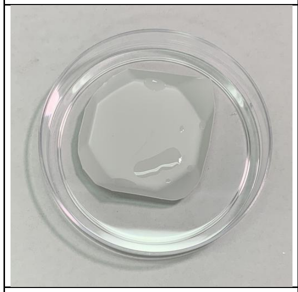	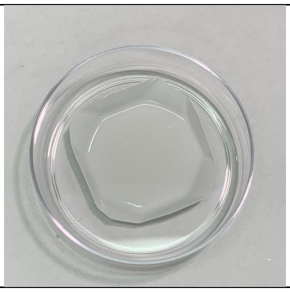	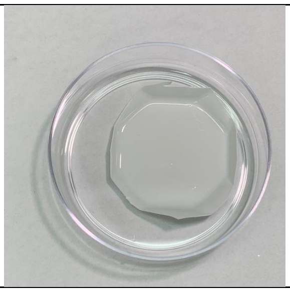	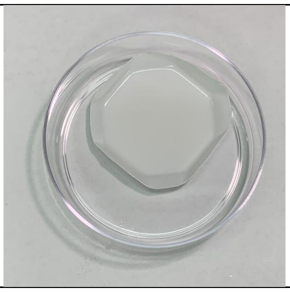	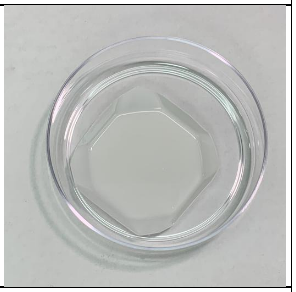
236	237	238	239	240
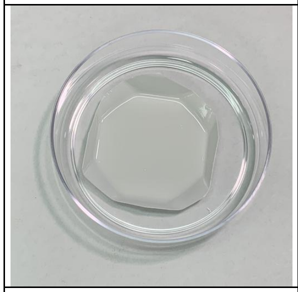	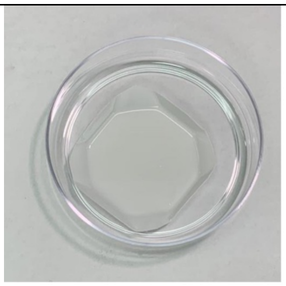	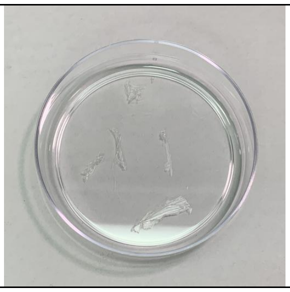	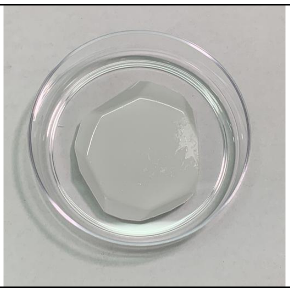	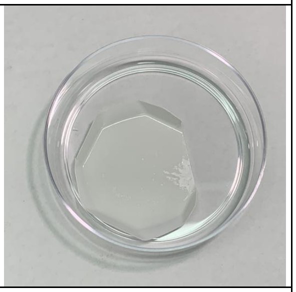
241	242	243	244	245
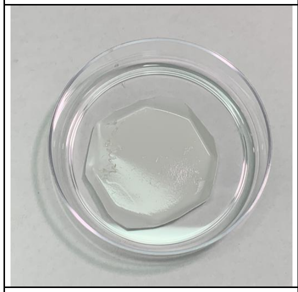	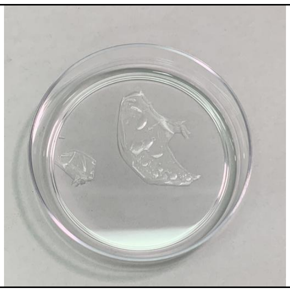	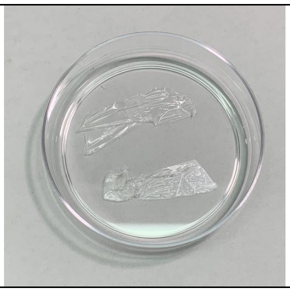	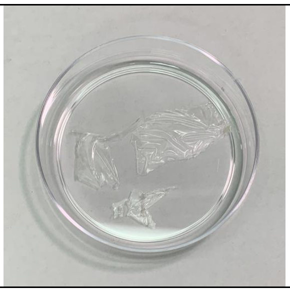	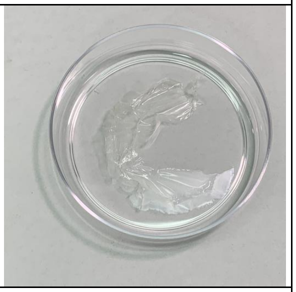
246	247	248	249	250
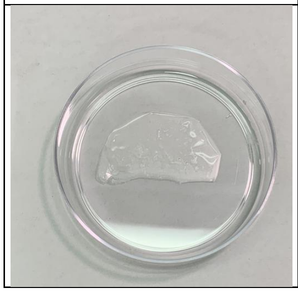	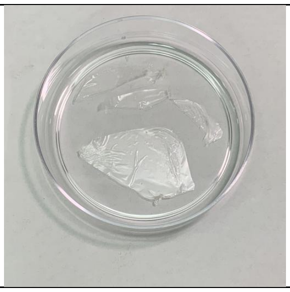	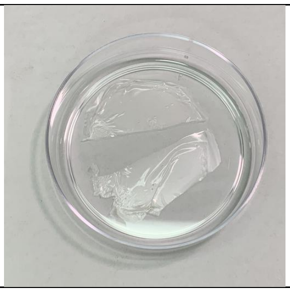	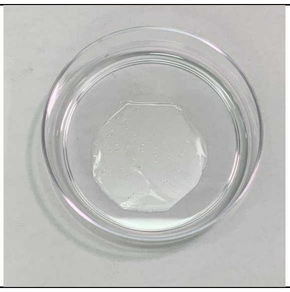	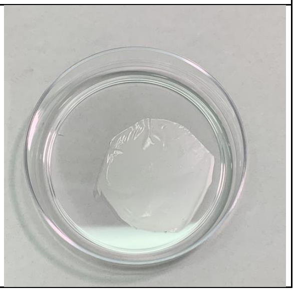

251	252	253	254	255
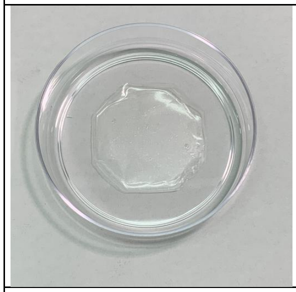	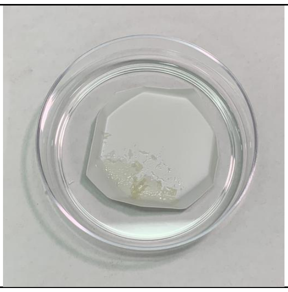	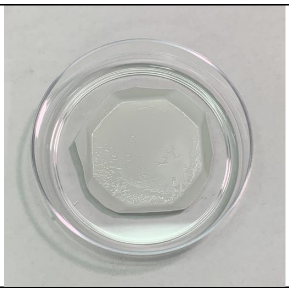	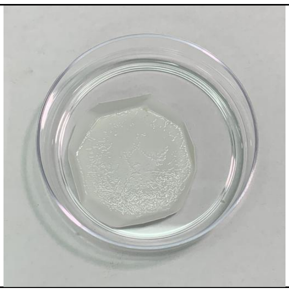	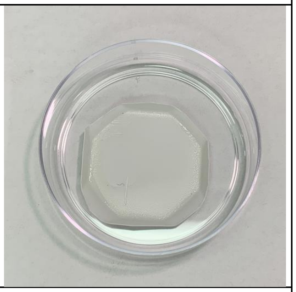
256	257	258	259	260
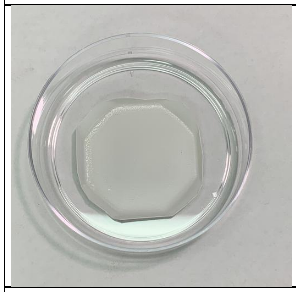	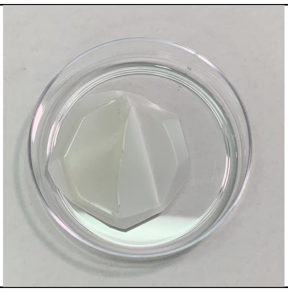	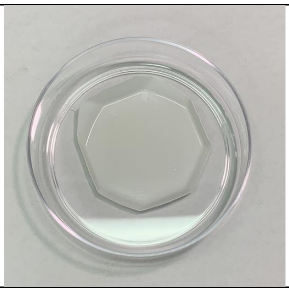	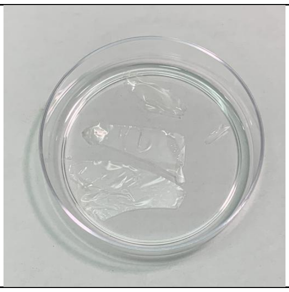	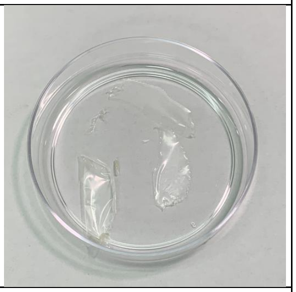
261	262	263	264	265
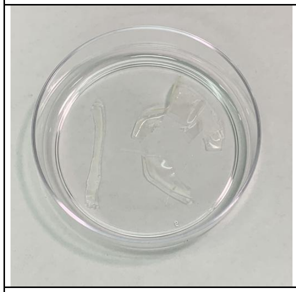	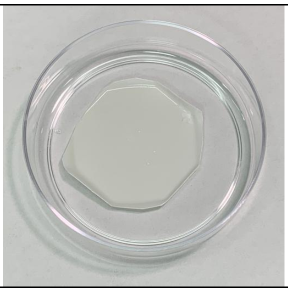	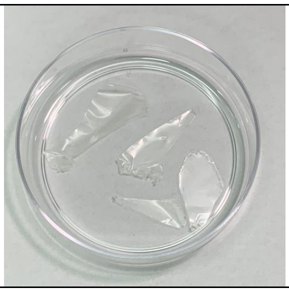	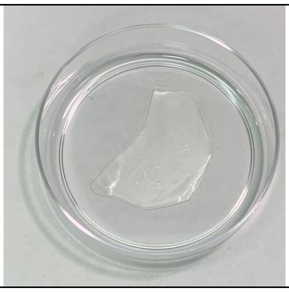	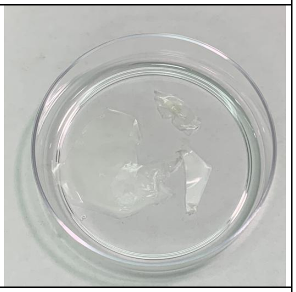
266	267	268	269	270
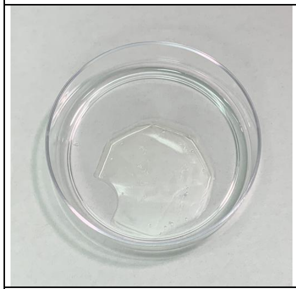	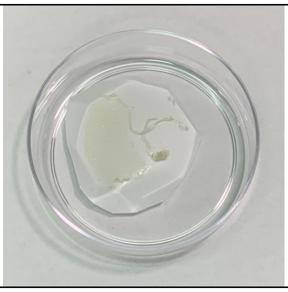	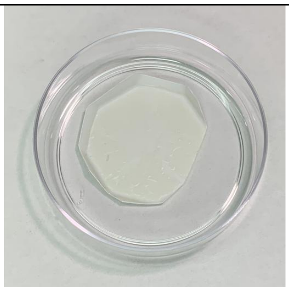	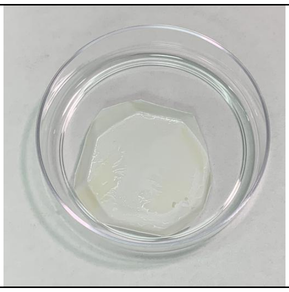	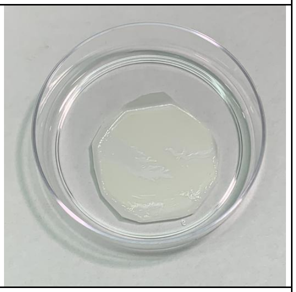
271	272	273	274	275
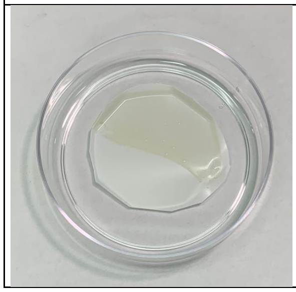	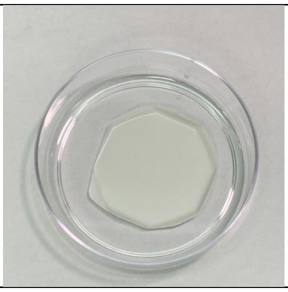	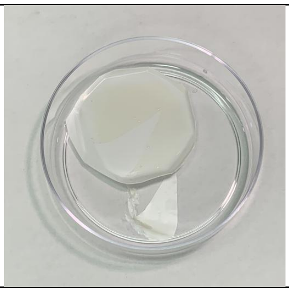	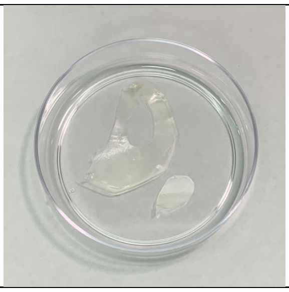	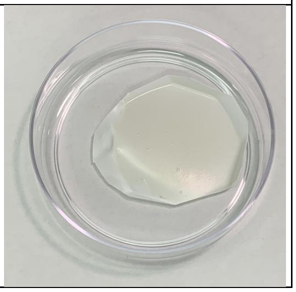

276	277	278	279	280
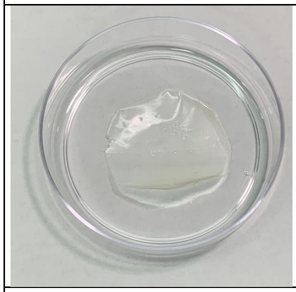	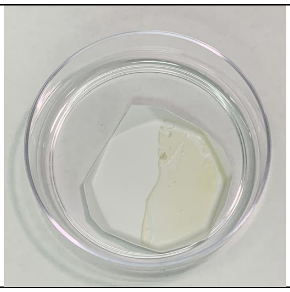	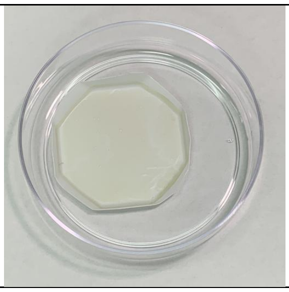	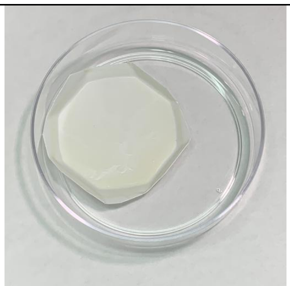	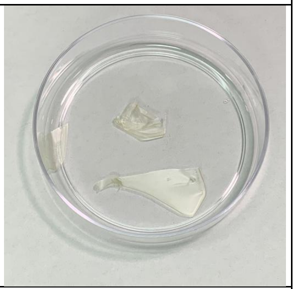
281	282	283	284	285
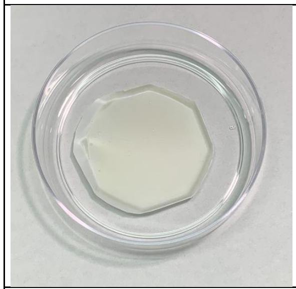	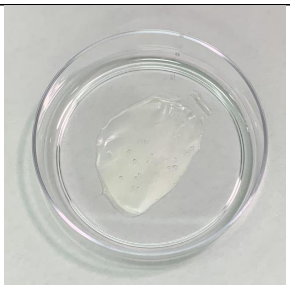	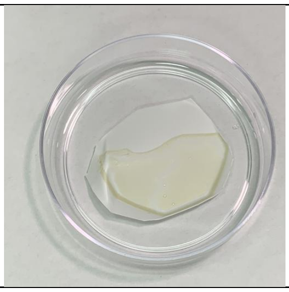	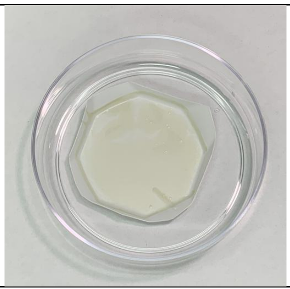	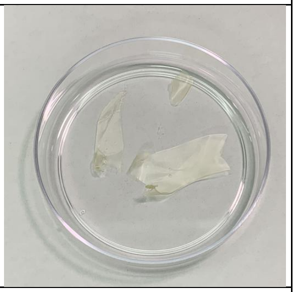
286				
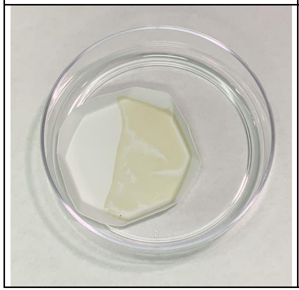				

Table S3: Testing dataset for the SVM classifier

ID	MMT/CNF/Gelatin/Glycerol Ratio				Grade
	MMT	CNF	Gelatin	Glycerol	
1	0.1364	0.0599	0.1213	0.6824	D
2	0.0739	0.1521	0.1616	0.6124	D
3	0.1108	0.1929	0.4995	0.1968	D
4	0.0554	0.1575	0.6051	0.1820	D
5	0.0262	0.1603	0.7456	0.0679	B
6	0.2578	0.5642	0.1324	0.0456	B
7	0.0324	0.0935	0.0653	0.8088	D
8	0.1660	0.6248	0.0562	0.1530	B
9	0.4638	0.1248	0.3125	0.0989	D
10	0.1630	0.1825	0.5412	0.1133	B
11	0.0805	0.2317	0.2076	0.4802	D
12	0.1007	0.0282	0.0679	0.8032	D
13	0.1321	0.2064	0.0761	0.5854	D
14	0.2013	0.1221	0.6296	0.0470	A
15	0.1699	0.1494	0.5141	0.1666	B
16	0.0135	0.3889	0.3879	0.2097	A
17	0.4540	0.0177	0.5130	0.0153	A
18	0.0137	0.3323	0.0203	0.6337	A
19	0.0181	0.4701	0.4936	0.0182	A
20	0.0136	0.4398	0.0484	0.4982	A
21	0.4275	0.0231	0.4094	0.1400	A
22	0.4940	0.1125	0.1645	0.2290	A
23	0.2053	0.3955	0.2692	0.1300	A
24	0.0128	0.6302	0.3424	0.0146	A
25	0.6424	0.0179	0.2131	0.1266	A
26	0.0589	0.3605	0.0115	0.5691	A
27	0.0169	0.3383	0.6075	0.0373	A
28	0.3559	0.2961	0.0854	0.2626	A
29	0.7163	0.0202	0.2495	0.0140	A
30	0.5200	0.2696	0.0193	0.1911	A
31	0.5123	0.2789	0.1932	0.0156	A
32	0.0156	0.5359	0.1822	0.2663	A
33	0.6531	0.1377	0.1929	0.0163	A
34	0.0181	0.6475	0.2233	0.1111	B
35	0.3280	0.2408	0.4183	0.0129	A

Table S4: Training dataset for the prediction model.

Composition Labels				Property Labels								
MMT (wt.%)	CNF (wt.%)	Gelatin (wt.%)	Glycerol (wt.%)	T_{Vis} (%)	T_{IR} (%)	T_{UV} (%)	RR (–)	σ_u (MPa)	ε_f (%)	E (MPa)	α (–)	β (–)
1.35	38.89	38.79	20.97	81.9	58.0	84.2	0.18	11.32	8.99	1037.05	0.80	0.05
45.40	1.77	51.30	1.53	61.6	22.8	74.5	0.80	107.75	4.61	3569.51	0.88	0.92
1.37	33.23	2.03	63.37	86.3	66.1	86.4	0.40	6.64	3.45	613.90	0.80	0.15
1.81	47.01	49.36	1.82	76.9	57.4	80.1	0.10	63.96	4.43	2773.06	0.56	0.05
1.36	43.98	4.84	49.82	86.7	64.2	88.4	0.80	21.68	5.82	1695.00	0.75	0.12
42.75	2.31	40.94	14.00	66.2	32.2	75.3	0.10	39.15	1.95	3627.78	0.62	0.04
49.40	11.25	16.45	22.90	33.8	2.7	71.4	0.78	31.66	1.70	3607.94	0.73	0.05
20.53	39.55	26.92	13.00	77.4	52.9	78.9	0.75	37.83	2.81	3242.28	0.81	0.68
1.28	63.02	34.24	1.46	85.5	72.5	82.8	0.10	68.26	3.65	3445.08	0.85	0.67
64.24	1.79	21.31	12.66	28.3	2.7	60.5	0.88	105.42	2.09	9754.08	0.28	0.02
43.73	21.76	33.41	1.10	66.4	25.5	69.6	0.77	21.40	0.58	4312.69	0.47	0.09
45.50	34.69	6.53	13.28	81.8	57.7	82.6	0.70	59.62	1.15	6911.20	0.80	0.34
35.57	21.75	17.46	25.22	72.9	36.8	72.4	0.72	30.39	1.15	4197.96	0.77	0.29
23.84	41.38	33.41	1.37	81.1	63.8	78.1	0.74	23.05	0.46	4154.40	0.58	0.36
1.39	53.34	30.45	14.82	83.2	63.7	79.7	0.18	67.88	6.10	4215.41	0.94	0.09
2.62	41.38	6.38	49.62	84.3	61.1	77.0	0.14	10.80	8.64	1086.98	0.80	0.14
1.91	61.77	14.22	22.10	84.7	58.3	83.7	0.11	52.71	9.83	3598.05	0.79	0.00
1.83	39.92	14.16	44.09	86.0	59.3	81.5	0.10	24.83	19.92	1942.93	0.77	0.02
4.44	41.16	2.95	51.45	85.7	55.9	85.9	0.21	12.53	4.08	1381.63	0.78	0.04
43.02	20.44	34.44	2.10	67.7	39.2	68.7	0.94	69.74	1.88	5489.13	0.65	0.35
57.66	5.12	23.96	13.26	43.1	8.0	69.8	0.90	59.78	0.97	7157.16	0.71	0.37
47.81	15.42	14.72	22.05	66.9	16.8	76.5	0.68	43.17	1.22	5266.89	0.82	0.11
1.90	42.28	12.31	43.51	81.9	48.5	78.5	0.11	11.97	29.31	637.82	0.66	0.24
5.45	66.15	9.46	18.94	85.3	59.4	89.8	0.21	52.70	6.77	3195.18	0.69	0.03

Composition Labels				Property Labels								
46.82	19.40	32.16	1.62	56.6	14.9	68.1	0.90	89.67	2.94	4418.28	0.39	0.08
19.76	32.62	29.80	17.82	78.5	45.4	75.8	0.45	49.07	3.81	4658.42	0.84	0.12
12.21	51.29	32.43	4.07	80.1	57.2	79.1	0.58	73.67	1.44	8190.37	0.51	0.05
42.86	35.30	3.32	18.52	75.1	34.9	79.5	0.69	66.25	2.41	5325.95	0.82	0.36
59.98	20.43	17.21	2.38	30.0	8.3	59.2	0.79	82.75	3.31	6110.24	0.77	0.46
23.21	37.36	34.46	4.97	79.3	53.2	76.7	0.71	64.67	0.91	5730.83	0.13	0.04
22.83	34.13	3.43	39.61	82.2	53.6	88.4	0.62	25.75	2.13	3370.62	0.82	0.06
2.71	38.31	57.07	1.91	72.7	54.4	71.3	0.30	58.37	1.48	6037.15	0.24	0.05
37.61	21.00	11.38	30.01	75.9	44.8	74.1	0.61	38.07	1.11	5748.10	0.82	0.33
60.92	14.45	11.70	12.93	30.1	7.8	68.9	0.97	46.13	1.60	4196.23	0.40	0.05
28.31	29.16	14.58	27.95	75.3	44.8	77.1	0.76	40.25	1.89	3681.73	0.89	0.30
12.33	43.54	30.66	13.47	79.6	57.4	77.3	0.70	73.79	2.86	3882.50	0.79	0.67
7.53	43.10	12.93	36.44	84.1	59.1	81.7	0.42	21.77	6.33	2337.79	0.77	0.05
4.54	48.09	14.32	33.05	84.0	59.8	82.0	0.24	33.71	13.20	4035.36	0.77	0.11
57.40	17.86	11.58	13.16	63.9	20.5	74.7	0.78	40.29	1.28	4927.50	0.57	0.14
40.67	35.64	15.11	8.58	76.7	34.0	80.5	0.69	47.73	1.01	6427.98	0.59	0.35
3.23	32.73	51.66	12.38	73.7	50.2	72.6	0.34	47.20	4.48	3366.43	0.95	0.06
3.50	37.09	52.09	7.32	76.6	55.2	70.4	0.19	63.16	2.34	3875.67	0.86	0.57
5.17	50.46	22.49	21.88	84.1	63.3	82.0	0.36	20.93	6.87	2129.23	0.72	0.08
34.56	34.25	27.73	3.46	75.2	45.7	78.0	0.68	54.67	1.01	6501.85	0.66	0.41
39.19	13.41	32.06	15.34	67.9	30.5	70.3	0.91	53.24	1.76	5107.29	0.76	0.51
30.70	42.82	15.73	10.75	75.8	46.8	83.8	0.39	28.83	0.79	4627.50	0.70	0.41
6.37	39.70	1.30	52.63	85.6	65.6	87.6	0.65	6.61	3.63	953.41	0.67	0.20
75.61	6.56	11.57	6.26	31.8	5.2	64.0	0.98	60.91	1.98	4372.35	0.31	0.03
56.80	28.42	4.91	9.87	82.6	56.9	83.6	0.79	49.89	1.25	6188.17	0.54	0.10
8.40	50.00	28.14	13.46	80.2	56.3	78.6	0.58	65.44	4.26	4125.97	0.90	0.10
9.02	52.11	14.27	24.60	81.4	49.6	84.1	0.48	42.67	7.03	3588.49	0.77	0.02
30.36	29.60	10.32	29.72	76.1	32.2	77.5	0.71	28.21	2.21	3242.01	0.82	0.09

Composition Labels				Property Labels								
2.35	57.87	3.43	36.35	84.6	56.1	84.6	0.80	19.40	7.16	1632.96	0.64	0.13
29.47	28.14	12.38	30.01	73.3	44.2	75.1	0.66	25.56	1.88	2835.14	0.81	0.22
10.13	51.97	19.87	18.03	81.0	56.1	78.8	0.44	51.65	6.42	4065.59	0.81	0.05
63.42	12.10	4.37	20.11	67.5	26.4	75.1	0.75	30.59	1.46	3428.36	0.71	0.16
32.81	27.47	20.44	19.28	75.9	41.1	76.5	0.67	41.59	1.15	4603.20	0.81	0.24
9.59	38.83	15.49	36.09	75.6	52.3	77.4	0.49	12.02	8.26	1083.36	0.74	0.08
52.05	1.85	22.83	23.27	67.2	26.8	70.3	0.89	33.85	0.96	4277.91	0.71	0.12
1.56	53.59	18.22	26.63	83.1	41.8	86.2	0.12	28.02	18.58	1929.38	0.78	0.06
5.03	34.37	1.21	59.39	80.0	52.0	75.7	0.30	5.05	5.84	493.31	0.79	0.22
6.36	35.30	45.99	12.35	71.8	47.9	67.0	0.48	77.06	1.98	4398.16	0.76	0.46
3.28	47.73	41.36	7.63	74.8	52.1	71.1	0.15	129.28	2.53	6206.98	0.77	0.75
3.22	63.53	28.54	4.71	80.3	56.9	77.0	0.16	138.93	2.16	7470.97	0.68	0.81
38.93	19.85	37.76	3.46	69.8	41.6	71.5	0.73	29.48	0.50	4049.18	0.10	0.05
5.04	42.54	44.30	8.12	73.8	50.5	71.8	0.35	102.47	1.85	6280.07	0.71	0.68
50.27	9.97	38.16	1.60	63.3	23.6	65.5	0.70	34.93	0.58	6610.64	0.48	0.03
12.32	38.68	35.67	13.33	77.3	52.9	74.4	0.75	96.67	3.15	5825.10	0.92	0.24
32.99	23.77	34.20	9.04	77.4	27.9	78.6	0.71	54.94	1.03	5844.69	0.30	0.06
52.93	15.55	28.71	2.81	43.2	14.4	62.9	0.81	56.23	1.33	5856.07	0.58	0.08
68.73	7.83	19.18	4.26	32.8	3.4	59.5	0.89	52.75	0.87	7231.47	0.36	0.00
40.29	26.61	30.42	2.68	76.5	50.6	78.3	0.72	45.61	0.51	7474.06	0.24	0.04
4.24	55.83	26.16	13.77	83.9	62.9	83.8	0.17	145.34	4.64	6236.95	0.94	0.24
50.98	23.24	3.44	22.34	70.7	38.4	79.7	0.80	63.18	1.60	5751.74	0.80	0.25
12.33	43.49	32.80	11.38	82.6	62.0	81.9	0.58	125.39	2.94	6527.86	0.88	0.34
41.81	26.94	10.82	20.43	77.3	35.1	81.5	0.65	73.75	2.15	5484.44	0.78	0.08
10.09	42.91	45.00	2.00	81.8	59.6	82.3	0.63	38.10	0.56	5323.96	0.26	0.05
7.45	42.64	25.93	23.98	83.0	58.5	82.9	0.57	40.40	9.22	3540.24	0.80	0.04
26.43	33.07	28.63	11.87	81.1	50.0	83.3	0.67	86.59	1.33	7164.76	0.75	0.42
40.73	23.12	25.72	10.43	76.4	44.8	80.1	0.86	16.29	0.53	2624.69	0.21	0.02

Composition Labels				Property Labels								
6.41	32.17	59.50	1.92	82.5	67.3	81.8	0.61	10.26	0.41	2887.97	0.42	0.08
1.52	55.78	14.23	28.47	84.8	59.4	84.3	0.28	41.11	16.76	2927.89	0.79	0.00
41.72	4.78	35.21	18.29	68.0	31.1	80.7	0.59	81.61	1.70	5727.15	0.76	0.43
58.98	5.80	33.21	2.01	51.3	18.5	69.7	0.85	107.78	1.36	8803.53	0.58	0.83
7.76	59.04	27.17	6.03	84.7	70.0	84.3	0.29	140.38	2.69	6955.95	0.71	0.84
47.80	3.93	40.41	7.86	65.2	26.4	72.7	0.69	20.23	0.87	2651.15	0.69	0.20
49.32	8.29	23.37	19.02	66.9	8.6	75.6	1.00	83.97	1.03	9290.25	0.84	0.56
14.99	32.87	32.53	19.61	82.3	62.4	80.4	0.56	87.01	3.25	6000.61	0.90	0.17
51.83	20.43	18.55	9.19	41.5	8.2	77.8	0.69	34.95	0.76	6569.81	0.71	0.57
9.01	45.48	36.49	9.02	83.0	57.8	82.6	0.60	125.93	2.12	6773.17	0.58	0.49
5.52	46.37	12.15	35.96	85.7	64.7	86.2	0.20	34.41	4.99	3160.97	0.86	0.05
46.69	10.93	26.32	16.06	56.5	19.5	72.9	0.92	57.99	1.08	6197.45	0.64	0.18
47.79	32.06	4.03	16.12	81.0	59.8	81.8	0.69	54.38	0.77	5976.27	0.18	0.06
52.85	21.47	16.27	9.41	53.6	10.3	73.5	0.95	78.39	1.47	6648.60	0.75	0.42
36.13	34.53	11.86	17.48	79.0	48.9	83.7	0.80	103.44	2.00	6638.22	0.75	0.39
40.85	23.33	8.61	27.21	80.7	47.5	82.9	0.65	73.99	1.77	5992.80	0.86	0.16
7.80	46.76	31.65	13.79	82.6	62.8	82.7	0.51	112.01	3.34	6371.68	0.95	0.26
54.14	10.37	34.06	1.43	48.3	11.4	68.9	0.90	65.47	1.36	5879.95	0.69	0.81
52.75	6.58	34.83	5.84	42.7	12.8	63.8	0.83	17.29	0.35	4439.10	0.28	0.50
47.46	18.73	15.10	18.71	66.8	32.6	77.0	0.76	67.77	1.42	6682.79	0.81	0.43
1.12	70.11	19.23	9.54	86.1	73.5	85.6	0.6	76.20	2.07	5716.16	0.40	0.03
38.61	3.94	51.12	6.33	67.7	34.1	69.5	0.77	81.59	3.28	5659.54	0.20	0.47
17.69	29.48	49.22	3.61	80.7	50.2	82.3	0.66	21.92	0.58	5060.67	0.87	0.32
15.33	38.88	2.37	43.42	85.8	58.7	86.5	0.60	20.93	3.36	2773.67	0.87	0.06
43.50	37.64	6.03	12.83	71.4	36.8	83.8	0.76	107.96	2.58	4518.38	0.59	0.06
66.60	13.12	13.96	6.32	37.2	1.8	65.0	0.92	132.84	3.27	7759.00	0.22	0.47
1.78	38.91	29.37	29.94	84.9	70.1	83.8	0.12	40.98	10.60	3033.53	0.84	0.04
65.07	2.91	17.57	14.45	21.4	3.7	56.7	0.92	95.71	2.29	6218.87	0.44	0.45

Composition Labels				Property Labels								
35.43	37.82	6.65	20.10	83.1	55.8	84.3	0.78	85.51	1.86	7873.81	0.67	0.53
64.86	15.53	18.14	1.47	33.4	4.1	60.4	0.82	136.93	3.74	7062.51	0.64	0.48
4.48	58.86	35.37	1.29	84.7	66.8	84.0	0.13	81.71	2.28	3988.01	0.54	0.04
44.13	11.63	33.75	10.49	52.3	12.9	72.7	0.76	37.31	1.47	4034.04	0.38	0.44
5.16	48.53	28.67	17.64	83.0	64.8	83.0	0.43	96.39	2.92	4484.02	0.60	0.43
2.51	49.21	15.12	33.16	86.4	65.5	85.3	0.18	45.24	8.45	3206.02	0.81	0.04
24.11	33.58	20.03	22.28	74.6	38.8	79.8	0.62	90.32	4.00	4023.98	0.64	0.96
1.94	43.29	22.42	32.35	85.0	64.7	85.0	0.90	37.54	10.57	2674.43	0.81	0.05
5.95	38.72	31.85	23.48	83.7	66.5	82.2	0.45	58.58	6.19	3072.59	0.93	0.13
18.88	38.48	6.12	36.52	83.4	57.0	89.7	0.68	32.40	1.79	2788.83	0.86	0.18
11.10	56.73	26.45	5.72	84.4	70.2	84.9	0.57	33.32	0.58	5645.58	0.55	0.06
62.86	22.57	10.99	3.58	35.4	2.2	70.9	0.71	113.55	4.71	6263.91	0.51	0.37
34.49	14.72	45.94	4.85	67.1	28.0	70.8	0.82	38.49	1.57	4278.90	0.63	0.45
33.73	12.80	52.05	1.42	69.4	29.8	70.9	0.77	14.33	0.44	4122.89	0.88	0.08
8.27	34.24	3.34	54.15	78.4	55.7	77.4	0.60	11.79	3.15	1262.44	0.81	0.06
37.60	5.55	46.80	10.05	65.3	30.8	65.5	0.90	14.38	0.37	4094.47	0.67	0.11
2.20	51.10	37.61	9.09	78.7	57.1	74.6	0.27	43.10	0.83	4761.24	0.49	0.08
2.53	47.38	26.05	24.04	80.5	55.1	78.3	0.34	45.37	4.78	3069.20	0.89	0.16
11.19	35.38	34.02	19.41	76.9	53.9	72.7	0.65	67.54	2.30	4363.22	0.73	0.15
17.89	37.48	21.77	22.86	80.9	45.5	79.3	0.65	67.54	1.82	5694.22	0.86	0.18
8.37	35.98	47.04	8.61	71.5	51.2	69.2	0.63	54.97	1.45	6499.51	0.34	0.05
15.61	43.42	36.15	4.82	78.2	58.9	76.8	0.71	40.28	0.83	4239.97	0.56	0.10
47.93	7.15	32.29	12.63	56.8	14.3	66.2	0.91	46.99	1.36	4982.53	0.34	0.04
24.90	38.29	13.83	22.98	78.2	42.3	80.7	0.72	53.98	2.05	4304.47	0.59	0.36
55.01	15.04	11.38	18.57	45.0	17.0	64.5	0.91	56.53	1.79	5529.79	0.56	0.46
37.26	19.29	22.25	21.20	70.2	40.5	69.1	0.84	67.92	2.25	2912.49	0.86	0.03
38.45	31.02	9.25	21.28	78.3	28.0	81.9	0.78	51.22	1.22	6139.92	0.77	0.03
5.89	36.05	1.15	56.91	84.6	57.7	86.1	0.26	7.87	3.43	600.32	0.75	0.02

Composition Labels				Property Labels								
1.69	33.83	60.75	3.73	80.7	59.7	80.6	0.14	30.24	1.03	3147.06	0.30	0.05
35.59	29.61	8.54	26.26	54.5	20.6	80.4	0.87	32.44	2.23	3452.66	0.60	0.03
71.63	2.02	24.95	1.40	21.3	2.6	51.2	1.00	92.05	3.52	5048.83	0.62	0.60
52.00	26.96	1.93	19.11	68.0	32.6	82.3	0.83	34.83	1.78	3996.62	0.64	0.01
51.23	27.89	19.32	1.56	70.1	20.2	84.7	0.84	41.21	1.33	5468.02	0.51	0.03
13.86	36.97	30.59	18.58	81.1	58.4	80.4	0.67	43.04	2.66	3960.74	0.68	0.46
58.42	3.19	24.13	14.26	34.4	5.3	68.5	0.93	67.61	1.90	6707.23	0.61	0.47
39.61	33.99	17.36	9.04	70.7	28.9	85.3	0.75	65.83	2.70	5187.90	0.19	0.03
27.24	35.43	11.46	25.87	75.6	56.1	82.6	0.85	37.89	2.20	3994.75	0.57	0.02
9.00	50.85	11.61	28.54	86.0	65.4	86.2	0.32	39.65	6.43	3224.73	0.81	0.03
40.12	29.70	10.03	20.15	76.3	48.8	84.1	0.81	42.11	1.83	4286.95	0.45	0.01
33.02	34.95	4.58	27.45	81.0	51.5	85.7	0.70	30.39	1.44	3125.08	0.89	0.05
9.18	51.76	35.89	3.17	84.6	65.3	83.8	0.49	54.61	1.68	5164.65	0.32	0.01
1.32	48.81	24.86	25.01	84.3	61.7	83.6	0.17	28.78	15.03	2124.60	0.84	0.03
66.12	15.41	17.02	1.45	70.3	36.1	77.2	0.88	65.48	1.40	6424.88	0.24	0.13
71.63	2.02	24.95	1.40	37.1	8.3	59.9	0.93	49.86	0.92	6119.97	0.41	0.09
1.56	53.59	18.22	26.63	82.0	52.4	78.5	0.12	14.60	10.17	875.05	0.72	0.37
51.23	27.89	19.32	1.56	69.3	29.7	77.3	0.75	43.50	1.13	5702.96	0.47	0.07
66.12	15.41	17.02	1.45	40.4	10.7	63.9	0.38	51.26	1.46	4695.55	0.83	0.27
1.81	64.75	22.33	11.11	79.1	62.7	77.4	0.21	22.82	7.98	1830.99	0.67	0.02
1.28	63.02	34.24	1.46	83.6	65.4	79.7	0.28	40.33	4.14	3380.67	0.87	0.15
64.24	1.79	21.31	12.66	48.2	16.2	66.0	0.92	54.64	0.95	6961.76	0.63	0.07
35.59	29.61	8.54	26.26	77.8	45.3	77.7	0.61	12.39	0.79	2173.43	0.83	0.13
1.32	48.81	24.86	25.01	82.3	50.6	78.6	0.11	7.94	11.22	513.15	0.74	0.34
65.31	13.77	19.29	1.63	22.2	6.5	62.8	0.97	62.63	1.82	6684.05	0.18	0.02
1.81	64.75	22.33	11.11	86.7	63.9	86.4	0.90	43.00	6.26	3472.56	0.80	0.16
32.x80	24.08	41.83	1.29	70.8	46.2	75.1	0.81	60.50	2.46	3956.64	0.41	0.46

Table S5. Testing dataset for the prediction model.

Composition Labels				Property Labels								
MMT (wt.%)	CNF (wt.%)	Gelatin (wt.%)	Glycerol (wt.%)	T_{Vis} (%)	T_{IR} (%)	T_{UV} (%)	RR (–)	σ_u (MPa)	ε_f (%)	E (MPa)	α (–)	β (–)
5.89	36.05	1.15	56.91	84.6	86.1	57.7	0.26	7.87	0.03	600.32	0.75	0.02
1.69	33.83	60.75	3.73	80.7	80.6	59.7	0.14	30.24	0.01	3147.06	0.30	0.05
35.59	29.61	8.54	26.26	54.5	80.4	20.6	0.87	32.44	0.02	3452.66	0.60	0.03
13.86	36.97	30.59	18.58	81.1	58.4	80.4	0.67	43.04	0.03	3960.74	0.68	0.46
52.00	26.96	1.93	19.11	68.0	82.3	32.6	0.83	34.83	0.02	3996.62	0.64	0.01
51.23	27.89	19.32	1.56	70.1	84.7	20.2	0.84	41.21	0.01	5468.02	0.51	0.03
32.80	24.08	41.83	1.29	70.8	75.1	46.2	0.81	60.50	0.02	3956.64	0.41	0.46
58.42	3.19	24.13	14.26	34.4	68.5	5.3	0.93	67.61	0.02	6707.23	0.61	0.47
27.24	35.43	11.46	25.87	75.6	82.6	56.1	0.85	37.89	0.02	3994.75	0.57	0.02
9.00	50.85	11.61	28.54	86.0	86.2	65.4	0.32	39.65	0.06	3224.73	0.81	0.03
40.12	29.70	10.03	20.15	76.3	84.1	48.8	0.81	42.11	0.02	4286.95	0.45	0.01
33.02	34.95	4.58	27.45	81.0	85.7	51.5	0.70	30.39	0.01	3125.08	0.89	0.05
9.18	51.76	35.89	3.17	84.6	83.8	65.3	0.49	54.61	0.02	5164.65	0.32	0.01
71.63	2.02	24.95	1.40	37.1	59.9	8.3	0.93	49.86	0.01	6119.97	0.41	0.09
64.24	1.79	21.31	12.66	48.2	66.0	16.2	0.92	54.64	0.01	6961.76	0.63	0.07

Table S6. Training dataset for various prediction models based on different sampling methods.

"*rand*" refers to random sampling. "*dist*" refers to distance sampling. "*latin*" refers to Latin hypercube sampling. "*human*" refers to human intelligence sampling.

Sampling Method	Composition Labels				Film Grade	Property Labels								
	MMT (wt.%)	CNF (wt.%)	Gelatin (wt.%)	Glycerol (wt.%)		T_{Vis} (%)	T_{IR} (%)	T_{UV} (%)	RR (–)	σ_u (MPa)	ε_f (%)	E (MPa)	α (–)	β (–)
rand	62.91	27.75	5.84	3.51	C	–	–	–	–	–	–	–	–	–
rand	26.52	29.69	19.94	23.85	A	73.8	76.7	41.8	0.66	45.97	1.31	3340.27	0.65	0.48
rand	23.43	39.71	34.75	2.11	A	78.5	77.6	54.4	0.73	25.88	0.43	6644.40	0.58	0.18
rand	46.77	2.86	39.95	10.42	C	–	–	–	–	–	–	–	–	–
rand	40.02	25.44	14.49	20.05	A	75.5	78.7	39.4	0.66	52.09	0.91	4888.50	0.79	0.04
rand	3.11	9.31	44.55	43.03	C	–	–	–	–	–	–	–	–	–
rand	20.78	13.21	33.27	32.74	A	74.9	71.1	39.4	0.72	23.98	1.50	1666.08	0.85	0.01
rand	28.30	26.89	28.41	16.40	A	77.1	76.0	42.3	0.62	48.33	1.19	3929.93	0.77	0.21
rand	8.76	41.84	30.78	18.63	A	78.6	79.4	56.6	0.65	44.66	3.20	2706.70	0.80	0.33
rand	18.41	33.11	34.73	13.75	A	76.4	76.2	49.0	0.53	35.93	2.05	3835.40	0.86	0.06
rand	31.04	17.94	32.24	18.78	B	–	–	–	–	–	–	–	–	–
rand	18.60	41.15	11.17	29.08	A	78.9	81.4	51.2	0.69	24.22	3.14	2263.32	0.75	0.09
rand	5.14	42.14	39.89	12.82	A	76.6	73.7	53.9	0.34	65.87	4.25	3696.46	0.74	0.34
rand	59.71	5.23	23.72	11.34	A	53.4	72.4	19.0	0.89	48.94	0.75	7307.55	0.71	0.19
rand	29.23	50.73	15.57	4.47	A	76.9	83.4	52.5	0.47	46.13	0.86	5831.14	0.66	0.21
rand	31.35	16.14	8.18	44.33	C	–	–	–	–	–	–	–	–	–
rand	14.54	31.57	10.01	43.88	C	–	–	–	–	–	–	–	–	–
rand	18.28	5.96	30.81	44.96	C	–	–	–	–	–	–	–	–	–
rand	19.18	40.60	21.66	18.55	A	76.9	77.7	48.7	0.67	40.55	1.97	3678.74	0.86	0.09
rand	20.62	32.70	31.21	15.46	A	76.9	76.1	46.6	0.47	36.44	1.63	3837.27	0.84	0.06

Sampling Method	Composition Labels				Film Grade	Property Labels								
	MMT (wt.%)	CNF (wt.%)	Gelatin (wt.%)	Glycerol (wt.%)		T_{Vis} (%)	T_{IR} (%)	T_{UV} (%)	RR (–)	σ_u (MPa)	ε_f (%)	E (MPa)	α (–)	β (–)
rand	29.35	19.53	20.73	30.40	A	73.3	74.8	41.4	0.74	23.50	1.32	2975.42	0.79	0.15
rand	15.13	26.27	25.46	33.15	A	78.5	77.5	49.2	0.73	22.28	3.12	2310.42	0.85	0.09
rand	4.79	26.57	33.60	35.04	C	–	–	–	–	–	–	–	–	–
rand	2.63	30.06	34.06	33.24	A	81.0	80.9	61.8	0.12	23.73	9.43	1492.19	0.85	0.02
rand	5.80	34.07	31.22	28.91	A	80.1	80.7	60.9	0.24	33.67	7.63	1817.23	0.84	0.02
rand	19.86	10.03	32.64	37.47	C	–	–	–	–	–	–	–	–	–
rand	12.66	24.74	20.49	42.11	A	74.2	77.1	47.8	0.58	15.30	4.12	1845.85	0.78	0.04
rand	4.05	50.29	42.43	3.23	A	77.6	77.7	57.4	0.17	55.05	0.87	4789.36	0.66	0.37
rand	34.70	3.76	36.52	25.01	D	–	–	–	–	–	–	–	–	–
rand	47.85	4.34	43.43	4.38	B	–	–	–	–	–	–	–	–	–
rand	15.29	18.90	41.38	24.43	A	76.7	67.4	47.5	0.59	18.44	2.59	2291.18	0.87	0.08
rand	16.46	15.44	53.59	14.51	D	–	–	–	–	–	–	–	–	–
rand	8.85	18.88	37.11	35.16	D	–	–	–	–	–	–	–	–	–
rand	38.56	26.98	14.34	20.13	A	75.9	79.4	39.3	0.66	42.87	0.96	3907.24	0.79	0.04
rand	29.38	22.66	34.31	13.65	A	74.5	76.1	36.2	0.70	34.48	1.04	4177.93	0.47	0.03
rand	25.79	27.65	39.11	7.45	A	76.2	76.6	40.4	0.71	39.57	0.87	5035.91	0.29	0.03
rand	18.86	41.11	2.79	37.25	D	–	–	–	–	–	–	–	–	–
rand	25.06	48.93	3.85	22.16	A	81.4	83.5	53.2	0.67	41.41	2.35	2704.29	0.60	0.18
rand	5.52	37.62	16.44	40.42	A	79.0	79.1	54.6	0.21	11.91	6.45	1146.50	0.76	0.01
rand	46.45	7.08	42.64	3.83	C	–	–	–	–	–	–	–	–	–
rand	17.01	19.09	21.60	42.29	C	–	–	–	–	–	–	–	–	–
rand	34.64	14.31	12.61	38.44	C	–	–	–	–	–	–	–	–	–
rand	32.06	18.88	12.96	36.10	C	–	–	–	–	–	–	–	–	–
rand	14.80	8.92	39.64	36.65	C	–	–	–	–	–	–	–	–	–
rand	20.35	28.95	27.98	22.73	A	78.9	78.3	46.4	0.51	31.38	2.65	3244.24	0.84	0.06
rand	44.06	13.36	8.06	34.52	A	73.5	76.4	40.0	0.66	24.02	1.02	3150.44	0.82	0.17

Sampling Method	Composition Labels				Film Grade	Property Labels								
	MMT (wt.%)	CNF (wt.%)	Gelatin (wt.%)	Glycerol (wt.%)		T_{Vis} (%)	T_{IR} (%)	T_{UV} (%)	RR (–)	σ_u (MPa)	ε_f (%)	E (MPa)	α (–)	β (–)
rand	32.40	8.25	18.33	41.02	D	–	–	–	–	–	–	–	–	–
rand	29.97	30.84	11.04	28.15	A	75.4	77.5	36.4	0.72	21.11	1.44	2614.94	0.83	0.05
rand	24.19	4.85	44.61	26.34	D	–	–	–	–	–	–	–	–	–
rand	2.40	32.23	40.58	24.79	A	80.8	80.4	57.5	0.15	14.95	10.36	1009.67	0.81	0.02
dist	29.36	8.67	15.86	46.12	D	–	–	–	–	–	–	–	–	–
dist	7.93	29.88	7.81	54.38	D	–	–	–	–	–	–	–	–	–
dist	47.25	13.76	36.80	2.19	A	64.4	71.4	30.9	0.75	29.42	0.39	5486.30	0.49	0.02
dist	19.17	3.38	45.10	32.34	C	–	–	–	–	–	–	–	–	–
dist	81.94	10.73	3.32	4.01	D	–	–	–	–	–	–	–	–	–
dist	50.40	36.06	2.98	10.56	C	–	–	–	–	–	–	–	–	–
dist	31.29	10.18	32.13	26.40	C	–	–	–	–	–	–	–	–	–
dist	19.76	26.61	7.20	46.43	C	–	–	–	–	–	–	–	–	–
dist	17.58	9.94	63.89	8.59	C	–	–	–	–	–	–	–	–	–
dist	15.15	27.66	39.09	18.09	A	76.0	74.7	52.7	0.60	48.70	3.65	3236.17	0.90	0.08
dist	10.36	5.36	35.78	48.50	C	–	–	–	–	–	–	–	–	–
dist	55.76	16.78	21.15	6.31	D	–	–	–	–	–	–	–	–	–
dist	8.19	51.58	7.01	33.21	A	81.5	82.9	57.1	0.21	21.00	5.31	1586.73	0.77	0.02
dist	53.60	23.79	3.58	19.02	D	–	–	–	–	–	–	–	–	–
dist	8.60	16.81	46.97	27.61	C	–	–	–	–	–	–	–	–	–
dist	39.96	33.94	2.00	24.09	C	–	–	–	–	–	–	–	–	–
dist	64.50	8.01	9.64	17.85	D	–	–	–	–	–	–	–	–	–
dist	4.95	12.63	69.17	13.26	A	76.2	82.6	50.3	0.67	17.34	10.15	3070.13	0.58	0.04
dist	3.45	61.70	17.12	17.72	A	82.3	81.9	57.4	0.10	39.98	5.18	2467.25	0.80	0.00
dist	39.36	32.11	21.98	6.55	B	–	–	–	–	–	–	–	–	–
dist	37.13	17.05	2.89	42.93	D	–	–	–	–	–	–	–	–	–
dist	19.02	20.26	29.22	31.50	D	–	–	–	–	–	–	–	–	–

Sampling Method	Composition Labels				Film Grade	Property Labels								
	MMT (wt.%)	CNF (wt.%)	Gelatin (wt.%)	Glycerol (wt.%)		T_{Vis} (%)	T_{IR} (%)	T_{UV} (%)	RR (–)	σ_u (MPa)	ε_f (%)	E (MPa)	α (–)	β (–)
dist	30.85	40.32	15.22	13.61	A	76.5	80.2	47.6	0.44	28.89	1.18	3680.45	0.71	0.21
dist	3.23	12.64	3.82	80.31	D	–	–	–	–	–	–	–	–	–
dist	61.26	2.12	3.94	32.68	D	–	–	–	–	–	–	–	–	–
dist	19.65	39.05	5.44	35.86	A	81.4	84.2	55.4	0.68	22.26	3.46	1969.62	0.87	0.09
dist	35.31	16.49	37.80	10.41	B	–	–	–	–	–	–	–	–	–
dist	7.46	32.38	32.70	27.46	A	81.2	81.4	58.7	0.48	46.16	5.85	2391.40	0.90	0.07
dist	2.49	38.84	53.67	5.00	A	76.1	73.5	55.3	0.21	58.53	2.45	3821.17	0.85	0.29
dist	2.11	40.81	42.88	14.20	A	78.0	74.2	56.5	0.32	37.95	7.10	2624.94	0.76	0.34
dist	30.29	31.28	13.72	24.72	A	75.9	77.8	46.4	0.76	29.85	1.72	2512.93	0.88	0.15
dist	56.81	2.79	31.55	8.85	D	–	–	–	–	–	–	–	–	–
dist	13.25	74.63	2.27	9.86	B	–	–	–	–	–	–	–	–	–
dist	18.59	20.72	49.93	10.76	D	–	–	–	–	–	–	–	–	–
dist	47.07	14.94	18.59	19.39	B	–	–	–	–	–	–	–	–	–
dist	8.58	20.16	6.54	64.71	D	–	–	–	–	–	–	–	–	–
dist	10.88	53.06	32.24	3.83	B	–	–	–	–	–	–	–	–	–
dist	3.03	11.04	26.10	59.83	D	–	–	–	–	–	–	–	–	–
dist	42.91	2.65	8.73	45.70	D	–	–	–	–	–	–	–	–	–
dist	10.46	41.10	2.16	46.27	A	83.5	82.4	58.1	0.57	8.90	6.49	1131.71	0.85	0.03
dist	3.23	21.35	35.27	40.15	A	81.8	79.7	58.2	0.21	25.30	0.97	3841.20	0.81	0.02
dist	2.14	43.25	14.57	40.04	A	82.5	80.2	55.9	0.10	10.61	18.80	613.47	0.67	0.12
dist	31.57	45.81	2.82	19.80	A	83.8	82.3	58.2	0.74	44.66	2.12	3155.12	0.64	0.26
dist	67.33	4.77	19.42	8.48	D	–	–	–	–	–	–	–	–	–
dist	3.52	4.68	51.35	40.45	D	–	–	–	–	–	–	–	–	–
dist	20.03	47.73	12.14	20.10	A	80.5	83.1	50.3	0.65	38.14	2.80	3458.58	0.67	0.18
dist	29.03	4.08	27.99	38.89	D	–	–	–	–	–	–	–	–	–
dist	27.12	32.93	32.79	7.16	A	78.8	78.7	47.4	0.69	63.14	1.21	6537.61	0.45	0.21

Sampling Method	Composition Labels				Film Grade	Property Labels								
	MMT (wt.%)	CNF (wt.%)	Gelatin (wt.%)	Glycerol (wt.%)		T_{Vis} (%)	T_{IR} (%)	T_{UV} (%)	RR (–)	σ_u (MPa)	ε_f (%)	E (MPa)	α (–)	β (–)
dist	16.09	36.87	41.80	5.23	A	75.8	77.1	53.7	0.70	38.56	1.18	4798.43	0.44	0.05
dist	45.32	3.30	24.30	27.07	C	–	–	–	–	–	–	–	–	–
latin	40.56	1.78	16.45	41.21	C	–	–	–	–	–	–	–	–	–
latin	19.42	42.42	1.34	36.83	A	83.0	83.6	56.6	0.67	20.48	4.40	1679.04	0.86	0.09
latin	6.56	5.81	25.02	62.61	D	–	–	–	–	–	–	–	–	–
latin	5.53	28.61	33.49	32.37	C	–	–	–	–	–	–	–	–	–
latin	28.34	13.16	45.23	13.27	D	–	–	–	–	–	–	–	–	–
latin	15.36	24.32	38.30	22.03	D	–	–	–	–	–	–	–	–	–
latin	38.29	8.02	17.90	35.80	D	–	–	–	–	–	–	–	–	–
latin	33.94	25.94	35.28	4.84	A	77.8	77.6	43.2	0.73	45.65	0.60	5884.65	0.25	0.03
latin	12.79	40.80	3.57	42.84	A	83.0	82.8	56.1	0.58	12.57	4.91	1481.99	0.86	0.03
latin	18.70	33.65	30.73	16.91	A	78.5	76.8	51.8	0.46	38.09	3.55	3465.22	0.84	0.06
latin	7.42	6.39	19.68	66.51	D	–	–	–	–	–	–	–	–	–
latin	23.21	38.37	10.24	28.18	A	78.9	81.6	49.1	0.80	35.57	2.16	2882.94	0.63	0.01
latin	26.62	31.20	4.52	37.66	A	76.9	80.6	48.9	0.62	18.19	1.76	1990.21	0.82	0.03
latin	24.05	19.42	33.44	23.10	B	–	–	–	–	–	–	–	–	–
latin	33.12	21.18	17.72	27.98	B	–	–	–	–	–	–	–	–	–
latin	16.82	47.25	31.18	4.76	A	80.2	79.7	59.4	0.64	75.39	1.59	6262.27	0.52	0.02
latin	61.00	0.81	15.09	23.10	B	–	–	–	–	–	–	–	–	–
latin	28.14	7.02	55.22	9.61	B	–	–	–	–	–	–	–	–	–
latin	25.14	34.86	28.15	11.85	A	78.6	80.3	50.1	0.67	65.08	1.58	5595.11	0.75	0.21
latin	36.71	30.01	18.23	15.05	A	73.3	78.9	40.6	0.68	39.94	0.97	4572.51	0.76	0.12
latin	42.26	42.50	4.08	11.16	A	73.8	82.9	41.4	0.72	67.01	1.59	4135.02	0.59	0.03
latin	9.86	50.68	34.25	5.22	A	77.2	78.4	59.0	0.53	71.33	1.70	6908.19	0.42	0.01
latin	37.24	36.02	6.15	20.59	A	80.2	82.3	53.0	0.78	60.09	1.55	5753.60	0.67	0.26
latin	1.02	40.03	15.21	43.74	D	–	–	–	–	–	–	–	–	–

Sampling Method	Composition Labels				Film Grade	Property Labels								
	MMT (wt.%)	CNF (wt.%)	Gelatin (wt.%)	Glycerol (wt.%)		T_{Vis} (%)	T_{IR} (%)	T_{UV} (%)	RR (–)	σ_u (MPa)	ε_f (%)	E (MPa)	α (–)	β (–)
latin	33.91	42.90	1.01	22.18	A	84.0	81.7	58.0	0.75	44.09	2.13	3054.52	0.66	0.26
latin	25.35	19.13	16.77	38.76	C	–	–	–	–	–	–	–	–	–
latin	30.98	9.40	19.34	40.28	C	–	–	–	–	–	–	–	–	–
latin	17.76	30.96	27.83	23.45	A	79.9	78.3	48.9	0.49	37.58	5.01	3621.83	0.85	0.06
latin	43.28	8.99	24.24	23.49	A	61.7	74.3	29.9	0.94	48.39	0.95	4925.07	0.84	0.28
latin	33.05	17.85	34.08	15.02	B	–	–	–	–	–	–	–	–	–
latin	25.15	15.98	30.60	28.26	D	–	–	–	–	–	–	–	–	–
latin	3.54	22.95	11.78	61.73	C	–	–	–	–	–	–	–	–	–
latin	21.04	22.52	52.11	4.33	B	–	–	–	–	–	–	–	–	–
latin	22.18	14.02	14.61	49.18	C	–	–	–	–	–	–	–	–	–
latin	35.28	17.07	31.98	15.67	B	–	–	–	–	–	–	–	–	–
latin	37.79	10.24	23.81	28.16	D	–	–	–	–	–	–	–	–	–
latin	28.76	26.61	35.92	8.71	A	76.9	77.7	38.1	0.71	40.70	1.03	4793.36	0.29	0.03
latin	6.77	59.27	32.44	1.52	B	–	–	–	–	–	–	–	–	–
latin	26.48	28.65	28.89	15.98	A	77.9	76.3	49.2	0.60	53.23	1.62	3698.31	0.77	0.21
latin	16.37	26.68	51.71	5.25	A	79.0	79.3	51.2	0.67	27.34	0.83	4620.92	0.87	0.16
latin	27.10	23.94	19.97	28.99	A	73.7	76.1	43.0	0.74	25.24	2.11	2759.53	0.86	0.15
latin	13.98	23.27	30.19	32.56	C	–	–	–	–	–	–	–	–	–
latin	19.36	14.43	31.20	35.01	C	–	–	–	–	–	–	–	–	–
latin	23.98	39.43	30.79	5.80	A	79.4	77.9	56.7	0.71	58.40	1.30	5429.33	0.19	0.02
latin	12.27	1.88	44.22	41.63	C	–	–	–	–	–	–	–	–	–
latin	12.13	32.73	10.85	44.30	C	–	–	–	–	–	–	–	–	–
latin	31.75	45.68	15.22	7.35	A	77.4	82.4	52.0	0.39	41.48	1.45	4269.88	0.69	0.21
latin	2.17	23.31	60.18	14.33	A	78.5	80.4	56.1	0.35	25.70	11.58	2337.73	0.83	0.03
latin	32.99	30.41	26.86	9.75	A	77.1	78.8	52.8	0.69	51.63	0.91	4833.81	0.67	0.21
latin	38.32	13.52	6.64	41.52	D	–	–	–	–	–	–	–	–	–

Sampling Method	Composition Labels				Film Grade	Property Labels								
	MMT (wt.%)	CNF (wt.%)	Gelatin (wt.%)	Glycerol (wt.%)		T_{Vis} (%)	T_{IR} (%)	T_{UV} (%)	RR (–)	σ_u (MPa)	ε_f (%)	E (MPa)	α (–)	β (–)
human	50.00	50.00	0.00	0.00	B	–	–	–	–	–	–	–	–	–
human	0.00	50.00	0.00	50.00	A	84.5	84.4	62.1	0.07	12.45	9.87	954.69	0.76	0.06
human	0.00	50.00	50.00	0.00	A	79.0	81.0	61.1	0.10	41.20	1.00	2263.70	0.56	0.02
human	50.00	0.00	0.00	50.00	D	–	–	–	–	–	–	–	–	–
human	50.00	0.00	50.00	0.00	A	–	–	–	–	–	–	–	–	–
human	0.00	0.00	50.00	50.00	D	–	–	–	–	–	–	–	–	–
human	33.33	33.33	0.00	33.33	A	77.2	78.9	43.3	0.68	24.48	3.01	2030.61	0.85	0.03
human	33.33	0.00	33.33	33.33	B	–	–	–	–	–	–	–	–	–
human	0.00	33.33	33.33	33.33	A	78.9	77.0	61.7	0.11	22.02	15.34	1511.48	0.85	0.02
human	33.33	33.33	33.33	0.00	A	73.4	78.1	43.3	0.69	29.61	1.32	3785.68	0.64	0.21
human	25.00	50.00	25.00	0.00	A	–	–	–	–	–	–	–	–	–
human	50.00	25.00	25.00	0.00	A	–	–	–	–	–	–	–	–	–
human	25.00	25.00	25.00	25.00	C	–	–	–	–	–	–	–	–	–
human	25.00	25.00	50.00	0.00	D	–	–	–	–	–	–	–	–	–
human	10.00	40.00	40.00	10.00	D	–	–	–	–	–	–	–	–	–
human	0.00	50.00	40.00	10.00	D	–	–	–	–	–	–	–	–	–
human	0.00	40.00	50.00	10.00	D	–	–	–	–	–	–	–	–	–
human	32.00	32.00	32.00	4.00	D	–	–	–	–	–	–	–	–	–
human	40.00	40.00	15.00	5.00	A	75.8	80.3	42.6	0.60	49.57	0.90	7927.64	0.60	0.17
human	30.00	30.00	30.00	10.00	B	–	–	–	–	–	–	–	–	–
human	0.00	48.00	48.00	4.00	A	77.8	79.4	61.4	0.11	46.68	0.28	7356.38	0.56	0.02
human	32.00	32.00	32.00	4.00	A	76.9	77.5	49.9	0.68	34.36	0.21	8702.57	0.55	0.21
human	16.00	40.00	40.00	4.00	A	80.6	79.1	59.3	0.70	25.93	0.15	8033.40	0.47	0.05
human	33.00	33.00	33.00	1.00	A	76.3	79.4	47.4	0.69	25.47	0.16	7418.98	0.64	0.21
human	35.00	35.00	25.00	5.00	A	76.3	79.8	48.0	0.68	33.61	0.17	10298.08	0.66	0.21
human	5.00	60.00	30.00	5.00	A	82.5	80.7	62.9	0.24	96.78	0.41	10662.42	0.70	0.42

Sampling Method	Composition Labels				Film Grade	Property Labels								
	MMT (wt.%)	CNF (wt.%)	Gelatin (wt.%)	Glycerol (wt.%)		T_{Vis} (%)	T_{IR} (%)	T_{UV} (%)	RR (–)	σ_u (MPa)	ε_f (%)	E (MPa)	α (–)	β (–)
human	3.00	70.00	25.00	2.00	A	83.8	80.8	65.2	0.14	55.36	0.48	10875.65	0.60	0.41
human	16.00	60.00	20.00	4.00	A	83.4	85.6	66.5	0.57	48.48	0.61	9785.45	0.57	0.03
human	0.00	49.00	49.00	2.00	A	77.8	79.6	60.6	0.10	37.94	0.17	6413.19	0.56	0.02
human	5.00	35.00	60.00	0.00	A	79.5	79.2	62.5	0.58	12.65	0.32	4927.28	0.41	0.04
human	8.00	76.00	8.00	8.00	B	–	–	–	–	–	–	–	–	–
human	76.00	8.00	8.00	8.00	B	–	–	–	–	–	–	–	–	–
human	8.00	8.00	8.00	76.00	D	–	–	–	–	–	–	–	–	–
human	8.00	8.00	76.00	8.00	B	–	–	–	–	–	–	–	–	–
human	10.00	20.00	20.00	50.00	D	–	–	–	–	–	–	–	–	–
human	20.00	20.00	10.00	50.00	D	–	–	–	–	–	–	–	–	–
human	20.00	10.00	20.00	50.00	D	–	–	–	–	–	–	–	–	–
human	10.00	20.00	50.00	20.00	C	–	–	–	–	–	–	–	–	–
human	20.00	20.00	50.00	10.00	A	77.8	78.7	46.8	0.69	25.78	0.37	5738.21	0.79	0.16
human	20.00	10.00	50.00	20.00	C	–	–	–	–	–	–	–	–	–
human	30.00	20.00	40.00	10.00	C	–	–	–	–	–	–	–	–	–
human	15.00	25.00	50.00	10.00	D	–	–	–	–	–	–	–	–	–
human	20.00	30.00	25.00	25.00	A	79.6	75.9	49.3	0.59	33.44	0.74	4266.66	0.73	0.48
human	25.00	10.00	35.00	30.00	C	–	–	–	–	–	–	–	–	–
human	25.00	15.00	40.00	20.00	A	–	–	–	–	–	–	–	–	–
human	10.00	35.00	35.00	20.00	A	77.5	74.8	54.1	0.64	39.06	1.21	3458.05	0.74	0.08
human	30.00	30.00	35.00	5.00	A	75.7	76.6	36.0	0.70	42.96	0.23	9383.28	0.24	0.03
human	40.00	20.00	25.00	15.00	B	–	–	–	–	–	–	–	–	–
human	25.00	25.00	20.00	30.00	C	–	–	–	–	–	–	–	–	–
human	30.00	10.00	35.00	25.00	C	–	–	–	–	–	–	–	–	–

Table S7. Composition labels of 11 model-predicted all-natural nanocomposite.

Sample Number	Composition Labels			
	MMT (wt.%)	CNF (wt.%)	Gelatin (wt.%)	Glycerol (wt.%)
#1	5.9	36.0	1.1	56.9
#2	51.2	27.9	19.3	1.6
#3	71.6	2.0	24.9	1.4
#4	9.0	50.9	11.6	28.5
#5	51.2	27.9	19.3	1.6
#6	71.6	2.0	24.9	1.4
#7	9.0	50.9	11.6	28.5
#8	29.5	28.1	12.4	30.0
#9	2.6	41.4	6.4	49.6
#10	45.5	34.7	6.5	13.3
#11	52.0	1.9	22.8	23.3

Table S8. Comparison of model-suggested MMT-rich and CNF-rich nanocomposites with high σ_u.

Sample Name		MMT (wt%)	CNF (wt%)	Gelatin (wt%)	Glycerol (wt%)	σ_u (MPa)
MMT-Rich Cluster Center in Fig. S20a		64.2%	6.7%	23.8%	5.3%	114 ± 18
Other Model-Suggested Nanocomposites Near MMT-Rich Cluster Center in Fig. S19b,c	1	66.1%	3.5%	20.9%	9.5%	106 ± 3
	2	65.0%	3.0%	25.0%	6.9%	97 ± 7
	3	63.0%	2.6%	30.7%	3.7%	88 ± 7
	4	59.4%	5.7%	31.0%	3.9%	71 ± 8
	5	66.1%	9.1%	19.7%	5.2%	97 ± 18
CNF-Rich Cluster Center in Fig. S20a		3.7%	61.8%	28.4%	6.1%	98 ± 7
Other Model-Suggested Nanocomposites Near CNF-Rich Cluster Center in Fig. S19b,d	1	4.8%	59.4%	28.3%	7.6%	90 ± 8
	2	3.6%	62.0%	29.0%	5.4%	84 ± 12
	3	2.0%	65.3%	27.7%	5.0%	99 ± 14
	4	4.6%	58.9%	29.0%	7.5%	81 ± 4
	5	4.8%	59.3%	28.0%	7.9%	91 ± 15

Table S9. Grades of 90 nanocomposites with different MMT/CNF/chitosan/gelatin/ glycerol ratios during the model expansion process.

"*A*-grades" refer to the conditions that the nanocomposite films were detachable and flat. "*B*-grades" refer to the conditions that the nanocomposite films were detachable yet curved. "*C*-grades" refer to the conditions that the nanocomposite films were detachable yet fractured. "*D*-grades" refer to the conditions that the nanocomposite films were non-detachable.

ID	MMT/CNF/Chitosan/Gelatin/Glycerol Ratio					Grade
	MMT	CNF	Chitosan	Gelatin	Glycerol	
1	17.82	3.62	39.86	36.38	2.33	B
2	4.61	24.25	3.92	19.16	48.04	D
3	4.20	26.77	42.77	26.26	0.00	A
4	45.06	0.00	27.02	27.92	0.00	D
5	5.72	2.46	84.26	2.41	5.16	D
6	2.09	5.61	18.11	71.78	2.40	D
7	35.69	28.18	3.42	23.99	8.72	D
8	22.12	0.00	51.09	26.79	0.00	B
9	45.38	23.16	3.85	15.20	12.40	B
10	2.45	7.32	68.81	3.58	17.83	D
11	14.96	0.00	75.17	4.43	5.45	A
12	17.09	3.05	61.70	15.82	2.34	D
13	16.73	2.11	30.24	43.29	7.62	D
14	0.00	20.86	35.18	32.36	11.60	D
15	9.04	0.00	6.95	59.55	24.46	A
16	2.64	18.79	12.48	63.80	2.30	D
17	23.93	6.13	17.68	21.40	30.86	D
18	12.64	0.00	24.40	62.96	0.00	A
19	2.54	34.77	43.35	15.53	3.81	D
20	2.51	51.66	6.38	37.32	2.13	D
21	57.49	8.70	5.69	23.01	5.11	D
22	56.37	0.00	4.03	39.60	0.00	C
23	2.43	32.97	50.83	5.49	8.28	D
24	2.27	16.67	27.10	51.01	2.95	D
25	2.08	25.76	64.59	5.10	2.46	D
26	9.08	0.00	67.64	10.84	12.45	B
27	0.00	28.98	26.75	44.27	0.00	A
28	2.23	32.49	2.09	32.87	30.32	D
29	0.00	27.18	34.56	2.39	35.88	D
30	30.78	18.77	7.87	13.73	28.85	D
31	0.00	30.22	51.42	12.81	5.55	A
32	16.85	2.35	44.65	24.39	11.77	D
33	3.25	19.40	51.86	22.67	2.83	D
34	66.93	13.31	2.21	8.03	9.52	D
35	5.05	8.11	60.69	21.40	4.75	D
36	2.11	0.00	32.14	33.17	32.57	D
37	9.71	30.00	6.88	48.83	4.58	D
38	72.15	7.05	3.85	7.80	9.15	B
39	7.67	2.04	41.69	30.51	18.09	D
40	48.74	4.78	5.21	24.06	17.20	D
41	0.00	0.00	20.50	47.66	31.84	A
42	0.00	4.72	49.34	21.00	24.94	A
43	9.59	0.00	38.27	21.29	30.86	D
44	7.50	0.00	4.05	83.47	4.97	D
45	0.00	37.11	35.73	27.17	0.00	A
46	50.21	28.29	2.72	13.50	5.28	D
47	2.51	3.98	4.70	72.48	16.33	D
48	6.86	6.87	28.66	0.00	57.61	D
49	31.09	6.04	5.77	57.10	0.00	D
50	0.00	10.39	3.92	43.69	41.99	D
51	0.00	22.34	34.11	43.55	0.00	D
52	2.20	21.09	43.16	2.31	31.24	D

ID	MMT/CNF/Chitosan/Gelatin/Glycerol Ratio					Grade
	MMT	CNF	Chitosan	Gelatin	Glycerol	
53	24.98	32.69	3.95	33.23	5.15	C
54	8.34	2.17	22.43	45.84	21.22	D
55	2.14	2.31	57.93	10.68	26.94	D
56	2.16	17.44	48.38	17.90	14.11	D
57	0.00	0.00	16.25	0.00	83.75	C
58	3.48	24.97	44.15	27.40	0.00	D
59	2.77	5.28	8.17	80.46	3.32	D
60	0.00	4.08	42.16	17.27	36.49	D
61	2.20	37.47	19.88	38.22	2.23	D
62	43.76	0.00	3.87	19.47	32.90	C
63	4.20	32.32	14.65	48.83	0.00	A
64	5.72	15.27	53.93	11.86	13.23	D
65	2.08	6.74	15.92	45.54	29.72	D
66	7.89	2.45	27.64	58.99	3.03	D
67	2.67	12.50	39.42	2.66	42.75	D
68	2.47	14.47	71.60	4.19	7.27	D
69	4.88	2.88	2.80	86.88	2.56	B
70	0.00	18.09	16.60	36.17	29.14	D
71	6.46	2.11	52.83	6.30	32.29	D
72	0.00	2.02	40.74	39.13	18.11	D
73	48.71	0.00	22.40	6.53	22.35	D
74	0.00	28.28	23.25	24.57	23.90	A
75	2.58	2.60	41.90	49.02	3.90	D
76	2.65	36.25	35.19	23.17	2.74	D
77	2.36	23.89	36.71	11.91	25.13	D
78	17.41	35.51	2.07	42.81	2.20	D
79	40.61	3.76	4.87	47.99	2.77	D
80	0.00	4.42	48.77	0.00	46.82	A
81	0.00	0.00	50.77	49.23	0.00	A
82	3.16	31.86	2.39	2.87	59.73	D
83	43.34	3.02	2.82	23.87	26.94	D
84	7.31	10.12	33.13	22.94	26.50	A
85	0.00	0.00	13.18	80.47	6.35	A
86	2.07	6.42	43.84	45.42	2.25	D
87	16.00	45.73	2.17	33.11	2.98	D
88	0.00	27.51	46.18	0.00	26.30	A
89	2.90	2.27	34.96	57.84	2.04	D
90	0.00	29.25	23.71	16.30	30.73	D

Table S10. Dataset for the prediction model during the model expansion process.

Composition Labels					Property Labels								
MMT (wt.%)	CNF (wt.%)	Chitosan (wt.%)	Gelatin (wt.%)	Glycerol (wt.%)	T_{Vis} (%)	T_{IR} (%)	T_{UV} (%)	RR (–)	σ_u (MPa)	ε_f (%)	E (MPa)	α (–)	β (–)
2.19	43.98	2.17	49.52	2.13	57.9	60.0	38.3	0.11	7.74	0.64	3296.41	0.11	0.56
2.71	4.12	76.38	8.13	8.66	78.9	79.5	51.4	0.60	22.19	34.47	623.77	0.60	0.72
54.24	2.42	5.04	33.07	5.23	55.3	61.9	19.6	0.83	13.02	1.11	4953.14	0.83	0.31
34.06	44.38	2.50	6.72	12.34	75.1	76.1	47.9	0.47	41.88	1.62	6422.24	0.47	0.66
4.02	62.61	2.55	28.71	2.11	66.1	63.0	40.4	0.16	19.60	1.45	3516.00	0.16	0.68
45.59	4.80	4.16	40.02	5.43	61.5	67.1	27.8	0.69	6.77	0.93	2306.65	0.69	0.69
2.19	31.73	2.42	4.77	58.89	61.9	55.7	38.4	0.20	2.43	3.86	164.53	0.20	0.80
38.25	37.83	2.04	21.88	0.00	44.0	56.6	16.1	0.68	3.81	1.43	1255.24	0.68	0.65
17.82	3.62	39.86	36.38	2.33	74.4	70.5	50.5	0.80	20.62	5.97	1730.03	0.80	0.63
2.71	2.41	67.47	24.70	2.71	78.8	78.3	52.8	0.64	23.11	5.95	1759.46	0.64	0.76
11.47	3.44	14.21	68.38	2.50	74.7	78.5	59.1	0.73	9.44	4.53	1138.14	0.73	0.74
35.61	3.26	4.54	30.53	26.07	72.9	77.0	42.3	0.57	2.82	1.70	618.51	0.57	0.78
2.54	34.77	43.35	15.53	3.81	81.5	81.7	58.4	0.44	38.52	23.02	2001.91	0.44	0.74
6.58	2.39	59.68	28.01	3.34	80.5	80.9	53.1	0.71	57.08	7.37	3626.87	0.71	0.46
2.45	7.32	68.82	3.58	17.83	80.3	80.9	56.5	0.55	44.22	27.11	1686.16	0.55	0.87
8.43	2.42	12.73	57.39	19.03	57.1	73.6	68.8	0.53	8.91	22.64	236.45	0.53	0.86
38.66	11.63	0.72	14.71	34.28	68.3	74.0	36.0	0.64	27.69	0.67	6424.57	0.64	0.81
5.31	23.13	49.12	3.79	18.66	78.9	79.8	59.2	0.52	46.93	25.91	2053.49	0.52	0.82
16.85	2.35	44.65	24.39	11.77	76.4	76.5	51.9	0.88	41.97	25.70	1853.39	0.88	0.77
3.25	19.40	51.85	22.67	2.83	80.4	80.5	49.6	0.51	52.21	16.74	3215.70	0.51	0.61
0.00	2.02	40.74	39.13	18.11	81.4	84.1	54.3	0.48	41.65	33.49	1312.13	0.48	0.84

Composition Labels					Property Labels								
18.81	18.29	4.53	35.08	23.30	73.1	73.6	38.2	0.60	18.56	3.27	2550.10	0.60	0.88
0.00	18.09	16.60	36.17	29.14	80.2	79.7	61.2	0.25	22.42	25.60	1363.60	0.25	0.82
2.27	16.67	27.10	51.01	2.95	80.5	80.1	61.1	0.56	37.04	2.40	2908.22	0.56	0.41
66.93	13.31	2.21	8.03	9.52	43.4	58.5	14.9	0.92	22.86	0.57	6240.77	0.92	0.28
2.90	2.27	34.96	57.83	2.04	84.2	81.8	67.4	0.69	41.51	2.35	2936.27	0.69	0.52
4.95	2.58	52.89	18.64	20.94	83.2	81.5	62.8	0.50	37.35	15.66	2361.94	0.50	0.85
2.67	12.50	39.42	2.66	42.75	83.6	84.2	66.3	0.38	42.65	18.98	2466.33	0.38	0.83
2.10	4.28	35.71	49.12	8.79	81.9	85.2	54.9	0.55	24.59	17.00	1158.14	0.55	0.83
39.62	44.55	1.54	8.89	5.41	79.7	78.6	54.5	0.61	66.43	1.14	12380.55	0.61	0.58
2.14	2.31	57.93	10.68	26.94	79.8	77.5	53.4	0.54	18.82	35.46	568.88	0.54	0.87
24.61	23.48	2.07	6.80	43.04	77.3	76.0	49.9	0.63	15.47	2.57	1663.87	0.63	0.81
2.09	5.61	18.11	71.79	2.40	79.5	76.9	61.0	0.69	22.70	2.23	2097.68	0.69	0.50
5.05	8.11	60.69	21.40	4.75	77.2	76.4	51.1	0.60	26.28	6.41	1810.70	0.60	0.77
43.34	3.02	2.82	23.87	26.94	59.9	67.3	25.0	0.84	19.74	0.38	9693.21	0.84	0.73
43.34	11.17	3.97	5.90	35.62	67.9	71.3	32.3	0.64	25.08	1.00	5599.63	0.64	0.82
57.49	8.70	5.69	23.01	5.11	37.3	54.4	6.9	0.88	36.51	0.55	9353.52	0.88	0.64
2.14	13.48	45.49	38.89	0.00	78.7	77.2	53.9	0.62	46.21	4.12	2872.40	0.62	0.66
17.41	35.51	2.07	42.81	2.20	64.3	64.8	36.3	0.68	10.43	0.54	3640.19	0.68	0.69
40.61	3.76	4.87	47.99	2.77	61.9	65.3	31.6	0.78	12.86	0.80	5081.61	0.78	0.54
2.10	2.51	22.29	53.34	19.76	81.0	77.4	63.8	0.53	7.34	25.71	164.03	0.53	0.85
30.08	3.38	2.01	43.55	20.98	75.8	76.5	48.5	0.88	19.52	2.39	2492.92	0.88	0.72
35.69	28.18	3.42	23.99	8.72	59.2	63.5	26.3	0.78	43.45	0.86	9095.29	0.78	0.34

Table S11. Summary of inverse design requests for targeted plastic products and model-suggested compositions.

Targeted Plastic Product	Property Criteria			Model-Suggested Composition Labels					Tested Property*
	Optical Property	Fire-Resistant Property	Mechanical Property	MMT (wt.%)	CNF (wt.%)	Chitosan (wt.%)	Gelatin (wt.%)	Glycerol (wt.%)	
Transparent Badge Holder	T_{Vis} > 90%	–	–	0.0	71.9	–	17.6	10.4	T_{Vis} = 92.1 ± 0.6%
Clear File Folder	T_{Vis} > 80%	–	ε_f > 5%	2.3	1.5	42.8	51.7	1.7	T_{Vis} = 83.1 ± 0.5%<br>ε_f = 5.27± 0.86%
Transparent Shopping Bag	T_{Vis} > 75%	–	σ_u > 100 MPa	6.0	48.8	–	32.2	12.0	T_{Vis} = 77.9 ± 1.2%<br>σ_u = 126.23 ± 37.08 MPa
Translucent Lamp Shading	50% > T_{Vis} > 70%	–	σ_u > 80 MPa	26.9	23.2	–	40.1	9.7	T_{Vis} = 69.7 ± 1.5%<br>σ_u = 83.86 ± 5.23 MPa
Transparent Air Pillow	T_{Vis} > 80%	–	σ_u > 25 MPa<br>ε_f > 25%	0.0	20.8	55.1	8.4	15.7	T_{Vis} = 81.0 ± 0.4%<br>σ_u = 28.40 ± 6.19 MPa<br>ε_f = 28.10 ± 3.89%
Transparent, Non-Flammable Battery Packaging	T_{Vis} > 75%	RR > 0.7	σ_u > 100 MPa<br>ε_f > 5%	35.5	34.7	–	11.7	18.1	T_{Vis} = 78.4 ± 2.5%<br>RR = 0.72 ± 0.03<br>σ_u = 102.42 ± 10.31 MPa<br>ε_f = 6.16 ± 2.58%
UV-Blocking, Non-Flammable Chemical Packaging	T_{Vis} < 50%<br>T_{UV} < 5%	RR > 0.9	σ_u > 100 MPa<br>ε_f > 3%	64.9	12.1	–	14.3	7.7	T_{Vis} = 34.5 ± 2.1%<br>T_{UV} = 3.6 ± 1.6%<br>RR = 0.96 ± 0.02<br>σ_u = 115.31 ± 18.73 MPa<br>ε_f = 3.61 ± 0.29%

*For instance, for the replacement of shopping bag, the all-natural substitutes were required to be highly transparent and mechanically strong (i.e., > 90% and > 100 MPa). For the replacement of chemical packaging, the all-natural substitutes were specialized to have low UV transmittances, high fire retardancy, and strong tensile strengths (i.e., > 90%, > 0.9, >5%, and > 100 MPa). By inputting these design criteria, the champion model was able to automate the inverse design to find the most suitable all-natural substitutes *via* clustering analyses.

Table S12. Comparison of our all-natural plastic substitutes with the state-of-the-art works.

Natural Components	Preparation Method	Design Method	σ_u (MPa)	E (GPa)	T_{UV} (365 nm, %)	T_{Vis} (550 nm, %)	T_{IR} (950 nm, %)	RR (–)	Ref.
MMT/CNF/Gelatin/Glycerol	Cast Drying/Ionic Crosslinking /Hot Pressing	AI/ML Predictions	1–520	0.5–71.7	2%–76%	20%–93%	54%–90%	0.00–1.00	**This Work**
Soy Protein/Glycerol	Cast Drying	Design of Experiments	19.1	0.23	–	93.7%	–	–	18
Polylactic Acid	Injection	Design of Experiments	44.1	2.69	–	–	–	–	19
Microcrystalline Cellulose	Cast Drying	Design of Experiments	83.6	3.29	–	~83%	–	–	20
Microcrystalline Cellulose	Cast Drying	Design of Experiments	93.9	3.62	–	>85%	–	–	21
Microcrystalline Cellulose/ Poly(methyl methacrylate)	Cast Drying	Design of Experiments	71.6	3.59	–	–	–	–	22
Cellulose/Hydrolyzed Lignin/Xylan	Cast Drying	Design of Experiments	39.9	1.79	–	93%	–	–	23
Cellulose/Polyhydroxylated Fatty Acid Ester	Acylation/Air Drying	Design of Experiments	55	3.2	–	–	–	–	24
Edible Vegetable Waste	Cast Drying	Design of Experiments	51	4.1	–	–	–	–	25
Cellulose Acetate/Triethyl Citrate	Extrusion/Compression Molding	Design of Experiments	26	1.3	–	–	–	–	26

Natural Components	Preparation Method	Design Method	σ_u (MPa)	E (GPa)	T_{UV} (365 nm, %)	T_{Vis} (550 nm, %)	T_{IR} (950 nm, %)	RR (–)	Ref.
Starch/Cellulose Nanocrystals	Cast Drying	Design of Experiments	26.0	0.89	–	–	–	–	27
Cellulose Nanofibers	Crosslinking/Air Drying	Design of Experiments	303	23.8	–	90%	–	–	28
Cellulose Acetate/Triethyl Citrate/Clay	Melt Compounding	Design of Experiments	120	6	–	–	–	–	29
Cellulose/Rectorite	Cast Drying	Design of Experiments	123.0	5.36	2.5%	15%	–	–	30
Calcium Phosphate Nanofibers	Hierarchical Assembly	Design of Experiments	23.6	0.78	–	–	–	–	31
Gluten/Glycerol	Thermo-Molding	Design of Experiments	7.35	0.26	–	–	–	–	32
Cellulose/Polypropylene	Compression Molding	Design of Experiments	26.0	0.89	–	–	–	–	33
Aramid Nanofibers/ Rectorite	Cast Drying	Design of Experiments	232.0	3.81	–	–	–	–	34

Table S13. Summary of data-driven influential components *via* different data analysis methods.

Data Analysis Method	Property Labels								
	T_{vis} (%)	T_{IR} (%)	T_{UV} (%)	RR (–)	σ_u (MPa)	ε_f (%)	E (MPa)	α (–)	β (–)
Spearman's ρ Analysis	CNF (+) MMT (–)	CNF (+) MMT (–)	CNF (+) MMT (–)	CNF (–) MMT (+)	No Significant Correlations	CNF (+) Glycerol (+) MMT (–)	Glycerol (–) MMT (+)	–	–
SHAP Analysis	CNF (+) MMT (–)	CNF (+) MMT (–)	CNF (+) MMT (–)	CNF (–) MMT (+)	Glycerol (–) CNF (+) MMT (+)	Glycerol (+) MMT (–)	Glycerol (–) MMT (+)	–	–

Table S14. Comparison of our AI/ML framework with the literature.

Ref.	Controllable Parameters	Optimization Targets	Multi-Property Optimization	Sampling Method	Sample Preparation and Characterization Tools/Platforms	Data Collection Rate	Machine Learning Algorithms
35	Fuel-to-oxidizer ratio, fuel blend, total concentration, anneal temperature (4 DOFs)	High-conductivity palladium films at low anneal temperature (2 property labels)	Yes	Bayesian optimization (w/ qEHVI acquisition function)	Ada: Precision 4-axis laboratory robot (N9)/6-axis collaborative robot (UR5e)	2 data per hour	Gaussian process regression
36	Column count, column outer radius, column thickness, twisted angle (4 DOFs)	Mechanical structures with high compression toughness (1 property label)	No	Bayesian optimization	BEAR: 3D printers/robotic arm/scale/universal testing machine	64 data per day	Gaussian process regression
37	Reagent injection sequence, reaction time and volume (>40 DOFs)	Hetero-nanostructures with high λ_{AP}, R_{PV}, I_{PL} (3 property labels)	Yes	Reinforcement learning	AlphaFlow: A self-driven fluidic lab consisting of reagent injection, droplet oscillation, optical sampling, phase separation, and waste collection.	2 data per hour	Reinforcement learning
**This work**	**MMT, CNF, gelatin, glycerol loadings (3 DOFs)**	**All-natural plastic substitutes with customized optical, fire-resistant, mechanical properties (9 property labels)**	**Yes (Optical, fire-resistant, mechanical properties)**	**Active learning (w/ *A score* acquisition function)**	**Automated pipetting robot (i.e., OT-2)/UV-vis spectrophotometer/fire tester/universal testing machine**	10 data per 2 days	Support-vector machine /artificial neural network /data augmentation

Supporting References

1 Tu, H., Zhu, M., Duan, B. & Zhang, L. Recent progress in high-strength and robust regenerated cellulose materials. *Adv. Mater.* **33**, 2000682 (2021).

2 Ryder, K., Ali, M. A., Billakanti, J. & Carne, A. Evaluation of dairy co-product containing composite solutions for the formation of bioplastic films. *J. Polym. Environ.* **28**, 725–736 (2020).

3 Lamm, M. E. *et al.* Recent advances in functional materials through cellulose nanofiber templating. *Adv. Mater.* **33**, 2005538 (2021).

4 Watanabe, M. *et al.* Addition of glycerol enhances the flexibility of gelatin hydrogel sheets; application for in utero tissue engineering. *J. Biomed. Mater. Res., Part B* **109**, 921–931 (2021).

5 Awad, M. & Khanna, R. *Efficient learning machines: Theories, concepts, and applications for engineers and system designers*. p. 39–66 (Springer nature, Apress, 2015).

6 Head, T., Kumar, M., Nahrstaedt, H., Louppe, G. & Shcherbatyi, I. Scikit-optimize/scikit-optimize: v0.9.0. (2021).

7 Schober, P., Boer, C., Schwarte, L. A. J. A. & analgesia. Correlation coefficients: Appropriate use and interpretation. *Anesth. Analg.* **126**, 1763–1768 (2018).

8 Zhu, M. *et al.* Anisotropic, transparent films with aligned cellulose nanofibers. *Adv. Mater.* **29**, 1606284 (2017).

9 Aulin, C., Salazar-Alvarez, G. & Lindstrom, T. High strength, flexible and transparent nanofibrillated cellulose-nanoclay biohybrid films with tunable.

10 E, S. *et al.* Ternary synergistic strengthening and toughening of bio-inspired TMPO-oxidized cellulose nanofibers/borax/polyvinyl alcohol composite film with high transparency. *ACS Sustainable Chem. Eng.* **8**, 15661–15669 (2020).

11 Kumar, P., Sandeep, K. P., Alavi, S., Truong, V. D. & Gorga, R. E. Effect of type and content of modified montmorillonite on the structure and properties of bio-nanocomposite films based on soy protein isolate and montmorillonite. *J. Food Sci.* **75**, N46-N56 (2010).

12 Wang, Z., Wang, H. & Cates, M. E. Effective elastic properties of solid clays. *GEOPHYSICS* **66**, 428–440 (2001).

13 Du, J., Zhou, A., Lin, X., Bu, Y. & Kodikara, J. Revealing Expansion Mechanism of Cement-Stabilized Expansive Soil with Different Interlayer Cations through Molecular Dynamics Simulations. *J. Phys. Chem. C* **124**, 14672–14684 (2020).

14 Wang, S. *et al.* Super-Strong, Super-stiff macrofibers with aligned, long bacterial cellulose nanofibers. *Adv. Mater.* **29**, 1702498 (2017).

15 Fang, Z. *et al.* Critical role of degree of polymerization of cellulose in super-strong nanocellulose films. *Matter* **2**, 1000–1014 (2020).

16 Quirós, M., Gražulis, S., Girdzijauskaitė, S., Merkys, A. & Vaitkus, A. Using SMILES strings for the description of chemical connectivity in the crystallography open database. *J. Cheminf.* **10**, 23 (2018).

17 O'Boyle, N. M. Towards a universal SMILES representation – A standard method to generate canonical smiles based on the InChI. *J. Cheminf.* **4**, 22 (2012).

18 Kamada, A. *et al.* Controlled self-assembly of plant proteins into high-performance multifunctional nanostructured films. *Nat.Commun.* **12**, 3529 (2021).

19 Cakir, S., Aycicek, M. & Akinci, A. Investigation of the mechanical and physical properties of PLA produced by injection molding for matrix material of polymer composites. *Mater. Sci. Adv. Compos. Mater.* **2**, 1–7 (2018).

20 Guzman-Puyol, S. *et al.* Transparent and robust all-cellulose nanocomposite packaging materials prepared in a mixture of trifluoroacetic acid and trifluoroacetic anhydride. *Nanomaterials* **9** (2019).

21 Guzman-Puyol, S. *et al.* Transparent, UV-blocking, and high barrier cellulose-based bioplastics with naringin as active food packaging materials. *Int. J. Biol. Macromol.* **209**, 1985–1994 (2022).

22 Tran, T. N. *et al.* Transparent and flexible amorphous cellulose-acrylic hybrids. *Chem. Eng. J.* **287**, 196–204 (2016).

23 Tedeschi, G. *et al.* Multifunctional bioplastics inspired by wood composition: Effect of hydrolyzed lignin addition to xylan–cellulose matrices. *Biomacromolecules* **21**, 910–920 (2020).

24 Heredia-Guerrero, J. A. *et al.* Cellulose-polyhydroxylated fatty acid ester-based bioplastics with tuning properties: Acylation *via* a mixed anhydride system. *Carbohydr. Polym.* **173**, 312–320 (2017).

25 Bayer, I. S. *et al.* Direct transformation of edible vegetable waste into bioplastics. *Macromolecules* **47**, 5135–5143 (2014).

26 Mohanty, A. K., Wibowo, A., Misra, M. & Drzal, L. T. Development of renewable resource–based cellulose acetate bioplastic: Effect of process engineering on the performance of cellulosic plastics. *Polym. Eng. Sci.* **43**, 1151–1161 (2003).

27 Agustin, M. B., Ahmmad, B., Alonzo, S. M. M. & Patriana, F. M. Bioplastic based on starch and cellulose nanocrystals from rice straw. *J. Reinf. Plast. Compos.* **33**, 2205–2213 (2014).

28 Lee, K. *et al.* Double-crosslinked cellulose nanofiber based bioplastic films for practical applications. *Carbohydr. Polym.* **260**, 117817 (2021).

29 Park, H.-M., Misra, M., Drzal, L. T. & Mohanty, A. K. "Green" nanocomposites from cellulose acetate bioplastic and clay: Effect of eco-friendly triethyl citrate plasticizer. *Biomacromolecules* **5**, 2281–2288 (2004).

30 Jin, L. *et al.* Fabrication of cellulose/rectorite composite films for sustainable packaging. *Int. J. Biol. Macromol.* **224**, 1471–1477 (2023).

31 Yu, Y. *et al.* A flexible and degradable hybrid mineral as a plastic substitute. *Adv. Mater.* **34**, 2107523 (2022).

32 Song, Y. & Zheng, Q. Improved tensile strength of glycerol-plasticized gluten bioplastic containing hydrophobic liquids. *Bioresour. Technol.* **99**, 7665–7671 (2008).

33 Hassan, M. L., Mathew, A. P., Hassan, E. A., Fadel, S. M. & Oksman, K. Improving cellulose/polypropylene nanocomposites properties with chemical modified bagasse nanofibers and maleated polypropylene. *J. Reinf. Plast. Compos.* **33**, 26–36 (2013).

34 Pan, X.-F. *et al.* Large-scale production of rectorite nanosheets and their co-assembly with aramid nanofibers for high-performance electrical insulating nanopapers. *Adv. Mater.* **34**, 2206855 (2022).

35 MacLeod, B. P. *et al.* A self-driving laboratory advances the Pareto front for material properties. *Nat.Commun.* **13**, 995 (2022).

36 Gongora, A. E. *et al.* A Bayesian experimental autonomous researcher for mechanical design. *Sci. Adv.* **6**, eaaz1708.

37 Volk, A. A. *et al.* AlphaFlow: Autonomous discovery and optimization of multi-step chemistry using a self-driven fluidic lab guided by reinforcement learning. *Nat.Commun.* **14**, 1403 (2023).
